# Two-year outcomes after selective early treatment of patent ductus arteriosus with ibuprofen in preterm babies: follow-up of Baby-OSCAR–a randomised controlled trial

**DOI:** 10.1016/j.eclinm.2025.103424

**Published:** 2025-08-20

**Authors:** Samir Gupta, Heather O'Connor, Edmund Juszczak, Nimish V. Subhedar, Ursula Bowler, Charlotte Clarke, David Field, Elizabeth Hutchison, Wilf Kelsall, Justine Pepperell, Tracy Roberts, Sunil Sinha, Kayleigh Stanbury, Jonathan Wyllie, Pollyanna Hardy, Samantha Johnson

**Affiliations:** a Division of Neonatology, Sidra Medicine, Doha, Qatar; b Department of Engineering, Durham University, UK; c National Perinatal Epidemiology Unit Clinical Trials Unit, Nuffield Department of Population Health, University of Oxford, UK; d Nottingham Clinical Trials Unit, School of Medicine, University of Nottingham, Nottingham, UK; e Liverpool Women's NHS Foundation Trust, Liverpool, UK; f Nuffield Department of Population Health, University of Oxford, UK; g Department of Population Health Sciences, University of Leicester, UK; h NICU, Rosie Hospital, Cambridge University Hospital Foundation Trust, Cambridge, UK; i Institute of Applied Health Research, University of Birmingham, Birmingham, UK; j South Tees Hospitals NHS Foundation Trust, James Cook University Hospital, Middlesbrough, UK

**Keywords:** Patent ductus arteriosus, Premature, Neurodevelopmental outcomes, Respiratory outcome, Death

## Abstract

**Background:**

Children born extremely preterm are at increased risk of developmental problems and respiratory morbidity due to patent ductus arteriosus (PDA). The objective of this study was to evaluate whether early treatment of a PDA ≥1.5 mm with ibuprofen improved neurodevelopmental and respiratory outcomes at 24 months of age, corrected for prematurity.

**Methods:**

Baby-OSCAR was a UK multi-center placebo-controlled masked randomized clinical trial in infants born 23^+0^–28^+6^ weeks' gestation. The main long-term outcome was survival without moderate or severe neurodevelopmental impairment at 24 months’ corrected age, assessed using parent report primarily or classified by blinded end-point review committee where parent-reported data were not available. Other secondary outcomes included survival without respiratory morbidity and duration of oxygen supplementation. (ISRCTN Registry number ISRCTN84264977).

**Findings:**

From July 2015 through December 2020, 653 infants underwent randomization. At 24 months’ corrected age, outcome data were available for 537 children: 263 in the ibuprofen group and 274 in the placebo group. Survival without moderate to severe neurodevelopmental impairment in the ibuprofen and placebo groups was 131/248 (53.0%) and 134/259 (51.9%) respectively; adjusted risk ratio 1.01 (95% confidence interval [CI] 0.86–1.18); p = 0.901. Survival without respiratory morbidity was 66/220 (30%) and 74/225 (32.9%) respectively; adjusted risk ratio 0.89 (95% CI 0.68–1.18). Median duration of oxygen supplementation from randomization was 76.0 and 78.0 days, respectively; adjusted median difference −1.5 (−13.8 to 10.9).

**Interpretation:**

We found no evidence of an improvement in neurodevelopmental and respiratory outcomes at 24 months’ corrected age, after selective early treatment of a PDA ≥1.5 mm with ibuprofen in children born extremely preterm.

**Funding:**

This study was funded by the 10.13039/501100000272National Institute for Health Research (NIHR) Health Technology Assessment Programme (11/92/15).

**Research in context**

**Evidence before this study**

The neonatal interventions have short term effects on the outcomes to discharge from the hospital but more importantly can affect long term outcomes including neurodevelopment, respiratory morbidity and survival. The quality of life of survivors of intensive care reflects the usefulness of an intervention and thus the long-term outcomes are of particular interest to the family, health planners and clinicians. The concern surrounding long term outcomes have gained attention with the improvement in survival of infants of extremely short gestation who would previously have died. Improving long-term outcomes is now recognized as the most important primary objective of neonatal clinical trials, with the quality of life of the survivors of intensive care being the critical determinant of the usefulness of an intervention.

Management of patent ductus arteriosus (PDA) in extreme preterm infants has been debated for over 50 years. Various treatment approaches have been evaluated using different medications. The interventions reduce the prevalence of PDA but this does not translate into improvement in short term outcomes till discharge. On the contrary, the early targeted treatment of PDA with ibuprofen, a widely practiced approach over the last decade, has reported increased death and bronchopulmonary dysplasia till discharge. There is paucity of data from the clinical trials on long term neurodevelopmental outcomes after treatment of PDA in preterm infants.

The trial of prophylactic treatment of PDA with indomethacin in extremely-low-birth-weight infants, reported no improvement in the rate of survival without neurosensory impairment at 18 months, despite the fact that it reduced the frequency of patent ductus arteriosus and severe periventricular and intraventricular hemorrhage. (N Engl J Med 2001; 344:1966–72). Another smaller trial using early echocardiography-targeted ibuprofen treatment of a large PDA did not change the rate of survival without cerebral palsy at 2 year corrected age (J Pediatr 2021; 233:33–42). One observational study reported that treatment for PDA may be associated with a greater risk of adverse neurodevelopmental outcome at 2–3 years age (J Pediatr 2015 Nov; 167(5):1025–32). Another observational study reported an association between PDA surgical ligation and a nonoptimal neurodevelopmental outcome at 2 years of age for preterm infants born before 29 weeks of gestation (Neonatology 2016; 109(2):139–46).

There is no systematic review on long term neurodevelopmental outcomes after treatment of PDA due to very small number of randomised trials and the heterogeneity of intervention. Additionally, none of the studies have reported to date long term respiratory morbidity at 2 years or beyond after treatment of PDA in preterm infants during the neonatal period.

**Added value of this study**

This is the largest study in past 25 years to evaluate the treatment of PDA in extreme preterm infants and report on long term neurodevelopmental outcomes at 2 years corrected age. Major advances have taken place in last 2 decades on management of extreme preterm infants with significant improvement in survival of infants below 26 week gestational age. This study randomised over 300 infants that were below 26 weeks gestational age at birth, the largest cohort of interest to clinicians to date.

Additionally, this study reported the respiratory morbidity and survival at 2 years corrected age. The long term respiratory morbidity has never been reported to date after interventions for closure of PDA. This is even more important as the interventions to close the PDA using ibuprofen have reported significantly increased incidence of the short term outcome of bronchopulmonary dysplasia at 36 weeks corrected age.

The high follow up rate (over 80% at 2 years corrected age) and very low contamination with open treatment of PDA after randomization are very attractive and important consideration for interpretation of the results of this trial.

**Implications of all the available evidence**

There is increased risk of mortality and some complications of prematurity before discharge after closing the PDA. Additionally, there is no beneficial effect on survival without neuro-developmental improvement and respiratory morbidity after early PDA treatment. The results of this study along with other studies to date would allow the clinicians and families to make informed decision about the futility of intervention for closure of PDA. This could change the clinical practice and prompt clinicians to better select babies before exposing them to the interventions for closure of PDA.

## Introduction

Persistence of ductus arteriosus (PDA) beyond 72 h of age in extremely preterm born infants is associated with an increase in short-term neonatal morbidities including death, bronchopulmonary dysplasia (BPD) and intraventricular hemorrhage (IVH).^1^

The Baby-OSCAR trial, utilizing early targeted treatment of a large PDA (≥1.5 mm) with ibuprofen, reported no difference in death or bronchopulmonary dysplasia (BPD) at 36 weeks’ postmenstrual age, compared to placebo.^2^ There are limited data for core long-term outcomes such as death or neurodevelopmental impairment in babies receiving early targeted treatment with ibuprofen,^3^ and no data on long-term respiratory outcomes after interventions for closure of PDA.

Assessing long-term outcomes is essential for establishing the efficacy and safety of therapeutic interventions since they may have widespread effects beyond the neonatal period. These outcomes may be impacted as a direct consequence of an intervention, or indirectly via other causal mechanisms.^4^ An increase in BPD may impact later neurodevelopmental and respiratory function in children born extremely preterm.^5^^,^^6^

As part of the Baby-OSCAR trial, we followed up extremely preterm born infants at 24 months' corrected age to determine whether early targeted treatment of a PDA ≥1.5 mm improved survival without neurodevelopmental impairment. An assessment of survival without respiratory morbidity, and of duration of oxygen supplementation by 24 months’ corrected age was also undertaken.

## Methods

### Trial design

Baby-OSCAR was a multicenter, masked, randomized, placebo-controlled trial conducted in 32 tertiary neonatal intensive care units in the United Kingdom, coordinated by the National Perinatal Epidemiology Unit Clinical Trials Unit (NPEU CTU) at the University of Oxford. The authors confirm the accuracy and completeness of the data and for the fidelity of the trial to the published protocol.^7^ Ethics approval was received from the NHS Health Research Authority and East Midlands Nottingham 2 Research Ethics Committee (Ref: 14/EM/0172). The trial was funded by the National Institute for Health Research (NIHR) Health Technology Assessment programme.

### Participants

Infants eligible for participation comprised those born between 23 weeks + 0 days and 28 weeks + 6 days’ gestation, who were <72 h old, were confirmed by echocardiography to have a large PDA, defined as a PDA dimension more than or equal to 1.5 mm with a pulsatile flow. Full inclusion and exclusion criteria are provided in the Supplementary Appendix. Written informed consent was obtained from parents prior to randomization.

### Randomisation and masking

Randomisation details are provided in the Supplementary Appendix.

### Intervention and trial procedures

The trial intervention was one course of ibuprofen sodium, administered parenterally, as a loading dose of 10 mg per kilogram of body weight commenced within 72 h of birth, followed by two doses of 5 mg per kilogram at least 24 h apart. Placebo was administered as an equal volume of 0.9% sodium chloride.^7^

### Long-term outcomes

Mortality up to 24 months' corrected age was assessed by recording deaths before hospital discharge from each site. Deaths after discharge and up to 24 months' corrected age were recorded on the Baby-OSCAR administrative database, after checking directly with the recruiting hospital. Among survivors, long-term neurodevelopmental and respiratory outcomes were assessed at 24 months’ corrected age using validated parent report measures^8^ and a questionnaire for respiratory outcomes [see Supplementary Appendix]. The main long-term outcome was a composite of survival without moderate or severe neurodevelopmental impairment.

Commensurate with guidelines for classifying outcomes at age two years,^9^ neurodevelopmental impairment was defined as the presence of any one or more of.

o Moderate or severe cognitive impairment (Parent Report of Children's Abilities-Revised (PARCA-R) non-verbal cognitive scale standard score <70);^8^
o Moderate or severe language impairment (PARCA-R language scale standard score <70);
o Moderate or severe gross motor impairment (inability to walk or sit independently);
o Moderate or severe hearing impairment (hearing loss corrected or uncorrected by aids, or deaf); or
o Moderate or severe visual impairment (reduced vision uncorrected with aids, blindness in one eye with good vision in the contralateral eye, or blindness or light perception only).

For children for whom a parent questionnaire was not returned, was returned outside of the time window for deriving PARCA-R standard scores (<23.5 months or >27.5 months), or was returned but had substantial missing data, information from their two-year routine clinical follow-up assessment was obtained from medical notes, where available.

Respiratory outcomes comprised duration of oxygen supplementation from randomization to 24 months’ corrected age and a composite of survival without respiratory morbidity. Respiratory morbidity was defined as the presence of any two or more of the following:

o Need for oxygen or respiratory support
o Presence of a persistent cough and/or wheeze
o Need for regular treatment for respiratory morbidity
o ≥4 unscheduled attendances at hospital or a general practitioner for respiratory problems,
o ≥1 readmission to hospital for respiratory problems

Other long-term outcomes comprised the individual components of the main composite long-term outcome and the composite respiratory outcome. Foreseeable serious adverse events were reportable during the period between the first dose of ibuprofen or placebo and 7 days after the last dose, and are reported elsewhere.^2^

### Blinded end-point review

A blinded end-point review was conducted in accordance with a Blinded Endpoint Review Committee (BERC) Charter, written and agreed by the PMG and TSC. The BERC reviewed data from routine clinical follow-up assessments to identify the presence of neurodevelopmental impairment and classify the main long-term outcome. Data were anonymized and reviewed independently by BERC members (SJ, SG, NS) who were unaware of the randomization assignments. Outcomes were classified by consensus according to the pre-specified criteria for classifying neurodevelopmental impairment.

### Sample size

Assuming the risk of a child dying before 24 months' corrected age was 10%, we estimated that questionnaires would be sent out to parents of 660 surviving children. Assuming attrition of 20% reduced the effective sample size to 530. The proportion of infants expected to survive to 24 months’ corrected age without moderate or severe neurodevelopmental impairment in the placebo group was expected to be 55%.^10^ With outcome data available on a total sample size of around 600 (including deaths) the trial would have 80% power to detect an increase in survival without moderate or severe neurodevelopmental impairment of 11% (from 55 to 66%) and 90% power to detect an increase of 13% (from 55 to 68%).

### Statistical analysis

All infants with available long-term outcomes data were included in the analyses. Demographics and clinical characteristics at baseline and short-term outcomes are reported using descriptive statistics.

Analysis of PARCA-R data was based on age standardized scores. Multiple imputation by predictive mean matching was used to impute PARCA-R non-verbal cognitive and language scale standard scores for children for whom there was no classification from the BERC, and (i) the questionnaire was completed outside the time window for deriving standard scores (at <23.5 and >27.5 months of age, corrected for prematurity), and (ii) for PARCA-R non-verbal cognitive standardized scores if more than 4 questions were missing. Imputation models included minimization factors and any other variables associated with the outcome. Additional information on the multiple imputation model specification is given in the Supplementary Appendix. Binary outcomes were analyzed using mixed effects Poisson regression where possible adjusted for minimization factors as fixed effects, with center and multi-fetal pregnancies as random effects. Where convergence could not be achieved, Poisson regression was used adjusted for minimization factors with a robust variance estimator to account for the correlation between multifetal pregnancies. Unadjusted and adjusted risk ratios, adjusted risk differences and 95% confidence intervals are reported. Continuous outcomes were analyzed using mixed effects linear regression models with center and multifetal pregnancies as random effects; unadjusted and adjusted mean differences and 95% confidence intervals (after checking model assumptions) are reported. Where these assumptions were not met, quantile regression using the 50th centile was used, clustering on center and multifetal pregnancies; unadjusted and adjusted median differences and 95% confidence intervals are reported. The p-value for the main long-term outcome alone is reported. A sensitivity analysis was performed on the main long-term outcome, excluding infants with data multiply imputed.

Pre-specified subgroup analyses for the main long-term outcome were performed on the multiply imputed data sets. Pooled treatment effect estimates were obtained from the same regression models used for the primary analysis, including an interaction term between the subgroup and the study arm. Pooled adjusted risk ratios and 95% confidence intervals are reported.

Demographic and clinical characteristics collected at baseline and at 24 months, and important short-term outcomes, are described using descriptive statistics for children for whom long-term outcome data were and were not available, within each trial arm.

No formal method to adjust for multiplicity was used. The width of the confidence intervals has not been adjusted for multiplicity, and inferences drawn may not be reproducible and should not be used to infer definitive treatment effects. Full details of the statistical analysis are provided in the Statistical Analysis Plan [available from https://doi.org/10.1186/ISRCTN84264977]. Stata/SE, version 17 (StataCorp), was used for all analyses.

### Consent to participate

Parent(s) with legal parental responsibility of eligible babies were approached to discuss the trial further, answer any questions they may have had, and to request consent. Parent(s) had as much time as they needed to consider the information, and the opportunity to question the research team, or other independent parties to decide whether they wished to participate in the study. It was mandatory that informed consent for the study was obtained by a suitably qualified member of the study team. A parent had to personally sign and date the latest approved version of the Informed Consent form before any study specific procedures were performed. Parent(s) who could not speak English were only approached if an adult interpreter were available.

### Role of the funding source

The funder had no involvement in study design; in the collection, analysis, and interpretation of data; in the writing of the report; or in the decision to submit the paper for publication.

## Results

### Participants

Between July 2015 and December 2020, a total of 653 infants were randomized: 326 were assigned to receive ibuprofen and 327 were assigned to placebo. Follow-up for the assessment at 24-months' corrected age continued until 24-March-2023. Parental questionnaires were returned for 365 children, with an age range of 20–33 months’ corrected age (median 23, interquartile range 23–24).

**Fig. 1:**
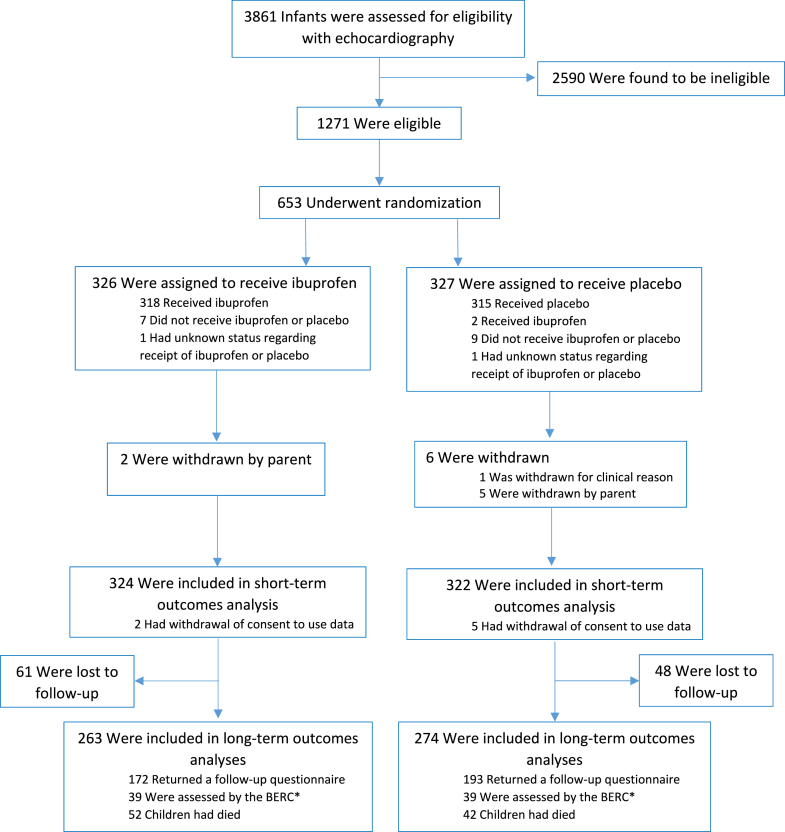
Participant flow chart. ∗ Blinded Endpoint Review Committee. An additional 4 (Ibuprofen) and 6 (Placebo) children with partially completed follow-up questionnaires were also classified by the BERC.

The analysis population for the long-term outcomes comprised 263 children (81%) assigned to receive ibuprofen and 274 children (84%) assigned to receive placebo (Fig. 1). This comprises children for whom a follow-up questionnaire was returned (172 and 193, ibuprofen and placebo groups, respectively), were classified by the BERC (39 and 39), or had died (52 and 42). For 30 children, data required to assess the main long-term outcome were not sufficient for classification based on the returned parental questionnaire or the BERC.

Maternal and infant baseline characteristics for children for whom long-term outcome data were available were well balanced between the groups (Table 1 and Table S1). The median diameter of the PDA at randomization was 2.2 mm (interquartile range, 1.9–2.6). Some imbalances were observed in short-term outcomes in children for whom long-term outcome data were available (Table 1). A higher proportion of children in the ibuprofen group died by or had moderate/severe BPD at 36 weeks’ post-menstrual age (69.9% vs. 62.6%), severe IVH (14.4% vs. 9.5%); cystic periventricular leukomalacia (5.3% vs. 2.9%) up to discharge home from hospital, or a closed or non-significant PDA (diameter <1.5 mm) at around 3 weeks of age (52.9% vs. 34.7%), compared with those in the placebo group. Known risk factors for respiratory morbidity reported at the 24-month follow-up were well balanced between the groups (Table S2).

**Table 1:** Baseline characteristics and short term outcomes for children for whom follow-up was assessed.

Characteristic/short-term outcome	Ibuprofen (n = 263)	Placebo (n = 274)
**Maternal characteristics**		
**Mother's ethnicity, no. (%)**		
White	178 (72.9)	190 (73.4)
Asian	34 (13.9)	41 (15.8)
Black	22 (9.0)	21 (8.1)
Other	10 (4.1)	7 (2.7)
*Missing, n*	*19*	*15*
**Mother's age (years)**		
Mean (SD)	30.9 (6.4)	30.7 (5.9)
**Deprivation index**^a^**, no. (%)**		
1 (least deprived)	79 (35.9)	84 (34.6)
2	50 (22.7)	61 (25.1)
3	41 (18.6)	38 (15.6)
4	30 (13.6)	37 (15.2)
5 (most deprived)	20 (9.1)	23 (9.5)
*Missing or not defined, n*	*43*	*31*
**Infant characteristics at randomization**		
**Postnatal age (hours)**^i^**, n (%)**		
Median [IQR]	57.3 [42.7–65.7]	56.1 [42.8–66.1]
<12 h	1 (0.4)	2 (0.7)
12–< 24 h	11 (4.2)	12 (4.4)
24–< 48 h	77 (29.3)	80 (29.2)
48–< 72 h	174 (66.2)	180 (65.7)
**Gestational age at birth (weeks)**^i^**, n (%)**		
Mean (SD)	26.0 (1.5)	26.1 (1.6)
**Mode of birth, n (%)**		
Vaginal birth—cephalic	108 (41.1)	117 (42.7)
Vaginal birth—breech	43 (16.3)	37 (13.5)
Caesarean section before onset of labour	70 (26.6)	71 (25.9)
Caesarean section after onset of labour	42 (16.0)	49 (17.9)
**Birth weight (g)**		
Mean (SD)	836.1 (207.7)	850.8 (211.8)
**Sex**^i^**, n (%)**		
Male	145 (55.1)	147 (53.6)
**APGAR score 5 min after birth, n (%)**		
Median [IQR]	8 [6–9]	7 [6–9]
*Missing, n*	*34*	*29*
**Size of Patent Ductus Arteriosus (PDA)**^i^**, n (%)**		
Median [IQR]	2.2 [1.9 to 2.5]	2.1 [1.9 to 2.6]
≥1.5 mm and <2.0 mm	70 (26.6)	70 (25.5)
≥2.0 mm and <3.0 mm	160 (60.8)	170 (62.0)
≥3.0 mm	33 (12.5)	34 (12.4)
**Mode of respiratory support at randomization**^i^**, n (%)**		
Invasive ventilation (by endotracheal tube)	166 (63.1)	175 (63.9)
Non-invasive respiratory support only^b^	95 (36.1)	97 (35.4)
Receiving no mechanical ventilation or pressure support^c^	2 (0.8)	0 (0.7)
**Receiving inotropes**^i^**, n (%)**	38 (14.4)	26 (9.5)
**Short-term outcomes up to discharge or 36 weeks' postmenstrual age**		
**Death by or moderate or severe bronchopulmonary dysplasia (BPD) at 36 weeks' postmenstrual age**^d^**, n (%)**	181 (69.9)	169 (62.6)
Infants survived up to 36 weeks' postmenstrual age, n	215	237
Missing	4	4
**Moderate or severe BPD at 36 weeks' postmenstrual age, n (%)**	137 (63.7)	136 (57.4)
**Severe intraventricular hemorrhage (grade III/IV**^e^**), n (%)**	38 (14.4)	26 (9.5)
**Cystic periventricular leukomalacia, n (%)**	14 (5.3)	8 (2.9)
**Treated for retinopathy of prematurity**^f^**, n (%)**	38 (14.4)	37 (13.5)
**NEC Bell stage II and above**^g^**, n (%)**	39 (14.9)	40 (14.6)
Missing, n	1	0
**Closed or non-significant PDA (< 1.5 mm) at around 3 weeks of age, confirmed by ECHO**^h^**, n (%)**	136 (52.9)	93 (34.7)
Missing, n	6	6
**Discharged home on oxygen, n (%)**	99 (38.2)	103 (38.3)
Missing, n	4	5
**Postnatal steroid use for chronic lung disease, n (%)**	64 (24.4)	67 (24.5)
Missing, n	1	0

SD denotes standard deviation and IQR interquartile range.

a Combines information from seven domains to produce an overall relative measure of deprivation. The domains are income; employment; education; skills and training; health and disability; crime; barriers to housing and services; and living environment.

b Nasal continuous positive airway pressure, nasal ventilation, humidified high flow nasal cannula therapy, or low flow oxygen ≥1.1 L/min.

c In room air, low flow oxygen <1.1 L/min, or ambient oxygen.

d Short-term primary outcome.

e With ventricular dilation or intraparenchymal abnormality.

f In at least one eye.

g Confirmed by radiography and/or histopathology.

h Deviation from the Statistical Analysis Plan due to incorrect short-term outcome specified.

i Denotes factor used in the randomization minimization algorithm.

### Outcomes

The main long-term outcome of survival without moderate or severe neurodevelopmental impairment at 24 months’ corrected age occurred in 131 of 248 children (53.0%) assigned to ibuprofen compared to 134 of 259 children (51.9%) assigned to placebo (after incorporating the multiply imputed standardized scores) adjusted risk ratio [aRR] 1.01 (95% CI, 0.86–1.18; p = 0.901) (Table 2). The sensitivity analysis excluding children whose PARCA-R scores were imputed showed a similar result (Table 2). Death occurred in 52 of 263 children (19.8%) assigned to ibuprofen and in 42 of 274 (15.3%) assigned to placebo (aRR, 1.23; 95% CI, 0.92 to 1.64). Among those children who survived, moderate or severe neurodevelopmental impairment was present in 57 of 174 children (32.8%) assigned to ibuprofen and 76 of 198 (38.4%) assigned to placebo (aRR, 0.85; 95% CI, 0.63–1.15; Table 2 and Table S5). Survival without respiratory morbidity occurred in 66 of 220 children (30.0%) in the ibuprofen group and 74 of 225 (32.9%) in the placebo group (aRR 0.89, 95% CI 0.68–1.18) (Table 3 and Table S6). Among those children who survived, respiratory morbidity was present in 102 of 168 (60.7%) in the ibuprofen group and 109 of 183 (59.6%) in the placebo group (aRR 1.05, 95% CI 0.89–1.23) (Table 3 and Table S6). The median duration of oxygen supplementation from randomization was 76 days (interquartile range [IQR] 38–166 days) in the ibuprofen group and 78 days (IQR 46–156 days) in the placebo group (adjusted median difference −1.5 days; 95% CI, −13.8 to 10.9) (Table 3 and Table S6).

**Table 2:** Main long-term outcome and its components.

	Ibuprofen (n = 263)	Placebo (n = 274)	Unadjusted risk ratio (95% CI)	Adjusted risk ratio (95% CI)	p-value
**Main long-term outcome—primary analysis**					
**Survival without moderate or severe neurodevelopmental impairment, n/N (%)**^c^	131/248 (53.0)	134/259 (51.9)	1.02^b^ (0.80–1.30)	1.01^a^^,^^b^ (0.86–1.18)	0.901
Missing, n	15	15			
**Main long-term outcome—sensitivity analysis excluding children for whom PARCA-R scores were multiply imputed**^d^					
**Survival without moderate or severe neurodevelopmental impairment, n/N (%)**	117/226 (51.8)	122/240 (50.8)	1.02 (0.79–1.31)	1.02^a^ (0.86–1.22)	0.790
Excluded from analysis,^e^ n	22	19			
Missing, n	15	15			
**Components of the main long-term outcome**					
**Death, n/N (%)**	52/263 (19.8)	42/274 (15.3)	1.29 (0.86–1.94)	1.23^d^ (0.92–1.64)	
**Children survived**	**n** = **211**	**n** = **232**			

**Moderate or severe neurodevelopmental impairment in survivors, n/N (%)**	57/174 (32.8)	76/198 (38.4)	0.85 (0.60–1.20)	0.85 (0.63–1.15)	
Not known^f^, n	37	34			
Moderate or severe non-verbal cognitive impairment, n (%)	38/171 (22.2)	50/201 (24.9)			
Not known^f^, n	40	31			
Moderate or severe language development impairment, n (%)	30/157 (19.1)	41/179 (22.9)			
Not known^f^, n	54	53			
Moderate or severe gross motor impairment	23/209 (11.0)	20/231 (8.7)			
Not known^f^, n	2	1			
Moderate or severe hearing impairment, n (%)	4/207 (1.9)	4/231 (1.7)			
Not known^f^, n	4	1			
Moderate or severe visual impairment, n (%)	8/206 (3.9)	7/230 (3.0)			
Not known^f^, n	5	2			

a Poisson model adjusted for size of PDA, gestational age at birth, age at randomization, sex, mode or respiratory support at randomization, whether the infant received inotropes at the time of randomization, multiple births, and center, with a robust variance estimator to account for the correlation between multifetal pregnancies.

b Analysed using multiply imputed data for 22 and 19 children in the ibuprofen and placebo groups respectively if the primary outcome was not already met and there was no classification from the Blinded Endpoint Review Committee for (i) PARCA-R non-verbal cognitive and language standardized scores for children for whom the questionnaire was completed outside the age range for deriving standardized scores (at <23.5 and >27.5 months of age, corrected for prematurity); and/or (ii) PARCA-R non-verbal cognitive standardized scores if more than 4 questions were missing.

c n/N (%) presented includes multiply imputed data.

d Mixed effects Poisson model adjusted for size of PDA, gestational age at birth, age at randomisation, sex, mode or respiratory support at randomisation, whether the infant received inotropes at the time of randomisation, multiple births, and center. Center and multiple births treated as random effects.

e Excluding children for whom PARCA-R non-verbal cognitive or language development scores were multiply imputed.

f Comprises follow-up questionnaire completed outside the age range for standardization for PARCA-R, or completed but with insufficient data, and with no classification by the Blinded Endpoint Review Committee.

**Table 3:** Respiratory morbidity outcomes.

	Ibuprofen (n = 263)	Placebo (n = 274)	Unadjusted risk ratio (95% CI)	Adjusted risk ratio (95% CI)
**Survival without respiratory morbidity**^a^**, n/N (%)**	66/220 (30.0)	74/225 (32.9)	0.91 (0.65–1.27)	0.89^b^ (0.68–1.18)
Missing, n	43	49		
**Duration of oxygen supplementation from randomization**^c^**, median [IQR]**	76.0 [38.0–166.0]	78.0 [46.0–156.0]	−2.0^d^ (−17.6 to 13.6)	−1.5^d^^,^^e^ (−13.8 to 10.9)
Missing^f^, n	20	24		
**Children survived**	**n** = **211**	**n** = **232**		

**Respiratory morbidity in survivors**^a^**, n/N (%)**	102/168 (60.7)	109/183 (59.6)	1.02 (0.78–1.33)	1.05^b^ (0.89–1.23)
Missing, n	43	49		
**Need for oxygen or respiratory support**^g^**, n/N (%)**	82/163 (50.3)	93/180 (51.7)		
Missing, n	48	52		
**Presence of a persistent cough and/or wheeze, n/N (%)**	**46/172 (26.7)**	**51/193 (26.4)**		
Missing, n	39	39		
**Need for regular treatment for respiratory illness, n/N (%)**	**94/172 (54.7)**	**97/193 (50.3)**		
Missing, n	39	39		
**4 or more unscheduled attendances at hospital/GP for respiratory problems, n/N (%)**	**39/172 (22.7)**	**56/191 (29.3)**		
Missing, n	39	41		
**Re-admission to hospital for respiratory problems, n/N (%)**	**76/155 (49.0)**	**94/179 (52.5)**		
Missing, n	56	53		

a Respiratory morbidity is defined as any 2 or more of: The need for oxygen or respiratory support; Presence of a persistent cough and/or wheeze; Need for regular treatment for respiratory illness; 4 or more unscheduled attendances at hospital/GP for respiratory problems; 1 or more readmission to hospital for respiratory problems.

b Mixed effects Poisson model adjusted for size of PDA, gestational age at birth, age at randomization, sex, mode or respiratory support at randomization, whether the infant received inotropes at the time of randomization, multiple births, and center. Center and multiple births treated as random effects.

c Duration of oxygen supplementation from randomization has been upper bounded so that the number of days on oxygen cannot exceed the number of days between date of randomization and date 2-years corrected age is reached.

d Value is the median difference (95% confidence interval).

e Bootstrapped quantile regression model adjusted for size of PDA, gestational age at birth, age at randomization, sex, mode or respiratory support at randomization, whether the infant received inotropes at the time of randomization, multiple births, and center. Clustered on center and multiple birth identifier.

f Missing if discharged home on oxygen and no data available for follow-up oxygen continuation or stopping date.

g Parent reported: “Since first discharge from hospital, has your child received any other breathing support?”. Multiple selections are possible.

**Fig. 2:**
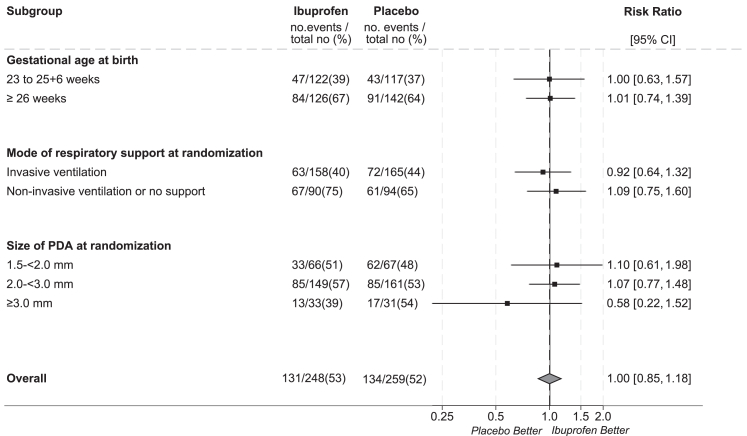
Subgroup analyses of main long-term outcome–Survival without moderate or severe neurodevelopmental impairment. Note: Analysed using multiply imputed data for 22 and 19 children in the ibuprofen and placebo groups respectively. Risk ratios and 95% confidence intervals were obtained from an interaction term between treatment assignment and subgroup characteristic of interest in a Poisson model adjusted for size of PDA, gestational age at birth, age at randomization, sex, mode or respiratory support at randomization, whether the infant received inotropes at the time of randomization, multiple births, and center, with a robust variance estimator to account for the correlation between multifetal pregnancies. No adjustments were made for multiplicity of testing, and therefore interpretation of the confidence intervals should not be used to assess treatment effect.

Prespecified subgroup analyses for the main long-term outcome are shown in Fig. 2. There was no evidence of a differential treatment effect for any of the subgroups.

### Characteristics of responders to follow-up

For children for whom long-term follow-up data were and were not available, maternal and infant baseline characteristics and short-term outcomes are described (Tables S3 and S4). Mothers of children with available outcome data (including deaths) were slightly older, and more likely to be non-white compared with those who did not have long-term outcome data available, with similar patterns across the trial groups (Table S3). More mothers of children with available long-term data assigned to ibuprofen had received any antenatal steroids and more children were from a multiple pregnancy and had received inotropes, with a reverse pattern seen in those assigned to placebo (Table S3). For short-term outcomes, fewer children with long-term outcome data available had moderate or severe BPD at 36 weeks’ postmenstrual age, had a closed or non-significant PDA (diameter <1.5 mm) at around 3 weeks of age, were discharged home on oxygen, or had received postnatal steroids up to discharge home compared with those who did not have long-term outcome data available, with a similar direction of results across trial groups (Table S4).

## Discussion

In this trial, there was no evidence of a difference in survival without moderate or severe neurodevelopmental impairment at 24 months’ corrected age, in extremely preterm born infants who were randomized to receive selective early treatment for a PDA ≥1.5 mm with ibuprofen compared with controls. There were also no significant between-group differences in the individual components of the main long-term outcome, death, survival without respiratory morbidity and duration of oxygen supplementation.

Overall, approximately 18% of infants died and 36% of survivors were classified with moderate or severe neurodevelopmental impairment, findings that are broadly comparable to other trials investigating long-term outcome after early pharmacological therapy of PDA.^3^^,^^11^^,^^12^ The TRIOCAPI randomized controlled trial of early ibuprofen therapy for a large PDA also found no difference in neurodevelopmental outcomes at 24 months’ corrected age in babies born <28 weeks of gestation, in which survival without cerebral palsy, assessed by formal neurological examination, occurred in 71.3% of ibuprofen-treated infants and 71.6% of controls.^3^ There were also no significant differences in any developmental domains assessed using the Ages and Stages Questionnaires (ASQ). The TIPP trial of prophylactic indomethacin reported death or severe neurosensory impairment (defined as cerebral palsy, cognitive delay, deafness or blindness at 18 months corrected age, as assessed by neurological examination and the Bayley Scales of Infant Development II) in 46% of very low birthweight infants treated with indomethacin vs. 47% of controls.^11^ The DETECT trial similarly reported no significant differences in long-term neurodevelopmental outcomes in preterm infants treated early for PDA with indomethacin.^13^

This report builds on our previously published short-term clinical outcomes which showed no effect of early selective ibuprofen therapy on death or moderate/severe BPD at 36 weeks' post-menstrual age (PMA).^2^ Death by 36 weeks' PMA occurred more frequently in treated infants and this tendency towards increased mortality persisted until 24 months’ corrected age, although neither were statistically significant at the 5% level. Whereas radiological markers of brain injury in the neonatal period (severe IVH and PVL) were also more commonly seen in ibuprofen-treated infants, this finding did not translate into an excess of long-term neurodevelopmental sequelae. One potential explanation for a lack of evidence of benefit of early ibuprofen therapy on long-term outcome includes the failure to achieve PDA closure in 47% of treated infants who were followed up. Whether earlier effective ductal closure would have resulted in improved long-term outcomes remains unknown.

Our results also show no evidence of a difference between groups in survival without respiratory morbidity at 24 months' corrected age, consistent with the earlier finding that early selective treatment of PDA with ibuprofen shows no evidence of an impact on rates of death or moderate/severe BPD in survivors at 36 weeks’ gestation.^2^ There was no evidence of a difference in respiratory morbidity amongst survivors and in duration of oxygen supplementation from randomization between the study groups. Extreme preterm birth is known to be associated with an increased risk of long-term respiratory morbidity,^14^ and this study provides novel data on long-term respiratory outcomes in infants treated with ibuprofen for PDA.

This is the largest follow-up of a clinical trial cohort reporting long-term data on survival and neurodevelopmental outcomes in extremely preterm infants following early ibuprofen therapy for PDA and the first to assess long-term respiratory outcomes.

Outcome assessments were performed using a validated parent-completed instrument. Cognitive and language impairment were assessed using the PARCA-R [www.parca-r.info], a parent-completed questionnaire for assessing cognitive and language development at 2 years of age. Unlike other developmental screening questionnaires, the PARCA-R has normative data from which age-standardized scores can be derived with a normative mean of 100 (SD 15), in keeping with other developmental tests, allowing classifications of impairment to be made using conventional definitions. Overall, parent questionnaires were returned for 65% of children alive at 2 years’ corrected age. Data for the analysis of the main long-term outcome were available for 82% of children who underwent randomization, including multiply imputed outcomes and blinded endpoint review of routine clinical data. Follow-up rates may be higher in neonatal trials in which outcomes are primarily assessed using clinical tests, which are preferable for assessing neurodevelopmental outcomes where feasible.

Among children for whom long-term data were available, maternal and infant baseline characteristics and short-term outcomes were generally well balanced between the trial arms suggesting adequate sample representation. A higher proportion of children in the ibuprofen group had moderate/severe BPD, IVH or PVL, but this did not translate into a higher rate of adverse neurodevelopmental or respiratory outcomes at 24 months.

An analysis of the characteristics of responders and non-responders showed mothers who responded to follow-up were slightly older and more likely to be non-white than those who did not respond to follow-up, and this was similar across trial arms. The higher proportion of non-white mothers among responders vs. non-responders is at odds with patterns of dropout typically observed in longitudinal studies, however responders in this trial included outcomes for babies who had died thus increasing the proportion who were likely to be non-white relative to other studies in which the denominator includes survivors only. The differences in maternal and infant baseline characteristics and short-term outcomes between responders and non-responders followed largely similar patterns across the trial groups or the numbers were too small to assess meaningfully, thus we do not expect this will have materially affected the results of the trial, which are consistent with other studies. Additionally, the closure rate of PDA in intervention arm was just over 50% and the placebo arm received open treatment with ibuprofen in about 30% babies. This reduces the perceived difference between the persistent PDA and thus the effects on long term health outcomes.

Whilst this trial is unique in its assessment of long-term neurodevelopmental and respiratory outcomes following early selective treatment for a PDA ≥1.5 mm, assessments at 2 years may have limited predictive validity for later outcomes. However, as there was no evidence that selective early treatment with ibuprofen improved either short or long-term outcomes in this trial, it is unlikely that there would be detectable effects on longer-term outcomes later in childhood.

In conclusion, among extremely preterm infants with a PDA ≥1.5 mm, we found no evidence that selective early treatment with ibuprofen was associated with improvement in survival without moderate or severe neurodevelopmental impairment at two years of age.

## Contributors

SG, PH & EJ were involved in the design of the Baby-OSCAR clinical trial, as well as in the conceptualisation, data curation, formal analysis, investigation, methodology, project administration, validation, funding acquisition, and writing and review of the manuscript; NS and SJ were involved in the conceptualisation, investigation, methodology, funding acquisition, conduct, and writing and review of the manuscript; TR was involved in the conceptualisation, data curation, formal analysis, investigation, methodology, validation, funding acquisition, and review of the manuscript; HOC was involved in data curation, formal analysis, investigation, methodology, project administration, validation, and writing and review of the manuscript; CC was involved in data curation, formal analysis, investigation, methodology, validation, and review of the manuscript; KS was involved in the conceptualisation, data curation, investigation, methodology, project administration, funding acquisition, and writing and review of the manuscript; UB was involved in the conceptualisation, data curation, investigation, methodology, project administration, funding acquisition, and review of the manuscript; DF, EH, WK, JP, SS and JW were involved in the conceptualisation, investigation, methodology, funding acquisition, and review of the manuscript. PH and HOC have directly accessed and verified the underlying data reported in the manuscript. All authors had full access to all the data in the study and had final responsibility for the decision to submit for publication.

## Data sharing statement

All data requests should be submitted to the corresponding author for consideration. Access to de-identified patient data may be granted for secondary analysis following review. If approved, a data sharing agreement will be put in place. Data availability will begin with publication.

## Declaration of interests

Samir Gupta received support from the National Institute for Health Research (NIHR) Health Technology Assessment (HTA) Programme (funding for the Baby-OSCAR study; payments made to institution. Grant Ref: 11/92/15) and has participated as: Member, DSMB, DENSe trial, India; Chair, DMC, TOAST trial (NIHR, UK); Member, DMC, EMBRACE trial, UK; Chair, Trial Steering committee NIHR, UK 152188.

Heather O'Connor received support from the National Institute for Health Research (NIHR) Health Technology Assessment (HTA) Programme (funding for the Baby-OSCAR study; payments made to institution. Grant Ref: 11/92/15).

Ed Juszczak received support from the NIHR (Payments were made to my institution).

Nimish Subhedar received research grants for neonatal pulmonary hypertension registry from Mallinckrodt Pharmaceuticals; Beyond Air and sponsorship of educational symposium Sept 2023 from Malinckrodt Pharmaceuticals.

Pollyanna Hardy received project funding from NIHR HTA (made to institution) and has participated as a committee member on NIHR HTA Commissioning Board.

Samantha Johnson received support from the National Institute for Health Research (NIHR) Health Technology Assessment (HTA) Programme (funding for the Baby-OSCAR study; payments made to institution. Grant Ref: 11/92/15).

Ursula Bowler, Charlotte Clarke, David Field, Elizabeth Hutchison, Wilf Kelsall, Justine Pepperell, Tracy Roberts, Sunil Sinha, Kayleigh Stanbury and Jonathan Wyllie declare no competing interests.

## Appendix A Supplementary data

Supplementary Appendix

Protocol

Charter for BERC

## References

[1] Sellmer A., Bjerre J.V., Schmidt M.R. (2013). Morbidity and mortality in preterm neonates with patent ductus arteriosus on day 3. Arch Dis Child Fetal Neonatal Ed.

[2] Gupta S., Subhedar N.V., Bell J.L. (2024). Trial of selective early treatment of patent ductus arteriosus with ibuprofen. N Engl J Med.

[3] Rozé J.C., Cambonie G., Le Thuaut A. (2021). Effect of early targeted treatment of ductus arteriosus with ibuprofen on survival without cerebral palsy at 2 years in infants with extreme prematurity: a randomized clinical trial. J Pediatr.

[4] Marlow N., Doyle L.W., Anderson P. (2019). Assessment of long-term neurodevelopmental outcome following trials of medicinal products in newborn infants. Pediatr Res.

[5] Twilhaar E.S., Wade R.M., de Kieviet J.F., van Goudoever J.B., van Elburg R.M., Oosterlaan J. (2018). Cognitive outcomes of children born extremely or very preterm since the 1990s and associated risk factors: a meta-analysis and meta-regression. JAMA Pediatr.

[6] Fawke J., Lum S., Kirkby J. (2010). Lung function and respiratory symptoms at 11 years in children born extremely preterm: the EPICure study. Am J Respir Crit Care Med.

[7] Gupta S., Juszczak E., Hardy P. (2021). Study protocol: baby-OSCAR trial: outcome after selective early treatment for closure of patent ductus ARteriosus in preterm babies, a multicentre, masked, randomised placebo-controlled parallel group trial. BMC Pediatr.

[8] Johnson S., Bountziouka V., Brocklehurst P. (2019). Standardisation of the Parent Report of Children's Abilities-Revised (PARCA-R): a norm-referenced assessment of cognitive and language development at age 2 years. Lancet Child Adolesc Health.

[9] Marlow N., Abbott J., Field D., Johnson S., Huertas A., Jones H. (2008).

[10] Mangham L.J., Petrou S., Doyle L.W., Draper E.S., Marlow N. (2009). The cost of preterm birth throughout childhood in England and Wales. Pediatrics.

[11] Schmidt B., Davis P., Moddemann D. (2001). Long-term effects of indomethacin prophylaxis in extremely-low-birth-weight infants. N Engl J Med.

[12] Kluckow M., Jeffery M., Gill A., Evans N. (2014). A randomised placebo-controlled trial of early treatment of the patent ductus arteriosus. Arch Dis Child Fetal Neonatal Ed.

[13] Varghese J., Gill A., McMichael J. (2016). Follow up at 2 years of an early ductal targeted treatment (DETECT) trial. J Paediatr Child Health.

[14] Gutvirtz G., Wainstock T., Sheiner E., Pariente G. (2022). Prematurity and long-term respiratory morbidity-what is the critical gestational age threshold?. J Clin Med.

