# Corrigendum to ‘Two-year outcomes after selective early treatment of patent ductus arteriosus with ibuprofen in preterm babies: follow-up of Baby-OSCAR–a randomised controlled trial’ [eClinicalMedicine, Volume 87, September 2025, 103424]

**DOI:** 10.1016/j.eclinm.2025.103718

**Published:** 2025-12-29

**Authors:** Samir Gupta, Heather O'Connor, Edmund Juszczak, Nimish V. Subhedar, Ursula Bowler, Charlotte Clarke, David Field, Elizabeth Hutchison, Wilf Kelsall, Justine Pepperell, Tracy Roberts, Sunil Sinha, Kayleigh Stanbury, Jonathan Wyllie, Pollyanna Hardy, Samantha Johnson

**Affiliations:** a Division of Neonatology, Sidra Medicine, Doha, Qatar; b Department of Engineering, Durham University, UK; c National Perinatal Epidemiology Unit Clinical Trials Unit, Nuffield Department of Population Health, University of Oxford, UK; d Nottingham Clinical Trials Unit, School of Medicine, University of Nottingham, Nottingham, UK; e Liverpool Women's NHS Foundation Trust, Liverpool, UK; f Nuffield Department of Population Health, University of Oxford, UK; g Department of Population Health Sciences, University of Leicester, UK; h NICU, Rosie Hospital, Cambridge University Hospital Foundation Trust, Cambridge, UK; i Institute of Applied Health Research, University of Birmingham, Birmingham, UK; j South Tees Hospitals NHS Foundation Trust, James Cook University Hospital, Middlesbrough, UK

## Explanatory text

During a routine review of the publication, the authors identified some errors in the numbers presented. The errors relate to:

1) ‘Mode of respiratory support at randomization’ in Table 1.

**Table 1:** Baseline characteristics and short term outcomes for children for whom follow-up was assessed.

Characteristic/short-term outcome	Ibuprofen (n = 263)	Placebo (n = 274)
**Infant characteristics at randomization**		
**Mode of respiratory support at randomization**^i^**, n (%)**		
Invasive ventilation (by endotracheal tube)	166 (63.1)	175 (63.9)
Non-invasive respiratory support only^b^	95 (36.1)	97 (35.4)
Receiving no mechanical ventilation or pressure support^c^	2 (0.8)	2 (0.7)

b Nasal continuous positive airway pressure, nasal ventilation, humidified high flow nasal cannula therapy, or low flow oxygen ≥1.1 L/min.

c In room air, low flow oxygen <1.1 L/min, or ambient oxygen.

i Denotes factor used in the randomization minimization algorithm.
Numbers that require correction: Number receiving no mechanical ventilation or pressure support in the placebo group was presented as 0.
Explanation: This was a typographical error requiring a minor correction to the number receiving no mechanical ventilation or pressure support. The percentage was correct.
Correction made: Number receiving no mechanical ventilation or pressure support in the placebo group corrected to 2.
2) Results presented in the text for the median diameter of the PDA at randomization.
Numbers that require correction: The upper quartile was presented as 2.6.
Explanation: This was a typographical error requiring a minor correction to the upper quartile.
Correction made: The upper quartile for the median diameter of the PDA at randomization corrected to 2.5.
3) Results presented in the text and Table 3 for ‘Survival without respiratory morbidity’ and ‘Respiratory morbidity in survivors’.

**Table 3:** Respiratory morbidity outcomes.

	Ibuprofen (n = 263)	Placebo (n = 274)	Unadjusted risk ratio (95% CI)	Adjusted risk ratio (95% CI)
**Survival without respiratory morbidity**^a^**, n/N (%)**	66/210 (31.4)	74/220 (33.6)	0.93 (0.67–1.30)	0.92^b^ (0.70–1.20)
*Missing, n*	*53*	*54*		
**Duration of oxygen supplementation from randomization**^c^**, median [IQR]**	76.0 [38.0–166.0]	78.0 [46.0–156.0]	−2.0^d^ (−17.3 to 13.3)	−1.5^d^^,^^e^ (−13.8 to 10.9)
*Missing*^f^*, n*	*20*	*24*		
**Children survived**	**n** = **211**	**n** = **232**		
**Respiratory morbidity in survivors**^a^**, n/N (%)**	92/158 (58.2)	104/178 (58.4)	1.00 (0.75–1.32)	1.02^b^ (0.87–1.21)
*Missing, n*	*53*	*54*		
**Need for oxygen or respiratory support**^g^**, n/N (%)**	107/165 (64.8)	120/186 (64.5)		
*Missing, n*	*46*	*46*		
**Presence of a persistent cough and/or wheeze, n/N (%)**	**46/172 (26.7)**	**51/193 (26.4)**		
*Missing, n*	*39*	*39*		
**Need for regular treatment for respiratory illness, n/N (%)**	**94/172 (54.7)**	**97/193 (50.3)**		
*Missing, n*	*39*	*39*		
**4 or more unscheduled attendances at hospital/GP for respiratory problems, n/N (%)**	**39/172 (22.7)**	**56/191 (29.3)**		
*Missing, n*	*39*	*41*		
**Re-admission to hospital for respiratory problems, n/N (%)**	**76/155 (49.0)**	**94/179 (52.5)**		
*Missing, n*	*56*	*53*		

a Respiratory morbidity is defined as any 2 or more of: The need for oxygen or respiratory support; Presence of a persistent cough and/or wheeze; Need for regular treatment for respiratory illness; 4 or more unscheduled attendances at hospital/GP for respiratory problems; 1 or more readmission to hospital for respiratory problems.

b Mixed effects Poisson model adjusted for size of PDA, gestational age at birth, age at randomization, sex, mode or respiratory support at randomization, whether the infant received inotropes at the time of randomization, multiple births, and center. Center and multiple births treated as random effects.

c Duration of oxygen supplementation from randomization has been upper bounded so that the number of days on oxygen cannot exceed the number of days between date of randomization and date 2-years corrected age is reached.

d Value is the median difference (95% confidence interval).

e Bootstrapped quantile regression model adjusted for size of PDA, gestational age at birth, age at randomization, sex, mode or respiratory support at randomization, whether the infant received inotropes at the time of randomization, multiple births, and center. Clustered on center and multiple birth identifier.

f Missing if discharged home on oxygen and no data available for follow-up oxygen continuation or stopping date.

g Parent reported: “Since first discharge from hospital, has your child received any other breathing support?”. Multiple selections are possible.
Numbers that require correction: Survival without respiratory morbidity was presented as 66 of 220 children (30.0%) (and 43 with missing data) in the ibuprofen group and 74 of 225 (32.9%) (and 49 with missing data) in the placebo group (unadjusted Risk Ratio [RR] 0.91, 95% Confidence Interval [CI] 0.65–1.27), adjusted RR 0.89, 95% CI 0.68–1.18); respiratory morbidity in survivors was presented as 102 of 168 (60.7%) (and 43 with missing data) in the ibuprofen group and 109 of 183 (59.6%) (and 49 with missing data) in the placebo group (unadjusted RR 1.02, 95% CI 0.78–1.33), adjusted RR 1.05, 95% CI 0.89–1.23).
Explanation: A review of the derivation of this composite outcome, following peer review feedback, identified that infants with missing data for one of the components were inadvertently included. This has been corrected requiring minor corrections to the numerators, denominators, corresponding percentages, missing data, and the risk ratios and 95% CIs.
Corrections made:
For ‘Survival without respiratory morbidity’:-
  denominator, percentage and missing data for the ibuprofen group corrected to 210, 31.4% and 53 with missing data;
  denominator, percentage and missing data for the placebo group corrected to 220, 33.6% and 54 with missing data;
  unadjusted RR and 95% CI corrected to 0.93, 95% CI 0.67–1.30;
  adjusted RR and 95% CI corrected to 0.92, 95% CI 0.70–1.20.
For ‘respiratory morbidity in survivors’:-
  numerator, denominator, percentage and missing data for the ibuprofen group corrected to 92 (numerator), 158 (denominator), 58.2% and 53 with missing data;
  numerator, denominator, percentage and missing data for the placebo group corrected to 104 (numerator), 178 (denominator), 58.4% and 54 with missing data;
  unadjusted RR and 95% CI corrected to 1.00, 95% CI 0.75–1.32;
  adjusted RR and 95% CI corrected to 1.02, 95% CI 0.87–1.21.
4) Results presented in Table 3 for ‘Need for oxygen or respiratory support’.
Numbers that require correction: Need for oxygen or respiratory support was presented as 82/163 (50.3%) (and 48 with missing data) for the ibuprofen group, and 93/180 (51.7%) (and 52 with missing data) in the placebo group.
Explanation: A review of the derivation of this component of the respiratory morbidity outcome, following peer review feedback, identified that it was only including infants who received oxygen since discharge from hospital. This was corrected to also include infants who received other types of respiratory support following discharge. This did not have an impact on the numbers meeting the composite outcome. This has been corrected requiring minor corrections to the numerators, denominators and corresponding percentages.
Corrections made: For ‘Need for oxygen or respiratory support’:-
  numerator, denominator, percentage and missing data for the ibuprofen group corrected to 107 (numerator), 165 (denominator), 64.8% and 46 with missing data;
  numerator, denominator, percentage and missing data for the placebo group corrected to 120 (numerator), 186 (denominator), 64.5% and 46 with missing data.
5) The forest plot (Fig. 2) for the subgroup analysis by ‘Size of PDA at randomization’ and the overall result presented on the forest plot.

**Fig. 2:**
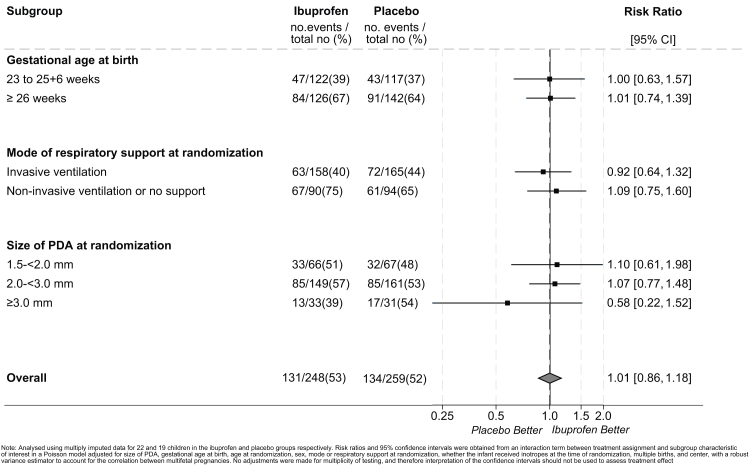
**Subgroup analyses of main long-term outcome–Survival without moderate or severe neurodevelopmental impairment**.
Numbers that require correction: The numerator for the size of PDA at randomization subgroup of 1.5–<2.00 mm in the placebo group was presented as 62, and the overall RR and 95% CI was presented as 1.00 [0.85, 1.18].
Explanation: These were typographical errors requiring a correction to the numerator for the size of PDA at randomization subgroup of 1.5–<2.00 mm in the placebo group, and a minor correction to the overall risk ratio and 95% CI.
Corrections made: Numerator for the size of PDA at randomization subgroup of 1.5–<2.00 mm in the placebo group corrected to 32; overall RR and 95% CI corrected to 1.01 [0.86, 1.18].
6) Text in the discussion relating to the percentage of babies receiving open-label treatment.
Numbers and text that require correction: The percentage and text in the sentence were incorrectly written as “… and the placebo arm received open treatment with ibuprofen in about 30% babies.”
Explanation: The percentage has been corrected to reflect the denominator for the 2-year outcome population (previously this was based on the denominator for the previously published short-term outcome population), and the wording has been corrected to better reflect the wording of the variable that this percentage is based on.
Corrections made: The percentage has been corrected to 25% and the wording corrected to “medical open-label treatment of a symptomatic PDA with a Cox inhibitor”.
7) Percentages presented for ‘Need for respiratory support’ in Table S6.

**Table S6:** Components of respiratory morbidity outcomes–additional detail.

	Ibuprofen (n = 263)	Placebo (n = 274)
**Duration of oxygen supplementation from randomisation (days)**^a^		
Mean (SD)	148.9 (191.7)	138.6 (167.9)
Median [IQR]	76.0 [38.0–166.0]	78.0 [46.0–156.0]
(Min to max)	(3–841)	(2–830)
Missing, n	20	24
**Children survived**	n = 211	n = 232
**Need for oxygen or respiratory support, n (%)**	107 (64.8)	120 (64.5)
Missing, n	46	46
Discharged home on oxygen	81 (77.1)	97 (72.5)
Missing, n	2	0
Still on oxygen	10 (9.5)	6 (5.0)
Missing, n	2	0
Received any oxygen at other time(s) since discharge	22 (59.5)	25 (61.0)
Missing, n	70	79
Need for respiratory support^b^, n (%)	31 (29.2)	36 (30.0)
Ventilator	5 (16.7)	15 (41.7)
CPAP	9 (30.0)	5 (13.9)
Tracheostomy	1 (3.3)	0 (0.0)
Other	15 (50.0)	16 (44.4)
Missing, n	1	0
**Presence of a persistent cough and/or wheeze, n (%)**	46 (26.7)	51 (26.4)
Missing, n	39	39
Persistent cough	28 (60.9)	28 (54.9)
Affects feeding, n (%)	12 (44.4)	12 (44.4)
Missing	1	1
Affects sleep, n (%)	23 (85.2)	21 (77.8)
Missing	1	1
Affects physical activity, n (%)	17 (60.7)	13 (50.0)
Missing	0	2
Persistent wheeze	40 (87.0)	45 (88.2)
**Need for regular treatment for respiratory illness, n (%)**	94 (54.7)	97 (50.3)
Missing, n	39	39
**Inhaler—reliever**	62 (70.5)	66 (70.2)
Missing, n	6	3
**Inhaler—preventer**	15 (19.7)	28 (30.8)
Missing, n	18	6
**Steroids**	31 (39.7)	33 (36.3)
Missing, n	16	6
**Other**	40 (50.0)	36 (39.1)
Missing, n	14	5
**Unscheduled attendances at hospital/GP for respiratory problems, n(%)**		
0 attendances	66 (38.4)	67 (35.1)
1–3 attendances	67 (39.0)	68 (35.6)
4–12 attendances	30 (17.4)	51 (26.7)
More than 12 attendances	9 (5.2)	5 (2.6)
Missing, n	39	41
**Re-hospitalisation for respiratory problems, n(%)**		
0 admissions	79 (51.0)	85 (47.5)
1–2 admissions	51 (32.9)	56 (31.3)
3–5 admissions	15 (9.7)	25 (14.0)
More than 5 admissions	10 (6.5)	13 (7.3)
Missing, n	56	53

a Duration of oxygen supplementation from randomisation has been upper bounded so that the number of days on oxygen cannot exceed the number of days between date of randomisation and date 2-years corrected age is reached.

b Parent reported: “Since first discharge from hospital, has your child received any other breathing support?”. Multiple selections are possible.
Numbers that require correction: Percentages were presented as 38.3% for the ibuprofen group, and 35.5% for the placebo group.
Explanation: The denominator for this variable was not updated following a review of the derivation of this outcome, requiring a minor correction to the percentages.
Corrections made: Percentages corrected to 29.2% for the ibuprofen group, and 30.0% for the placebo group.

## Corrections

Below are the correct versions of the relevant sections where these numbers are mentioned.

## Summary: findings

Survival without respiratory morbidity was 66/210 (31.4%) and 74/220 (33.6%) respectively; adjusted risk ratio 0.92 (95% CI 0.70–1.20).

## Results: participants

The median diameter of the PDA at randomization was 2.2 mm (interquartile range, 1.9–2.5).

## Results: outcomes

Survival without respiratory morbidity occurred in 66 of 210 children (31.4%) in the ibuprofen group and 74 of 220 (33.6%) in the placebo group (aRR 0.92, 95% CI 0.70–1.20) (Table 3 and Table S6). Among those children who survived, respiratory morbidity was present in 92 of 158 (58.2%) in the ibuprofen group and 104 of 178 (58.4%) in the placebo group (aRR 1.02, 95% CI 0.87–1.21) (Table 3 and Table S6).

## Discussion: penultimate sentence of paragraph 8

Additionally, the closure rate of PDA in intervention arm was just over 50% and the placebo arm received medical open-label treatment of a symptomatic PDA with a COX inhibitor in about 25% of babies.

## Appendix A Supplementary data

**Supplementary Appendix**

## Impact on conclusions

The authors confirm that the updated numbers were produced using the original, verified source data and has undergone rigorous quality control to ensure its accuracy. No other sections of the manuscript were affected by these errors.

The authors have thoroughly reviewed the corrected data and confirm that the overall conclusions of the study remain unchanged. The study findings are still valid and supported by the corrected numbers.

## Author's statement

We confirm that there are no additional errors in the data submitted to the journal. The accuracy of all other figures, tables, and findings has been verified, ensuring the integrity and reliability of the manuscript as a whole.

