# Supplementary material for: Two-year outcomes after selective early treatment of patent ductus arteriosus with ibuprofen in preterm babies: follow-up of Baby-OSCAR–a randomised controlled trial
Source: eClinicalMedicine. 2025 Aug 20;87:103424. doi: 10.1016/j.eclinm.2025.103424 (PMC12396396; doi:10.1016/j.eclinm.2025.103424)
Supplement: Supplementary Appendix [file mmc1.docx]

Supplementary Appendix

Gupta S, O’Connor H, Juszczak, J et al. Two-Year Outcomes After Selective Early Treatment of Patent Ductus Arteriosus with Ibuprofen in Preterm Babies (Follow-Up of Baby-OSCAR - A Randomised Controlled Trial) DOI: XXXX

This appendix has been provided by the authors to give readers additional information about their work.

Table of Contents

[Baby-OSCAR Collaborative Group 2](#_Toc201829928)

[Study Committees 3](#_Toc201829929)

[Acknowledgements 3](#_Toc201829930)

[Methods 4](#_Toc201829931)

[Inclusion & exclusion criteria 4](#_Toc201829932)

[Randomisation detail 4](#_Toc201829933)

[Two year follow up form 4](#_Toc201829934)

[Multiple imputation of missing primary outcome data 4](#_Toc201829935)

[Tables 5](#_Toc201829936)

[Table S1: Additional baseline characteristics for children for whom long-term outcomes were assessed 5](#_Toc201829937)

[Table S2: Risk factors for respiratory morbidity collected at 2-year follow-up 6](#_Toc201829938)

[Table S3: Baseline characteristics by availability of 2-year follow-up data 6](#_Toc201829939)

[Table S4: Short-term outcomes by availability of 2-year follow-up data 10](#_Toc201829940)

[Table S5: Components of main long-term outcome – additional detail 11](#_Toc201829941)

[Table S6: Components of respiratory morbidity outcomes – additional detail 12](#_Toc201829942)

# Baby-OSCAR Collaborative Group

The following investigators, research nurses, and hospitals participated in the Baby-OSCAR Study. Sites are listed alphabetically.

| **Recruiting site for Baby-OSCAR** | **PI(s)** | **Research Staff** |
| --- | --- | --- |
| Aberdeen Maternity Hospital | Dr Saulius Satas | Margaret Connon, Stephen Main, Susan MacFarlane |
| Addenbrookes’s Hospital, Cambridge | Dr Anthony Wilfred Ross Kelsall | Katherine Bradly Russell, Helen Shelley, Beth Berthlecon, Dr Sajeev Job |
| Arrowe Park Hospital, Wirral | Dr Anand Kamalanathan, | Sharon Hughes, Lucy Lewis, Dr Aung Soe |
| Birmingham Heartlands Hospital | Dr Jaideep Singh | Eve Irvine, Katie Price, Laura Thrasyvoulou, Juneka Begum, Jacqueline Daglish |
| Birmingham Women’s Hospital | Dr Vishna Rasiah  Dr Anju Singh | Rachel Jackson, Efygenia Kotsia, Amy Woodhead, Abby Twiss, Maxine Heather Barrow, Elizabeth Simcox |
| Bradford Royal Infirmary | Dr Sam Wallis | Rachel Wane |
| Burnley General Hospital | Dr Savithiri Sivashankar | Emily Andrews, Heather Collier, Dr Chi-Ning Gerrard, Caroline Cowman, Bev Hammond, Frances Pickering |
| Derriford Hospital ,Plymouth | Dr Robert John Madar, | Sarah-Jane Sharman, Alison Stolton |
| James Cook University Hospital, Middlesbrough | Dr Jonathan Wyllie | Caroline Buckley, Ms Amanda Forster, Helena Smith, Suzanne Bell, |
| Jessop Wing Hospital, Sheffield | Dr Porus Bustani | Pauline Bayliss, Rachel Sellars, Lynne Smart, Liz Taylor, Pauline Bayliss, Beth Lally |
| Leeds General Infirmary | Dr Lawrence Miall | Nicola Balatoni, Suzanne Laing, Collette Spencer, Sarah Thornton, Lindsay Uryn, Dr Laura Dalton, Dr Katherine Pettinger, Charlotte Reilly |
| Leicester Royal Infirmary | Dr Jonathan Cusack | Marie Hubbard, Rosalind Astles, Maria Sharpe, Jennifer Smith |
| Liverpool Women’s Hospital | Dr Nimish Subhedar | Karen Harvey, Joanne Windrow, Patrick McGowan, Amy Beasley |
| Luton & Dunstable University Hospital | Dr Sateeshkumar Somisetty | Yvonne Millar, Olaitan Adesiyan, Jenny Baker |
| Medway Maritime Hospital | Dr Santosh Pattnayak | Helen Harizaj, Ms Aimee Harris, Sarah Jones, Alison Youdale, Dr Aung Soe |
| Norfolk and Norwich University Hospital | Dr Rahul Roy | Samantha Claire, Dr Supriya Bhoomaiah, Karen Few, Katherine Lloyd, Amy Nichols, Laura Playne |
| Queen Charlotte’s and Chelsea Hospital | Dr Jay Banerjee | Batia Gourin, Zoe McClure, Kirupalini Mariampillai, Dr Sundar Satyamurthy |
| Royal Infirmary of Edinburgh | Dr Christopher Kissack | Sally Yip, Lynn Clark |
| Royal Jubilee Maternity Hospital, Belfast | Dr Bharathi Rao | Eileen Killen, Jennifer McGowan, Muriel Millar, Mary O'Neill, Angela Abbate, Rachel Anderson, Julie Brown, Patrick Lawlor, Judith Ratcliffe, Eileen Rogers |
| Royal Preston Hospital, Preston | Dr Akaolisa Egbeama | Joanna Lees, Claire Lodge, Natalie Morgan, Dr Raju Narasimhan, Paula Sugden |
| Royal Victoria Infirmary Newcastle | Dr Sundeep Harigopal | Julie Groombridge, Tracey Downes |
| St George’s Hospital, London | Dr Donovan Duffy | Naomi Hayward, Dr Anay Kulkarni |
| St Mary’s Hospital, London | Dr Jay Banerjee | Batia Gourin, Zoe McClure, Izabela Andrzejewska, Kirupalini Mariampillai, Vania Oliveira, Dr Sundar Sathiyamurthy |
| St Mary’s Hospital, Manchester | Dr Arindam Mukherjee | Nicola Booth, Karen Dockery, Clare Jennings, Louise Weaver-Lowe, Katherine Birchall |
| Sunderland Royal Hospital, | Dr Majid Abu-Harb | Natalie Talbot, Paul Corrigan |
| The Grange University Hospital | Dr Siddhartha Sen | Alison Davies, Angela Harris |
| The Royal London Hospital | Dr Ajay Sinha | MaySze Chang, Caroline Francia, Ivone Lancoma-Malcolm, Gail Falder, Dr Rainer Ebel |
| University Hospital Coventry | Dr Mrinalini Rajimwale | Francesca Brewer, Rebecca Grenfell, Nicola Watts, Laura Wild, Nicolas Aldridge, Susan Dale, Jo Gmerek, Kerri McGowan. |
| University Hospital of North Tees | Dr Samir Gupta  Dr Sundaram Janakiraman | Alex Ramshaw, Wendy Cheadle, Dr Harikumar |
| Watford General Hospital | Dr Nazakat Merchant | Shabana Malik, Suminthra Naidu, Rona Verdadero |
| William Harvey Hospital, Ashford | Dr Amit Gupta | Shermi George, Claire Moloney, Vimal Vasu. |
| Wishaw General Hospital | Dr Gopala Krishnan | Denise Vigni |

### National Perinatal Epidemiology Unit Clinical Trials Unit, University of Oxford, UK

Jennifer Bell, Ursula Bowler, Charlotte Clarke, Christina Cole, Kerrianne Dempster, Clare Edwards, Pollyanna Hardy, Nina Jamieson, Edmund Juszczak, Ann Kennedy, Andy King, Marketa Laube, Louise Linsell, David Murray, Heather O’Connor, Rema Ramakrishnan, Charles Roehr, Kayleigh Stanbury, Julia Sutton, Richard Welsh, Joy Wiles.

### Research & Development Department, University Hospital of North Tees, Stockton, UK

Jane Greenaway, Pauline Shephard, Dr Volker Straub, Dr Justin Carter

# Study Committees

### Independent Trial Steering Committee Members

Ben Snook

Dr Denis Azzopardi

Emeritus Professor Michael Weindling (Chair)

Dr Narender Aladangady

Sophie Welch

Dr Tim Clayton

### Independent Data Monitoring Committee Members

Professor Alan Montgomery

Professor David Edwards (Chair)

Dr Heike Rabe

# Acknowledgements

We thank all the families, infants, and hospital staff at recruiting and continuing care sites who participated in the trial.

We acknowledge independent advice from Dr Nicholas Evans and Dr Steven M. Donn in planning the trial. We’d also like to thank Rema Ramakrishnan for her support with the validation of the analysis.

# Methods

## Inclusion & exclusion criteria

### Inclusion Criteria

Babies will be considered eligible for inclusion into the trial if they are:

- Born at 23^+0^ to 28^+6^ weeks of gestation
- Less than 72 hours old
- Confirmed by echocardiography as having a large PDA which
  - is at least 1.5 mm in diameter (determined by gain optimised colour Doppler)

**and**

- - has unrestrictive pulsatile left to right flow (ratio of flow velocity in PDA Maximum (Vmax) to Minimum (Vmin) > 2:1)

In addition:

- The responsible clinician is uncertain about whether the baby might benefit from treatment to close the PDA
- Written informed consent has been obtained from the parent(s)

### Exclusion Criteria

Babies will be excluded from participation in the trial if they have:

- No realistic prospect of survival
- Severe congenital anomaly
- Clinical or echocardiography suspicion of congenital structural heart disease that contraindicates treatment with ibuprofen
- Other conditions that would contraindicate the use of ibuprofen (clinically significantly intracranial or gastrointestinal haemorrhage, coagulopathy, thrombocytopenia (platelet count <50,000), renal failure, pulmonary hypertension, known or suspected necrotising enterocolitis (NEC))
- Indomethacin, ibuprofen, or paracetamol administration after birth

## Randomisation detail

Dynamic allocation was performed via a secure web-based randomisation system written and hosted by NPEU CTU with 24/7 telephone back-up, ensuring allocation concealment. The randomisation program used a probabilistic minimisation algorithm (allocation ratio 1:1) to ensure balance between the groups for the size of the PDA, gestational age at birth, age, sex, site, multiple births, mode of respiratory support and receiving inotropes. Multiple births were randomised individually. Participants were enrolled by the delegated clinician at the study site.

## Two year follow up form

The Baby-OSCAR 2 Year Form can be downloaded from <https://www.isrctn.com/ISRCTN84264977> (Additional files) or requested from the first author.

## Multiple imputation of missing primary outcome data

A multiple imputation analysis was performed for the non-verbal cognitive and language PARCA-R scores for infants with PARCA-R questionnaires completed outside of the 23.5 to 27.5 months corrected age range, or for those with more than 4 missing non-verbal cognitive scale questions missing. Infants with a cognitive or language classification by the Blinded Endpoint Review Committee were not included in the multiple imputation analysis of that component.

The (continuous) value of the standardised PARCA-R score was imputed by predictive mean matching, using the closest 10 observations as donors. A total of 50 imputation datasets were generated and used to convert the imputed standardised scores to the required binary outcome. The multiple imputation models included minimisation factors and other baseline variables associated with PARCA-R raw score status. These were: PDA size at randomisation, gestational age at birth, post-natal age at randomisation (in hours), sex, multiparity, mode of respiratory support at randomisation, any inotropes given at randomisation, and recruiting site. This process was repeated separately for the cognitive score and for the language score.

A second senior statistician validated the multiple imputation process and outputs

# Tables

Table S1: Additional baseline characteristics for children for whom long-term outcomes were assessed

| Characteristic | Ibuprofen  (n=263) | Placebo  (n=274) |
| --- | --- | --- |
| Maternal characteristics | | |
| **Antenatal steroid use, n (%)** |  |  |
| Any | 243 (92.7) | 245 (90.4) |
| < 24 hours before birth^1^ | 86 (35.4) | 84 (34.3) |
| ≥ 24 hours before birth | 157 (64.6) | 161 (65.7) |
| Missing, n | 1 | 3 |
| **Antenatal COX inhibitor use, n (%)** | 38 (14.7) | 34 (12.6) |
| Missing, n | 4 | 4 |
| **Antenatal magnesium sulphate use for neuroprotection, n (%)** | 194 (77.0) | 209 (79.8) |
| Missing, n | 11 | 12 |
| Infant characteristics at randomization | | |
| **Born in enrolling centre, n (%)** | 225 (85.6) | 240 (87.6) |
| **Enrolling centre*, n (%)** |  |  |
| 1 | 27 (10.3) | 26 (9.5) |
| 2 | 13 (4.9) | 16 (5.8) |
| 3 | 20 (7.6) | 19 (6.9) |
| 4 | 15 (5.7) | 16 (5.8) |
| 5 | 16 (6.1) | 13 (4.7) |
| 6 | 17 (6.5) | 13 (4.7) |
| 7 | 14 (5.3) | 18 (6.6) |
| 8 | 12 (4.6) | 16 (5.8) |
| 9 | 9 (3.4) | 15 (5.5) |
| 10 | 13 (4.9) | 10 (3.6) |
| 11 | 8 (3.0) | 8 (2.9) |
| 12 | 11 (4.2) | 8 (2.9) |
| 13 | 8 (3.0) | 5 (1.8) |
| 14 | 8 (3.0) | 8 (2.9) |
| 15 | 7 (2.7) | 10 (3.6) |
| 16 | 3 (1.1) | 8 (2.9) |
| 17 | 10 (3.8) | 5 (1.8) |
| 18 | 6 (2.3) | 6 (2.2) |
| 19 | 5 (1.9) | 7 (2.6) |
| 20 | 5 (1.9) | 5 (1.8) |
| 21 | 4 (1.5) | 8 (2.9) |
| 22 | 9 (3.4) | 3 (1.1) |
| 23 | 3 (1.1) | 7 (2.6) |
| 24 | 3 (1.1) | 6 (2.2) |
| 25 | 3 (1.1) | 6 (2.2) |
| 26 | 5 (1.9) | 4 (1.5) |
| 27 | 2 (0.8) | 4 (1.5) |
| 28 | 3 (1.1) | 1 (0.4) |
| 29 | 2 (0.8) | 1 (0.4) |
| 30 | 1 (0.4) | 1 (0.4) |
| 31 | 1 (0.4) | 1 (0.4) |
| Gestational age at birth (weeks)*, n (%) |  |  |
| 23 to < 24 weeks | 24 (9.1) | 24 (8.8) |
| 24 to < 25 weeks | 51 (19.4) | 50 (18.2) |
| 25 to < 26 weeks | 52 (19.8) | 52 (19.0) |
| 26 to < 27 weeks | 54 (20.5) | 56 (20.4) |
| 27 to < 28 weeks | 48 (18.3) | 49 (17.9) |
| 28 to < 29 weeks | 34 (12.9) | 42 (15.3) |
| ≥ 29 weeks | 0 (0.0) | 1 (0.4) |
| Forceps or Ventouse used in delivery, n (%) | 4 (1.5) | 2 (0.7) |
| Missing, n | 0 | 2 |
| Main cause of preterm birth, n (%) |  |  |
| Preterm pre-labour rupture of membranes (PPROM) | 95 (36.1) | 82 (29.9) |
| Preterm labour (without PROM) | 89 (33.8) | 104 (38.0) |
| Antepartum hemorrhage^2^ | 25 (9.5) | 31 (11.3) |
| Hypertension^3^ | 5 (1.9) | 5 (1.8) |
| Pre-eclampsia | 8 (3.0) | 13 (4.7) |
| Sepsis | 6 (2.3) | 5 (1.8) |
| Other maternal illness | 8 (3.0) | 5 (1.8) |
| Obstetric intervention for fetal reasons | 27 (10.3) | 29 (10.6) |
| Birth weight z score |  |  |
| Mean (SD) | -0.4 (0.8) | -0.4 (0.8) |
| Head circumference (cm) |  |  |
| Mean (SD) | 23.5 (2.2) | 23.5 (2.0) |
| Missing, n | 86 | 113 |
| Head circumference z score |  |  |
| Mean (SD) | -0.9 (1.1) | -0.9 (1.0) |
| Missing, n | 86 | 113 |
| Baby is one of a multiple pregnancy*, n (%) | 77 (29.3) | 75 (27.4) |
| Families in the trial (sets of babies from a multiple pregnancy and singletons), n | 242 | 249 |
| Sets of babies from a multiple pregnancy in the trial, n | 21 | 24 |
| Baby’s worst base excess at first hour after birth |  |  |
| Mean (SD) | -5.8 (4.3) | -5.3 (4.4) |
| Missing, n | 65 | 56 |
| CRIB II^4^ (without temperature) |  |  |
| Mean (SD) | 11.2 (2.6) | 10.8 (2.7) |
| Missing, n | 65 | 56 |

* Denotes factor used in the randomization minimization algorithm

SD denotes standard deviation and IQR interquartile range.

^1^ Doses would usually be 24 hours apart, so < 24 hours before birth suggests only one dose was taken.

^2^ Including abnormally implanted placenta.

^3^ With or without antepartum hemorrhage.

^4^ Clinical Risk Index for Babies.

Table S2: Risk factors for respiratory morbidity collected at 2-year follow-up

| Risk factor | Ibuprofen  (n=263) | Placebo  (n=274) |
| --- | --- | --- |
| Family history of asthma or wheezing, n(%) | 65 (38.2) | 75 (38.9) |
| Missing | 93 | 81 |
| Smoking in household | 12 (7.2) | 11 (5.9) |
| Missing | 96 | 86 |
| Problems with dust, damp or mould, or major building work or renovations in the home | 17 (10.2) | 21 (11.1) |
| Missing | 97 | 85 |
| Living near a busy main road | 41 (24.7) | 52 (27.5) |
| Missing | 97 | 85 |

Table S3: Baseline characteristics by availability of 2-year follow-up data

(Available if 2 year follow-up questionnaire was returned or if main long-term outcome had been classified by the Blinded Endpoint Review Committee or if the child was known to have died)

|  | Ibuprofen | | Placebo | |
| --- | --- | --- | --- | --- |
| Characteristic | Available  (n=263) | Not available (n=61) | Available  n=274) | Not available (n=48) |
| Maternal characteristics | | | | |
| Mother’s ethnicity, n (%) |  |  |  |  |
| White | 178 (73.0) | 45 (81.8) | 190 (73.4) | 33 (75.0) |
| Asian | 34 (13.9) | 5 (9.1) | 41 (15.8) | 4 (9.1) |
| Black | 22 (9.0) | 3 (5.5) | 21 (8.1) | 4 (9.1) |
| Other | 10 (4.1) | 2 (3.6) | 7 (2.7) | 3 (6.8) |
| Missing, n | 19 | 6 | 15 | 4 |
| Mother’s age (years) |  |  |  |  |
| Mean (SD) | 30.9 (6.4) | 26.6 (5.5) | 30.7 (5.9) | 26.9 (6.6) |
| Deprivation index ^1^, n (%) |  |  |  |  |
| 1 (least deprived) | 79 (35.9) | 24 (47.1) | 84 (34.6) | 17 (43.6) |
| 2 | 50 (22.7) | 7 (13.7) | 61 (25.1) | 14 (35.9) |
| 3 | 41 (18.6) | 11 (21.6) | 38 (15.6) | 2 (5.1) |
| 4 | 30 (13.6) | 7 (13.7) | 37 (15.2) | 4 (10.3) |
| 5 (most deprived) | 20 (9.1) | 2 (3.9) | 23 (9.5) | 2 (5.1) |
| Missing or not defined, n | 43 | 10 | 31 | 9 |
| Antenatal steroid use, n (%) |  |  |  |  |
| Any | 243 (92.7) | 50 (82.0) | 245 (90.4) | 45 (93.8) |
| < 24 hours before birth^2^ | 86 (35.4) | 15 (30.0) | 84 (34.3) | 18 (40.0) |
| ≥ 24 hours before birth | 157 (64.6) | 35 (70.0) | 161 (65.7) | 27 (60.0) |
| Missing, n | 1 | 0 | 3 | 0 |
| Antenatal COX inhibitor use, n (%) | 38 (14.7) | 5 (8.5) | 34 (12.6) | 2 (4.3) |
| Missing, n | 4 | 2 | 4 | 1 |
| Antenatal magnesium sulphate use for neuroprotection, n (%) | 194 (77.0) | 42 (72.4) | 209 (79.8) | 36 (76.6) |
| Missing, n | 11 | 3 | 12 | 1 |
| Infant characteristics at randomization | | | | |
| Born in enrolling centre, n (%) | 225 (85.6) | 48 (78.7) | 240 (87.6) | 37 (77.1) |
| Enrolling centre*, n (%) |  |  |  |  |
| 1 | 27 (10.3) | 3 (4.9) | 26 (9.5) | 3 (6.3) |
| 2 | 13 (4.9) | 15 (24.6) | 16 (5.8) | 8 (16.7) |
| 3 | 20 (7.6) | 2 (3.3) | 19 (6.9) | 1 (2.1) |
| 4 | 15 (5.7) | 4 (6.6) | 16 (5.8) | 3 (6.3) |
| 5 | 16 (6.1) | 1 (1.6) | 13 (4.7) | 3 (6.3) |
| 6 | 17 (6.5) | 2 (3.3) | 13 (4.7) | 1 (2.1) |
| 7 | 14 (5.3) | 1 (1.6) | 18 (6.6) | 0 (0.0) |
| 8 | 12 (4.6) | 1 (1.6) | 16 (5.8) | 3 (6.3) |
| 9 | 9 (3.4) | 5 (8.2) | 15 (5.5) | 2 (4.2) |
| 10 | 13 (4.9) | 1 (1.6) | 10 (3.6) | 0 (0.0) |
| 11 | 8 (3.0) | 3 (4.9) | 8 (2.9) | 3 (6.3) |
| 12 | 11 (4.2) | 1 (1.6) | 8 (2.9) | 1 (2.1) |
| 13 | 8 (3.0) | 3 (4.9) | 5 (1.8) | 4 (8.3) |
| 14 | 8 (3.0) | 0 (0.0) | 8 (2.9) | 2 (4.2) |
| 15 | 7 (2.7) | 0 (0.0) | 10 (3.6) | 0 (0.0) |
| 16 | 3 (1.1) | 2 (3.3) | 8 (2.9) | 3 (6.3) |
| 17 | 10 (3.8) | 0 (0.0) | 5 (1.8) | 1 (2.1) |
| 18 | 6 (2.3) | 2 (3.3) | 6 (2.2) | 2 (4.2) |
| 19 | 5 (1.9) | 1 (1.6) | 7 (2.6) | 2 (4.2) |
| 20 | 5 (1.9) | 3 (4.9) | 5 (1.8) | 1 (2.1) |
| 21 | 4 (1.5) | 1 (1.6) | 8 (2.9) | 0 (0.0) |
| 22 | 9 (3.4) | 0 (0.0) | 3 (1.1) | 0 (0.0) |
| 23 | 3 (1.1) | 1 (1.6) | 7 (2.6) | 1 (2.1) |
| 24 | 3 (1.1) | 2 (3.3) | 6 (2.2) | 1 (2.1) |
| 25 | 3 (1.1) | 2 (3.3) | 6 (2.2) | 0 (0.0) |
| 26 | 5 (1.9) | 1 (1.6) | 4 (1.5) | 0 (0.0) |
| 27 | 2 (0.8) | 2 (3.3) | 4 (1.5) | 0 (0.0) |
| 28 | 3 (1.1) | 0 (0.0) | 1 (0.4) | 1 (2.1) |
| 29 | 2 (0.8) | 1 (1.6) | 1 (0.4) | 1 (2.1) |
| 30 | 1 (0.4) | 1 (1.6) | 1 (0.4) | 1 (2.1) |
| 31 | 1 (0.4) | 0 (0.0) | 1 (0.4) | 0 (0.0) |
| Postnatal age, (hours)*, n (%) |  |  |  |  |
| Median [IQR] | 57.3 [42.7 to 65.7] | 58.0 [45.4 to 64.5] | 56.1 [42.8 to 66.1] | 62.8 [50.9 to 69.1] |
| < 12 hours | 1 (0.4) | 1 (1.6) | 2 (0.7) | 0 (0.0) |
| 12 to < 24 hours | 11 (4.2) | 4 (6.6) | 12 (4.4) | 2 (4.2) |
| 24 to < 48 hours | 77 (29.3) | 13 (21.3) | 80 (29.2) | 9 (18.8) |
| 48 to < 72 hours | 174 (66.2) | 43 (70.5) | 180 (65.7) | 37 (77.1) |
| Gestational age at birth (weeks)*, n (%) |  |  |  |  |
| Mean (SD) | 26.0 (1.5) | 26.1 (1.5) | 26.1 (1.6) | 26.0 (1.5) |
| 23 to < 24 weeks | 24 (9.1) | 6 (9.8) | 24 (8.8) | 5 (10.4) |
| 24 to < 25 weeks | 51 (19.4) | 8 (13.1) | 50 (18.2) | 8 (16.7) |
| 25 to < 26 weeks | 52 (19.8) | 14 (23.0) | 52 (19.0) | 11 (22.9) |
| 26 to < 27 weeks | 54 (20.5) | 12 (19.7) | 56 (20.4) | 12 (25.0) |
| 27 to < 28 weeks | 48 (18.3) | 13 (21.3) | 49 (17.9) | 7 (14.6) |
| 28 to < 29 weeks | 34 (12.9) | 8 (13.1) | 42 (15.3) | 5 (10.4) |
| ≥ 29 weeks | 0 (0.0) | 0 (0.0) | 1 (0.4) | 0 (0.0) |
| Mode of birth, n (%) |  |  |  |  |
| Vaginal birth – cephalic | 108 (41.1) | 33 (54.1) | 117 (42.7) | 21 (43.8) |
| Vaginal birth – breech | 43 (16.3) | 7 (11.5) | 37 (13.5) | 9 (18.8) |
| Caesarean section before onset of labour | 70 (26.6) | 13 (21.3) | 71 (25.9) | 9 (18.8) |
| Caesarean section after onset of labour | 42 (16.0) | 8 (13.1) | 49 (17.9) | 9 (18.8) |
| Forceps or Ventouse used in delivery, n (%) | 4 (1.5) | 0 (0.0) | 2 (0.7) | 0 (0.0) |
| Main cause of preterm birth, n (%) |  |  |  |  |
| Preterm pre-labour rupture of membranes (PPROM) | 95 (36.1) | 17 (27.9) | 82 (29.9) | 19 (39.6) |
| Preterm labour (without PROM) | 89 (33.8) | 27 (44.3) | 104 (38.0) | 14 (29.2) |
| Antepartum hemorrhage^3^ | 25 (9.5) | 9 (14.8) | 31 (11.3) | 8 (16.7) |
| Hypertension^4^ | 5 (1.9) | 1 (1.6) | 5 (1.8) | 0 (0.0) |
| Pre-eclampsia | 8 (3.0) | 0 (0.0) | 13 (4.7) | 2 (4.2) |
| Sepsis | 6 (2.3) | 1 (1.6) | 5 (1.8) | 1 (2.1) |
| Other maternal illness^5^ | 8 (3.0) | 2 (3.3) | 5 (1.8) | 0 (0.0) |
| Obstetric intervention for fetal reasons | 27 (10.3) | 4 (6.6) | 29 (10.6) | 4 (8.3) |
| Birth weight (g) |  |  |  |  |
| Mean (SD) | 836.1 (207.7) | 856.1 (192.6) | 850.8 (211.8) | 864.7 (210.0) |
| Birth weight z score |  |  |  |  |
| Mean (SD) | -0.4 (0.8) | -0.4 (0.7) | -0.4 (0.8) | -0.2 (0.8) |
| Head circumference (cm) |  |  |  |  |
| Mean (SD) | 23.5 (2.2) | 23.9 (2.4) | 23.5 (2.0) | 23.8 (2.1) |
| Missing, n | 86 | 23 | 113 | 22 |
| Head circumference z score |  |  |  |  |
| Mean (SD) | -0.9 (1.1) | -0.6 (1.2) | -0.9 (1.0) | -0.8 (1.0) |
| Missing, n | 86 | 23 | 113 | 22 |
| Sex*, n (%) |  |  |  |  |
| Male | 145 (55.1) | 35 (57.4) | 147 (53.6) | 28 (58.3) |
| Baby is one of a multiple pregnancy*, n (%) | 77 (29.3) | 11 (18.0) | 75 (27.4) | 14 (29.2) |
| Families in the trial (sets of babies from a multiple pregnancy and singletons), n | 242 | 57 | 249 | 43 |
| Sets of babies from a multiple pregnancy in the trial, n | 21 | 4 | 24 | 5 |
| Sibling enrolled in the study (in multiple pregnancies), n (%) | 18 (23.4) | 6 (54.5) | 28 (37.3) | 6 (42.9) |
| APGAR score 5 minutes after birth, n (%) |  |  |  |  |
| Median [IQR] | 8 [6 to 9] | 8 [6 to 9] | 7 [6 to 9] | 7 [5 to 8] |
| Missing, n | 34 | 12 | 29 | 5 |
| Baby’s worst base excess at first hour after birth |  |  |  |  |
| Mean (SD) | -5.8 (4.3) | -4.9 (4.3) | -5.3 (4.4) | -7.2 (5.0) |
| Missing, n | 65 | 20 | 56 | 14 |
| CRIB II^6^ (without temperature) |  |  |  |  |
| Mean (SD) | 11.2 (2.6) | 10.8 (2.5) | 10.8 (2.7) | 11.5 (2.7) |
| Missing, n | 65 | 20 | 56 | 14 |
| Size of Patent Ductus Arteriosus (PDA)*, n (%) |  |  |  |  |
| Median [IQR] | 2.2 [1.9 to 2.5] | 2.2 [2.0 to 2.6] | 2.1 [1.9 to 2.6] | 2.3 [2.0 to 2.8] |
| ≥ 1.5 mm and < 2.0 mm | 70 (26.6) | 14 (23.0) | 70 (25.5) | 12 (25.0) |
| ≥ 2.0 mm and < 3.0 mm | 160 (60.8) | 41 (67.2) | 170 (62.0) | 31 (64.6) |
| ≥ 3.0 mm | 33 (12.5) | 6 (9.8) | 34 (12.4) | 5 (10.4) |
| Mode of respiratory support at randomisation*, n (%) |  |  |  |  |
| Invasive ventilation (by endotracheal tube) | 166 (63.1) | 40 (65.6) | 175 (63.9) | 29 (60.4) |
| Non-invasive respiratory support only^7^ | 95 (36.1) | 21 (34.4) | 97 (35.4) | 18 (37.5) |
| Receiving no mechanical ventilation or  pressure support^8^ | 2 (0.8) | 0 (0.0) | 2 (0.7) | 1 (2.1) |
| Receiving inotropes*, n (%) | 38 (14.4) | 6 (9.8) | 26 (9.5) | 11 (22.9) |
| * Denotes factor used in the randomization minimization algorithm.  SD denotes standard deviation and IQR interquartile range.  ^1^ Combines information from seven domains to produce an overall relative measure of deprivation. The domains are income; employment; education; skills and training; health and disability; crime; barriers to housing and services; and living environment.  ^2^ Doses would usually be 24 hours apart, so < 24 hours before birth suggests only one dose was taken.  ^3^ Including abnormally implanted placenta.  ^4^ With or without antepartum hemorrhage.  ^5^ Any pregnancy where the main reason for preterm delivery was a maternal problem such as infection, renal disease or pre-pregnancy diabetes, hypertension or trauma.  ^6^ Clinical Risk Index for Babies.  ^7^ Nasal continuous positive airway pressure, nasal ventilation, humidified high flow nasal cannula therapy, or low flow oxygen ≥ 1.1L/min.  ^8^ In room air, low flow oxygen < 1.1L/min, or ambient oxygen. | | | | |

Table S4: Short-term outcomes by availability of 2-year follow-up data

(Available if 2 year follow-up questionnaire was returned or if main long-term outcome had been ascertained by the Blinded Endpoint Review Committee or if the child was known to have died)

|  | Ibuprofen | | Placebo | |
| --- | --- | --- | --- | --- |
| Short-term outcome (up to discharge or 36 weeks’ postmenstrual age) | Available  (n=263) | Not available  (n=61) | Available  (n=274) | Not available  (n=48) |
| Death by or moderate/ severe bronchopulmonary dysplasia (BPD) at 36 weeks’ postmenstrual age^1^, n(%) | 181 (69.9) | 39 (66.1) | 169 (62.6) | 33 (68.8) |
| Death by 36 weeks’ postmenstrual age, n | 44 | 0 | 33 | 0 |
| Infants survived up to 36 weeks’ postmenstrual age, n | 215 | 59 | 237 | 48 |
| Missing | 4 | 2 | 4 | 0 |
| Moderate or severe BPD at 36 weeks’ postmenstrual age, n(%) | 137 (63.7) | 39 (66.1) | 136 (57.4) | 33 (68.8) |
| Severe intraventricular haemorrhage (grade III/ IV^2^), n(%) | 38 (14.4) | 7 (11.5) | 26 (9.5) | 8 (16.7) |
| Cystic periventricular leukomalacia, n(%) | 14 (5.3) | 1 (1.6) | 8 (2.9) | 1 (2.1) |
| Treated for retinopathy of prematurity^3^, n(%) | 38 (14.4) | 7 (11.5) | 37 (13.5) | 8 (16.7) |
| NEC Bell stage II and above^4^, n(%) | 39 (14.9) | 5 (8.2) | 40 (14.6) | 3 (6.3) |
| Missing, n | 1 | 0 | 0 | 0 |
| Closed or non-significant PDA (< 1.5mm) at around 3 weeks of age, confirmed by ECHO^5^, n(%) | 136 (52.9) | 40 (66.7) | 93 (34.7) | 24 (50.0) |
| Missing, n | 6 | 1 | 6 | 0 |
| Discharge home on oxygen, n(%) | 99 (38.2) | 31 (55.4) | 103 (38.3) | 20 (44.4) |
| Missing, n | 4 | 5 | 5 | 3 |
| Postnatal steroid use for chronic lung disease, n(%) | 64 (24.4) | 21 (34.4) | 67 (24.5) | 15 (31.3) |
| Missing, n | 1 | 0 | 0 | 0 |
| ^1^ Short-term primary outcome.  ^2^ With ventricular dilation or intraparenchymal abnormality.  ^3^ In at least one eye.  ^4^ Confirmed by radiography and/or histopathology.  ^5^ Deviation from the Statistical Analysis Plan due to incorrect short-term outcome specified. | | | | |

Table S5: Components of main long-term outcome – additional detail

|  | Ibuprofen  (n=263) | Placebo  (n=274) |
| --- | --- | --- |
| Children survived | n=211 | n=232 |
| Non-verbal cognitive impairment, n (%) |  |  |
| Moderate or severe | 38 (22.2) | 50 (24.9) |
| PARCA-R < 70 | 28 (73.7) | 40 (80.0) |
| Classified by BERC | 10 (26.3) | 10 (20.0) |
| Mild or no impairment | 133 (77.8) | 151 (75.1) |
| PARCA-R ≥ 70 | 103 (77.4) | 117 (77.5) |
| Classified by BERC | 30 (22.6) | 34 (22.5) |
| Not known^1^, n | 40 | 31 |
| Language cognitive impairment, n (%) |  |  |
| Moderate or severe | 30 (19.1) | 41 (22.9) |
| PARCA-R < 70 | 18 (60.0) | 26 (63.4) |
| Classified by BERC | 12 (40.0) | 15 (36.6) |
| Mild or no impairment | 127 (80.9) | 138 (77.1) |
| PARCA-R ≥ 70 | 98 (77.2) | 109 (79.0) |
| Classified by BERC | 29 (22.8) | 29 (21.0) |
| Not known^1^, n | 54 | 53 |
| Gross motor impairment, n (%) |  |  |
| Moderate or severe | 23 (11.0) | 20 (8.7) |
| Parent report | 14 (60.9) | 12 (60.0) |
| Classified by BERC | 9 (39.1) | 8 (40.0) |
| Mild or no impairment | 186 (89.0) | 211 (91.3) |
| Parent report | 154 (82.8) | 175 (82.9) |
| Classified by BERC | 32 (17.2) | 36 (17.1) |
| Not known^1^, n | 2 | 1 |
| Hearing impairment, n (%) |  |  |
| Moderate or severe | 4 (1.9) | 4 (1.7) |
| Parent report | 4 (100.0) | 4 (100.0) |
| Classified by BERC | 0 (0.0) | 0 (0.0) |
| Mild or no impairment | 203 (98.1) | 227 (98.3) |
| Parent report | 164 (80.8) | 183 (80.6) |
| Classified by BERC | 39 (19.2) | 44 (19.4) |
| Not known^1^, n | 4 | 1 |
| Visual impairment, n (%) |  |  |
| Moderate or severe | 8 (3.9) | 7 (3.0) |
| Parent report | 5 (62.5) | 7 (100.0) |
| Classified by BERC | 3 (37.5) | 0 (0.0) |
| Mild or no impairment | 198 (96.1) | 223 (97.0) |
| Parent report | 163 (82.3) | 179 (80.3) |
| Classified by BERC | 35 (17.7) | 44 (19.7) |
| Not known^1^, n | 5 | 2 |
| ^1^ Comprises follow-up questionnaire completed outside the age range for standardization for PARCA-R, or completed but with insufficient data, and with no classification by the Blinded Endpoint Review Committee. | | |

Table S6: Components of respiratory morbidity outcomes – additional detail

|  | Ibuprofen (n=263) | Placebo (n=274) |
| --- | --- | --- |
| Duration of oxygen supplementation from randomisation (days)^1^ |  |  |
| Mean (SD) | 148.9 (191.7) | 138.6 (167.9) |
| Median [IQR] | 76.0 [38.0 to 166.0] | 78.0 [46.0 to 156.0] |
| (Min to max) | (3 to 841) | (2 to 830) |
| Missing, n | 20 | 24 |
| Children survived | n=211 | n=232 |
| Need for oxygen or respiratory support, n (%) | 107 (64.8) | 120 (64.5) |
| Missing, n | 46 | 46 |
| Discharged home on oxygen | 81 (77.1) | 97 (72.5) |
| Missing, n | 2 | 0 |
| Still on oxygen | 10 (9.5) | 6 (5.0) |
| Missing, n | 2 | 0 |
| Received any oxygen at other time(s) since discharge | 22 (59.5) | 25 (61.0) |
| Missing, n | 70 | 79 |
| Need for respiratory support2, n (%) | 31 (38.3) | 36 (35.5) |
| Ventilator | 5 (16.7) | 15 (41.7) |
| CPAP | 9 (30.0) | 5 (13.9) |
| Tracheostomy | 1 (3.3) | 0 (0.0) |
| Other | 15 (50.0) | 16 (44.4) |
| Missing, n | 1 | 0 |
| Presence of a persistent cough and/or wheeze, n (%) | 46 (26.7) | 51 (26.4) |
| Missing, n | 39 | 39 |
| Persistent cough | 28 (60.9) | 28 (54.9) |
| Affects feeding, n (%) | 12 (44.4) | 12 (44.4) |
| Missing | 1 | 1 |
| Affects sleep, n (%) | 23 (85.2) | 21 (77.8) |
| Missing | 1 | 1 |
| Affects physical activity, n (%) | 17 (60.7) | 13 (50.0) |
| Missing | 0 | 2 |
| Persistent wheeze | 40 (87.0) | 45 (88.2) |
| Need for regular treatment for respiratory illness, n (%) | 94 (54.7) | 97 (50.3) |
| Missing, n | 39 | 39 |
| Inhaler – reliever | 62 (70.5) | 66 (70.2) |
| Missing, n | 6 | 3 |
| Inhaler – preventer | 15 (19.7) | 28 (30.8) |
| Missing, n | 18 | 6 |
| Steroids | 31 (39.7) | 33 (36.3) |
| Missing, n | 16 | 6 |
| Other | 40 (50.0) | 36 (39.1) |
| Missing, n | 14 | 5 |
| Unscheduled attendances at hospital/ GP for respiratory problems, n(%) |  |  |
| 0 attendances | 66 (38.4) | 67 (35.1) |
| 1 – 3 attendances | 67 (39.0) | 68 (35.6) |
| 4 – 12 attendances | 30 (17.4) | 51 (26.7) |
| More than 12 attendances | 9 (5.2) | 5 (2.6) |
| Missing, n | 39 | 41 |
| Re-hospitalisation for respiratory problems, n(%) |  |  |
| 0 admissions | 79 (51.0) | 85 (47.5) |
| 1 – 2 admissions | 51 (32.9) | 56 (31.3) |
| 3 – 5 admissions | 15 (9.7) | 25 (14.0) |
| More than 5 admissions | 10 (6.5) | 13 (7.3) |
| Missing, n | 56 | 53 |
| ^1^ Duration of oxygen supplementation from randomisation has been upper bounded so that the number of days on oxygen cannot exceed the number of days between date of randomisation and date 2-years corrected age is reached  ^2^ Parent reported: “Since first discharge from hospital, has your child received any other breathing support?”. Multiple selections are possible. | | |
