# Supplementary material for: Two-year outcomes after selective early treatment of patent ductus arteriosus with ibuprofen in preterm babies: follow-up of Baby-OSCAR–a randomised controlled trial
Source: eClinicalMedicine. 2025 Aug 20;87:103424. doi: 10.1016/j.eclinm.2025.103424 (PMC12396396; doi:10.1016/j.eclinm.2025.103424)
Supplement: Protocol [file mmc2.pdf]

This supplement contains the following items:

1. Original protocol, final protocol, summary of changes.
2. Original statistical analysis plan and final statistical analysis plan, summary of changes.

Baby-OSCAR Protocol and Statistical Analysis Plan (SAP)

Table of Contents

Original protocol 3

Final protocol 51

Summary of all protocol amendments 100

Original and final Statistical Analysis Plan (SAP) for long term outcomes 106

Summary of SAP amendments 125

Outcome after Selective Early Treatment for Closure of Patent Ductus ARteriosus in Preterm Babies

PROTOCOL

Chief Investigator:

Professor Samir Gupta

Consultant Neonatologist University Hospital of North Tees

Hardwick Road

Stockton-on-Tees TS19 6PE

Tel: 01642 624232

Clinical Trials Unit:

National Perinatal Epidemiology Unit

Nuffield Department of Population Health University of Oxford

Old Road Campus Oxford

OX3 7LF

Tel: 01865 289700

Funder:

National Institute of Health Research – Health Technology Assessment Programme (project reference 11/92/15)

Sponsor:

University of Oxford

Trial Identifiers:

EudraCT No.: 2013-005336-23

ISRCTN: 84264977

Professor Samir Gupta

26/01/15

Chief Investigator	Chief Investigator's Signature	Date

Table of Contents

1. Protocol Synopsis 5
2. Trial Flow Diagram 10
3. Abbreviations 11
4. Introduction 13
   4.1. Background and Rationale 13
   4.2. Current Evidence Supporting Trial Rationale 14
5. Trial Objective 15
   5.1. Primary Objective 15
   5.2. Secondary Objectives 16
6. Trial Design 16
   6.1. Summary 16
   6.2. Inclusion Criteria 17
   6.3. Exclusion Criteria 17
   6.4. Setting 17
   6.5. Primary Outcome 18
      6.5.1. Oxygen Reduction Test 19
   6.6. Secondary Outcomes 19
   6.7. Process Outcomes 21
7. Trial Procedures 21
   7.1. Trial Assessments 21
   7.2. Structure and Duration of the Trial 22
   7.3. Initial Eligibility Assessment 23
   7.4. Informed Consent 23
   7.5. Randomisation 24
   7.6. Echocardiograms 24
   7.7. Concomitant Medications 25
   7.8. Permitted and Non-Permitted Medications 25
      7.8.1. Supportive Care of Enrolled Babies 25
   7.9. Stopping Trial Interventions 26
   7.10. End of Trial 26
   7.11. Early Trial Cessation 26
   7.12. Remuneration 26
8. Investigational Medicinal Product (IMP) 26
   8.1. Dosing and Administration 27
   8.2. Distribution 27
   8.3. Accountability 27
   8.4. Rescue Treatment 27
   8.5. Masking of Trial Medication 28
   8.6. Procedure for Unmasking 28
9. Safety Reporting 29

9.1. Definitions ... 29
9.1.1. Adverse Event (AE) ... 29
9.1.2. Adverse Reaction (AR) ... 29
9.1.3. Serious Adverse Event (SAE) ... 29
9.1.4. Foreseeable Serious Adverse Events ... 29
9.1.5. Unforeseeable Serious Adverse Events ... 30
9.1.6. Serious Adverse Reaction (SAR) ... 30
9.1.7. Suspected Unexpected Serious Adverse Reaction (SUSAR) ... 31
9.1.8. Causality ... 31
9.1.9. Assessment of Safety ... 31
9.2. Reporting Procedures ... 31
9.2.1. AE/SAE Reporting ... 31
9.2.2. SUSAR Reporting ... 32
9.2.3. Development Safety Update Report (DSUR) ... 32
10. Statistics and Analysis ... 32
10.1. Sample Size ... 32
10.2. Assessment of Outcomes ... 34
10.3. Statistical Analysis ... 35
10.3.1. Primary Analysis Population ... 35
10.3.2. Statistical Methods ... 35
10.3.3. Pre-specified Subgroup Analysis ... 36
10.3.4. Level of Statistical Significance ... 36
10.3.5. Dealing with Missing Data ... 36
10.4. Economic Analysis ... 36
10.5. Measures to Minimise Bias ... 37
11. Source Data/Documents ... 37
12. Quality Control and Assurance ... 37
12.1. Risk Assessment ... 37
12.2. National Registration Systems ... 38
12.3. Site Initiation and Training ... 38
12.4. Site Monitoring and Auditing ... 38
12.5. Blinded Endpoint Review ... 38
13. Serious Breach of Good Clinical Practice or the Trial Protocol ... 39
14. Ethics ... 39
14.1. Declaration of Helsinki ... 39
14.2. Guidelines for Good Clinical Practice ... 39
14.3. Approvals ... 39
14.4. Participant Confidentiality, Data Handling and Record Keeping ... 40
14.5. Retention of Personal Data ... 40
14.6. Funding ... 41
14.7. Insurance ... 41
15. Trial Governance ... 41

15.1. Site Research and Development Approval 41
15.2. Trial Sponsor 41
15.3. Co-ordinating Centre 41
15.4. Project Management Group (PMG) 41
15.5. Co-investigators' Group (CIG) 42
15.6. Trial Steering Committee (TSC) 42
15.7. Data Monitoring Committee (DMC) 42
16. Publication Policy/Acknowledgement of Contribution 43
17. Protocol Signature 44
17.1. Principal Investigator Signature 44
18. References 45
Contact details 48

1. Protocol Synopsis

**Trial Title:**	Outcome after Selective Early Treatment for Closure of Patent Ductus ARteriosus in Preterm Babies [Baby-OSCAR Trial]
**Internal Reference:**	Baby-OSCAR
**Clinical Phase:**	Phase III
**Trial Design:**	Multi-centre, masked, randomised placebo-controlled parallel group trial to determine short and long term health and economic outcomes of the treatment of a large Patent Ductus Arteriosus (PDA) in extremely preterm babies with ibuprofen within 72 hours of birth.<br><br>The main trial will be conducted after an internal pilot phase, which will be run to assess the suitability of trial procedures and likelihood of recruitment targets being achieved.
**Trial Participants:**	Extreme preterm babies with a large PDA confirmed using echocardiography.
**Inclusion Criteria:**	Babies will be considered eligible for inclusion in the trial if they are:<br>• Born at 23^{+0} to 28^{+6} weeks of gestation<br>• Less than 72 hours old<br>• Confirmed by echocardiography to have a large PDA which<br>- is at least 1.5 mm in diameter (determined by gain optimised colour Doppler),<br>*and*<br>- has unrestrictive pulsatile left to right flow (ratio of flow velocity in PDA Maximum (V_{max}) to Minimum (V_{min}) > 2:1))<br>In addition:<br>• The responsible clinician is uncertain about whether the baby might benefit from treatment to close the PDA<br>• Written informed consent has been obtained from the parent(s).
**Exclusion Criteria:**	Babies will be excluded from participation in the trial if they have:<br>• No realistic prospect of survival<br>• Severe congenital anomaly<br>• Clinical or echocardiography suspicion of congenital structural heart disease that contraindicates treatment with ibuprofen<br>• Other conditions that would contraindicate the use of ibuprofen (Clinically significant intracranial or gastrointestinal haemorrhage,

	coagulopathy, thrombocytopenia (platelet count <50,000), renal failure, pulmonary hypertension, known or suspected necrotising enterocolitis (NEC))<br>• Indomethacin, ibuprofen, or paracetamol administration after birth
**Sample Size:**	Approximately 730 preterm babies in total (including those recruited during the internal pilot phase).<br>365 babies per treatment arm.
**Trial Sites:**	25 UK tertiary (level 3) neonatal units. 4 sites for the internal pilot phase.
**Trial Period:**	Trial period for an individual baby is defined as randomisation to 2 years of age corrected for prematurity.<br>For the purposes of regulatory notification, end of trial is defined as the last follow-up assessment at 2 years of age corrected for prematurity. The entire trial is anticipated to take 82 months to complete (including set-up, internal pilot phase and reporting).<br>Adverse Events which are serious will be recorded from first dose until 7 days after trial medication. Only Unforeseeable SAEs will be reported.
**Primary Objective:**	To determine if the selective treatment of echocardiographically confirmed large PDAs in extremely preterm babies with ibuprofen within 72 hours of birth reduces the incidence of death at 36 weeks postmenstrual age, or moderate or severe bronchopulmonary dysplasia (BPD) at 36 weeks postmenstrual age.
**Primary Endpoints:**	Composite outcome of incidence of death at 36 weeks postmenstrual age, or moderate or severe BPD at 36 weeks postmenstrual age.
**Secondary Objectives:**	To determine if the selective treatment of confirmed large PDAs in extremely preterm babies with ibuprofen within 72 hours of birth results in:<br>• A reduction in the components of the primary outcome, the duration of ventilation, acute morbidities including necrotising enterocolitis (Bell stage 2 or 3), severe intraventricular haemorrhage (grade 3 or 4), cystic periventricular leukomalacia, retinopathy of prematurity (requiring treatment), failure of PDA closure requiring rescue treatment, side effects of drug treatment, gastrointestinal bleeding and the duration of intensive care (short-term secondary objectives).<br>• Improved health outcomes at 2 years corrected age including survival without moderate or severe neurodevelopmental disability (long-term primary objective) and survival without respiratory morbidity (long-

	term secondary objective).<br>An economic evaluation will be carried out from the perspective of the health service. It will take the form of a cost-effectiveness analysis presented in terms of cost per major outcome averted (MOA). The major outcomes are those of the primary outcome, namely death and any moderate or severe BPD at 36 weeks postmenstrual age. Additional analyses will take place on a range of secondary outcomes and on neurodevelopmental outcomes at 2 years. The incremental cost estimate for statistically significant differences in the pre-specified outcomes in primary and subgroup analyses would be computed.
**Secondary Endpoints:**	**Short Term Outcomes**<br>• Death at 36 weeks postmenstrual age<br>• Moderate or severe BPD at 36 weeks postmenstrual age<br>• Severity of BPD at 36 weeks postmenstrual age (see table in Section 6.5)<br>Incidence or duration of the following up to discharge:<br>• Severe intraventricular haemorrhage (IVH) (grade 3/4 with ventricular dilation or intraparenchymal bleeding)<br>• Cystic periventricular leukomalacia (PVL)<br>• Retinopathy of prematurity (ROP) requiring treatment<br>• Significant pulmonary haemorrhage (fresh blood in ET tube with increase in respiratory support)<br>• Pulmonary hypertension requiring treatment with pulmonary vasodilator<br>• NEC definitive and/or complicated (Bell stage II and above) confirmed by radiography and / or histopathology<br>• NEC requiring surgery<br>• Gastrointestinal bleeding within 7 days of the first dose of trial drug administration<br>• Spontaneous intestinal perforation<br>• Closed or non-significant PDA (<1.5 mm) at 3 weeks of age, confirmed by ECHO (or hospital discharge from recruiting centre, if discharged sooner)<br>• PDA $\geq$ 1.5 mm at 3 weeks, not treated medically or by surgical closure<br>• Medical rescue treatment of a symptomatic PDA with a COX

	inhibitor<br>• Rescue treatment of a symptomatic PDA by surgical treatment<br>• Administration and duration of inotropic support<br>• Total duration of respiratory support<br>a) Invasive Ventilation through an endotracheal tube<br>b) Non-invasive support through nasal CPAP, nasal ventilation, or high flow oxygen therapy<br>• Discharge home on oxygen<br>• Duration of initial hospitalisation (birth to discharge home)<br>• Postnatal steroid use for chronic lung disease<br>• Tolerance of ibuprofen treatment within the safety reporting range described in the protocol (Section 9)<br>**Long Term Outcomes**<br>Secondary long term clinical outcomes assessed at 2 years of age corrected for prematurity:<br>• Survival<br>• Survival without moderate or severe neurodevelopmental disability<br>• Individual components of survival without moderate or severe neurodevelopmental disability (in the four domains of motor, cognitive, hearing and visual function). Cognitive disability will be assessed by determining the Parent Report Composite score obtained through the Parent Report of Cognitive Abilities-Revised (PARCA-R) assessment. The PARCA-R assessment will be adapted to include questions to assess hearing and visual function. Motor function will be assessed using the Gross Motor Function Classification System.<br>• Respiratory morbidity. Respiratory morbidity will be assessed by the need for oxygen or respiratory support; presence of persistent cough and/or wheeze; need for regular treatment for respiratory illness; unscheduled attendances at hospital/GP; number of re-hospitalisation episodes and duration.<br>A cost-effectiveness analysis will be conducted of deaths and BPD events avoided and national health services used up to 2 years of age corrected for prematurity.
**Process Outcomes**	Process outcomes will be the following;<br>• Number of doses of trial medication received

	• Adherence to protocol (e.g. protocol violations, incidence of non-symptomatic rescue treatment etc.)<br>• Study withdrawals
**Investigational Medicinal Product:**	Ibuprofen will be provided as a clear sterile preservative-free solution for intravenous injection. An initial dose of 10 mg/kg will be followed by two doses of 5 mg/kg at 24 and 48 hours after the initial dose. The solution of ibuprofen is provided at a concentration of 10 mg/ml in a single-use vial, thus 1 ml/kg, followed by two administrations of 0.5 ml/kg will be required.<br>Placebo will be provided as a clear sterile solution of 0.9% normal saline. The solution will be indistinguishable from that of ibuprofen. It will be given as a 1 ml/kg infusion followed by two infusions of 0.5 ml/kg at 24 and 48 hours.<br>Doses to be calculated on birth weight and administered as a short infusion over 15–30 minutes, preferably undiluted and first dose administered soon after randomisation and within 72 hours of birth.<br>Rescue treatment will be permitted if defined clinical and echocardiography criteria are met.

2. Trial Flow Diagram

Inclusion criteria

- Gestation of 23^{+0} to 28^{+6} weeks
- <72 hours old
- Echocardiogram confirmation of a large PDA
  - dimension of ≥1.5 mm
  - unrestricted pulsatile left to right flow
- The responsible clinician is uncertain about whether the baby might benefit from treatment to close the PDA
- Written informed consent has been obtained from the parent(s)

Exclusion criteria

- No realistic prospect of survival
- Severe congenital anomaly
- Clinical or echocardiography suspicion of congenital structural heart disease that contraindicates treatment with ibuprofen
- Contraindication to use of ibuprofen
- Indomethacin, ibuprofen, or paracetamol administration after birth

Randomisation (1:1 ratio)

Web-based randomisation hosted by NPEU Clinical Trials Unit

Active treatment

Ibuprofen

10, 5, 5 mg/kg each 24 hours apart
(1.0, 0.5, 0.5 ml/kg of 10 mg/ml solution)

OR

Placebo

0.9% Normal saline

1.0, 0.5, 0.5 ml/kg each 24 hours apart

Primary Outcome

Death at 36 weeks PMA
or moderate or severe bronchopulmonary dysplasia (BPD) at 36 weeks PMA

Secondary Short Term Outcomes

- Death at 36 weeks PMA
- Moderate or severe BPD at 36 weeks PMA
- Severity of BPD at 36 weeks PMA
- Other secondary outcomes (see protocol) up to discharge from neonatal unit

Cost Effectiveness Analysis

At discharge from neonatal unit and at 2 years of age corrected for prematurity:

- Deaths and BPD events avoided
- NHS services used up to 2 years of age corrected for prematurity

Secondary Long Term Outcomes (at 2 years of age corrected for prematurity)

- Survival
- Survival without moderate or severe neurodevelopmental disability
- Individual components of survival without moderate or severe disability (in the four domains of motor, cognitive, hearing and visual function)
- Survival without respiratory morbidity

3. Abbreviations

**ADR**	Adverse Drug Reaction
**AE**	Adverse Event
**AR**	Adverse Reaction
**ARR**	Absolute Risk Reduction
**BPD**	Bronchopulmonary Dysplasia
**CI**	Chief Investigator
**CIG**	Co-Investigator Group
**COX**	Cyclo-oxygenase
**CPAP**	Continuous Positive Airway Pressure
**DA**	Ductus Arteriosus
**DCF**	Data Collection Form
**DMC**	Data Monitoring Committee
**DSUR**	Development Safety Update Report
**GCP**	Good Clinical Practice
**GMFCS**	Gross Motor Function Classification System
**GP**	General Practitioner
**HSCIC**	Health and Social Care Information Centre
**HTA**	Health Technology Assessment
**ICF**	Informed Consent Form
**ICH**	International Conference on Harmonisation
**IMP**	Investigational Medicinal Product
**IRAS**	Integrated Research Application System
**ITT**	Intention to Treat
**IVH**	Intraventricular Haemorrhage
**LCRN**	Local Clinical Research Network
**LRN**	Local Research Nurse
**MCRN**	Medicines for Children Research Network
**MHRA**	Medicines and Healthcare products Regulatory Agency
**nCPAP**	Nasal Continuous Positive Airway Pressure
**NEC**	Necrotising Enterocolitis
**NHS**	National Health Service
**NIHR**	National Institute for Health Research
**NPEU CTU**	National Perinatal Epidemiology Unit Clinical Trials Unit

**NSAID**	Non-Steroidal Anti-inflammatory Drug
**OR**	Odds ratio
**PARCA-R**	Parent Report of Cognitive Abilities-Revised
**PDA**	Patent Ductus Arteriosus
**PI**	Principal Investigator
**PIL**	Parent Information Leaflet
**PMA**	Postmenstrual Age
**PMG**	Project Management Group
**PVL**	Cystic Periventricular leukomalacia
**R&D**	NHS Trust Research and Development Department
**REC**	Research Ethics Committee
**RDS**	Respiratory Distress Syndrome
**ROP**	Retinopathy of Prematurity
**SAE**	Serious Adverse Event
**SAR**	Serious Adverse Reaction
**SUSAR**	Suspected Unexpected Serious Adverse Reaction
**TSC**	Trial Steering Committee

4. Introduction

4.1. Background and Rationale

The Ductus Arteriosus (DA) is a vessel that allows blood from the right ventricle to bypass the fetal lungs to the placenta. In term babies it closes spontaneously after birth when breathing is established and is structurally closed after a few days. However, in a large number of preterm babies, the vessel does not close spontaneously resulting in a condition known as Patent Ductus Arteriosus (PDA). Around 7,000 extremely preterm babies (<29 weeks of gestation) are born in the UK every year. In 40% the PDA will fail to close spontaneously even by 4 months of age. [Nemerofsky et al, 2008].

PDA is associated with a number of serious and life-threatening short and long term complications including low blood pressure (hypotension), bleeding in the lungs (pulmonary haemorrhage) and brain (intraventricular haemorrhage (IVH)), systemic complications such as necrotising enterocolitis (NEC), bronchopulmonary dysplasia (BPD), and long term health problems such as neurodevelopmental disability and chronic respiratory problems. The persistence of PDA is associated with an 8-fold rise in neonatal mortality [Noori S et al, 2009]. In addition, as PDA is very common in extreme preterm babies and is associated with a prolonged need for respiratory support and hospitalisation, it places a significant financial burden on the National Health Service (NHS).

Historically, clinicians who have been concerned about the complications associated with a PDA have attempted to close PDAs utilising medical (pharmacological) or surgical treatment. Traditionally, medical treatment is instituted as prophylactic treatment (within 24 hours of birth) or symptomatic treatment (usually 5−7 days after birth). Prophylactic pharmacological treatment of all preterm babies unnecessarily exposes a large proportion of babies to the potentially serious side effects of drug treatment, when their PDA would have closed spontaneously. Symptomatic treatment on the contrary delays treatment while waiting for symptoms to appear and could result in a loss of treatment benefit as irreversible damage may have already been done.

Moreover, the practice of a conservative approach of not treating, seems to originate from uncertainty regarding the management of PDA rather than evidence favouring no intervention. This is due to the fact that most studies conducted to date have involved more mature preterm babies (over 1,000 g or 28 weeks of gestation) whose PDA is more likely to close spontaneously. The studies were also largely designed to assess PDA closure rates rather than clinically important outcomes.

It is now suggested that large PDAs (those with a diameter of ≥1.5 mm) through which blood flow is pulsatile and unrestricted are less likely to close spontaneously. Targeted early

treatment of large PDAs whilst asymptomatic has the potential to overcome the disadvantages of both the prophylactic and symptomatic approaches. Although clinical detection of PDA whilst asymptomatic is challenging, it can be assessed using bedside echocardiography.

Non-steroidal anti-inflammatory drugs, especially indomethacin and ibuprofen have been widely used for the treatment of PDA. Short term efficacy of indomethacin and ibuprofen are equivalent in the treatment of PDA [Su BH et al, 2008]. Ibuprofen however appears to reduce the risk of NEC and is associated with fewer clinical gastrointestinal and renal side effects compared to indomethacin, hence it is the drug of choice for this trial. Paracetamol, has also been recently reported in case studies for closure of symptomatic PDA but further research needs to be done to establish its effectiveness [Oncel MY et al, 2013].

The aim of this trial is to examine whether the pharmacological closure of a large PDA (identified by echocardiography) in extremely preterm babies whilst asymptomatic has a clinically important impact on both short and long term health and economic outcomes.

4.2. Current Evidence Supporting Trial Rationale

Although the number of extremely preterm babies that survive has increased due to improvements in neonatal care, the proportion of babies with moderate or severe disability has remained largely unchanged. Concern regarding this is reflected in the results of a survey conducted by the Medicines for Children Research Network (MCRN) which identified PDA and BPD as key areas in which clinicians believed further evidence and research is most needed.

To date, the majority of studies of prophylactic or symptomatic treatment of PDA have included babies up to 34 weeks of gestation, have been small in size, were designed to assess PDA closure rates rather than short or long term clinical outcomes and are now relatively old, all of which limit the ability to draw meaningful conclusions from the results. Furthermore, there are no recent trials reporting outcomes after selective early treatment of PDA based both on duct size and haemodynamic assessment. Thus the current literature falls short of providing substantive evidence on the management of PDA among extreme preterm babies leading to uncertainty and heterogeneity in clinical practices.

A recent cohort trial identified presence of a large PDA (defined as a PDA dimension of ≥1.5 mm) on day 3 in babies born before 28 weeks of gestation with threefold increase in odds of death or severe morbidity compared with neonates without PDA (Odds Ratio (OR) 3.4; 95% Confidence Interval (CI) 1.1 to 11.0). Neonates with a large PDA were also reported to have increased odds of IVH (OR 4.2; 95% CI 1.3 to 14.0) and BPD (OR 3.7; 95% CI 1.0 to 14.0) compared with neonates with no PDA [Sellmer A et al, 2013]. In preclinical trials,

pharmacologic PDA closure is reported to improve alveolarisation and minimise the impaired postnatal alveolar development that is the pathologic hallmark of "new bronchopulmonary dysplasia (BPD)" [Clyman RI, 2013]. An early selective treatment approach for closure of a PDA is suggested to trial its effect on BPD, which is the hypothesis of this trial.

Both indomethacin and ibuprofen have been shown to have comparable efficacy in closing PDA. The relative risks of treatment strategies adapted from the Cochrane Collaboration reviews are outlined in this table [Fowlie PW, 2010; Ohlsson A, 2011; Ohlsson A, 2010; Cooke I, 2009].

		Relative risk (95% Confidence interval)						^^
Author	Intervention	Symptomatic PDA	Death before 36 Weeks	BPD at 36 weeks	NEC	Severe IVH	Death / Severe Disability 18–24 Months	Duration of Ventilation (days)
Fowlie PW, *Cochrane Review* 2010, comparison 1	Prophylactic indomethacin	0.44<br>(0.38, 0.50)*	0.82<br>(0.65, 1.03)	1.06<br>(0.92, 1.22)	1.09<br>(0.82, 1.46)	0.66<br>(0.53, 0.82)*	1.02<br>(0.90, 1.15)	-1.83<br>(-5.53, 1.87)
Ohlsson A, *Cochrane Review* 2011, comparison 1	Prophylactic ibuprofen	0.17<br>(0.11, 0.26)*	0.90<br>(0.62, 1.30)	1.04<br>(0.87, 1.25)	1.04<br>(0.63, 1.70)	0.82<br>(0.54, 1.26)	-	1.02<br>(-1.99, 4.03)
Ohlsson A, *Cochrane Review* 2010, comparison 2	Symptomatic PDA (indomethacin vs. ibuprofen)	1.28<br>(0.48, 3.38)	1.12<br>(0.59, 2.11)	1.12<br>(0.77, 1.61)	0.68<br>(0.47, 0.99)*	1.21<br>(0.74, 1.98)	-	-1.96<br>(-4.97, 1.05)
Cooke L, *Cochrane Review* 2009, comparison 1	Early asymptomatic indomethacin	0.36<br>(0.19, 0.68)*	1.32<br>(0.45, 3.86)	0.91<br>(0.62, 1.35)	0.41<br>(0.05, 3.68)	-	-	-5.00<br>(-17.33, 3.34)
Ohlsson A, *Cochrane Review* 2010, comparison 1	Early asymptomatic ibuprofen	0.27<br>(0.12, 0.60)*	0.8<br>(0.34, 1.90)	0.99<br>(0.88, 1.11)	1.00<br>(0.64, 1.55)	1.00<br>(0.47, 2.15)	-	-

*p<0.05
^^ Weighted Mean Differences [WMD] (95% Confidence interval)

5. Trial Objective

5.1. Primary Objective

To determine if selective early treatment of echocardiographically confirmed large PDAs in extremely preterm babies with ibuprofen within 72 hours of birth reduces the incidence of death at 36 weeks postmenstrual age or moderate or severe bronchopulmonary dysplasia (BPD) at 36 weeks postmenstrual age.

5.2. Secondary Objectives

To determine if the selective treatment of confirmed large PDAs in extremely preterm babies with ibuprofen within 72 hours of birth results in:

- A reduction in the components of the primary outcome, the duration of ventilation, acute morbidities including necrotising enterocolitis (Bell stage 2 or 3), severe intraventricular haemorrhage (grade 3 or 4), cystic periventricular leukomalacia, retinopathy of prematurity (requiring treatment), failure of PDA closure requiring rescue treatment, side effects of drug treatment, gastrointestinal bleeding and the duration of intensive care (short-term secondary objectives).

- Improved health outcomes at 2 years corrected age including survival without moderate or severe neurodevelopmental disability (long-term primary objective) and survival without respiratory morbidity (long-term secondary objective).

An economic evaluation: an economic evaluation will be carried out from the perspective of the health service. It will take the form of a cost-effectiveness analysis presented in terms of cost per major outcome averted (MOA). The major outcomes are those of the primary outcome, namely death and moderate or severe BPD at 36 weeks. Additional analyses will take place on a range of secondary outcomes and on neurodevelopmental outcomes at 2 years. The incremental cost estimate for statistically significant differences in the pre-specified outcomes in primary and subgroup analyses would be computed.

6. Trial Design

6.1. Summary

This is a multicentre, masked, randomised, placebo-controlled parallel group trial to determine if the treatment of a large PDA with ibuprofen in extremely preterm babies (23^{+0} to 28^{+6} weeks of gestation) improves short and long term health and economic outcomes. The main trial will be preceded by an internal pilot phase which will be used to assess the suitability of trial procedures and likelihood of recruitment targets being achieved.

The entire trial is anticipated to take 82 months to complete and aims to recruit a total of approximately 730 extremely preterm babies.

6.2. Inclusion Criteria

Babies will be considered eligible for inclusion into the trial if they are:

- Born at 23^{+0} to 28^{+6} weeks of gestation
- Less than 72 hours old
- Confirmed by echocardiography as having a large PDA which
  - is at least 1.5 mm in diameter (determined by gain optimised colour Doppler)

  and

  - has unrestrictive pulsatile left to right flow (ratio of flow velocity in PDA Maximum (V_{max}) to Minimum (V_{min}) > 2:1)

In addition:

- The responsible clinician is uncertain about whether the baby might benefit from treatment to close the PDA
- Written informed consent has been obtained from the parent(s)

6.3. Exclusion Criteria

Babies will be excluded from participation in the trial if they have:

- No realistic prospect of survival
- Severe congenital anomaly
- Clinical or echocardiography suspicion of congenital structural heart disease that contraindicates treatment with ibuprofen
- Other conditions that would contraindicate the use of ibuprofen (clinically significantly intracranial or gastrointestinal haemorrhage, coagulopathy, thrombocytopenia (platelet count <50,000), renal failure, pulmonary hypertension, known or suspected necrotising enterocolitis (NEC))
- Indomethacin, ibuprofen, or paracetamol administration after birth

6.4. Setting

The trial will be conducted in 25 level 3 neonatal units across the UK (4 units will be involved in the internal pilot phase).

Only units that are in equipoise in the way that they manage PDA, are able and agree to perform echocardiograms within 72 hours of birth to confirm the presence of a large PDA and are part of a Local Clinical Research Network (LCRN) will be selected.

6.5. Primary Outcome

The primary outcome is defined as a composite outcome of death at 36 weeks postmenstrual age, or moderate or severe BPD at 36 weeks postmenstrual age.

**TABLE:** Severity-Based Diagnostic Criteria for BPD	
Time point of assessment:	36 weeks PMA
Therapy with oxygen > 21% and/or respiratory support for ≥ 28 days and the following:	
Mild BPD;	Baby is breathing room air
Moderate BPD;	Baby is in 22 - 29% oxygen, or 0.01 – 1.0 l/min
Severe BPD;	FiO_2 ≥ 0.3, or low flow oxygen ≥ 1.1 l/min, or the baby is receiving any respiratory support (ventilation, CPAP, or high flow oxygen therapy) to achieve saturations of ≥ 91%

The need for oxygen is subjective and hence oxygen dependency will be confirmed using an 'oxygen reduction test'. This is based on the threshold at which the baby is able to maintain oxygen saturations ≥ 91% whilst breathing in air or at a given minimum FiO_2. Babies unable to achieve this will be considered to be oxygen dependent. This test will only apply to those babies whose oxygen requirements are < 0.3, or low flow oxygen < 1.1 l/min, and who have not received any additional respiratory support in the previous 24 hours. Babies outside of this will not be tested, but their oxygen requirements will be captured on the relevant data collection form.

6.5.1. Oxygen Reduction Test

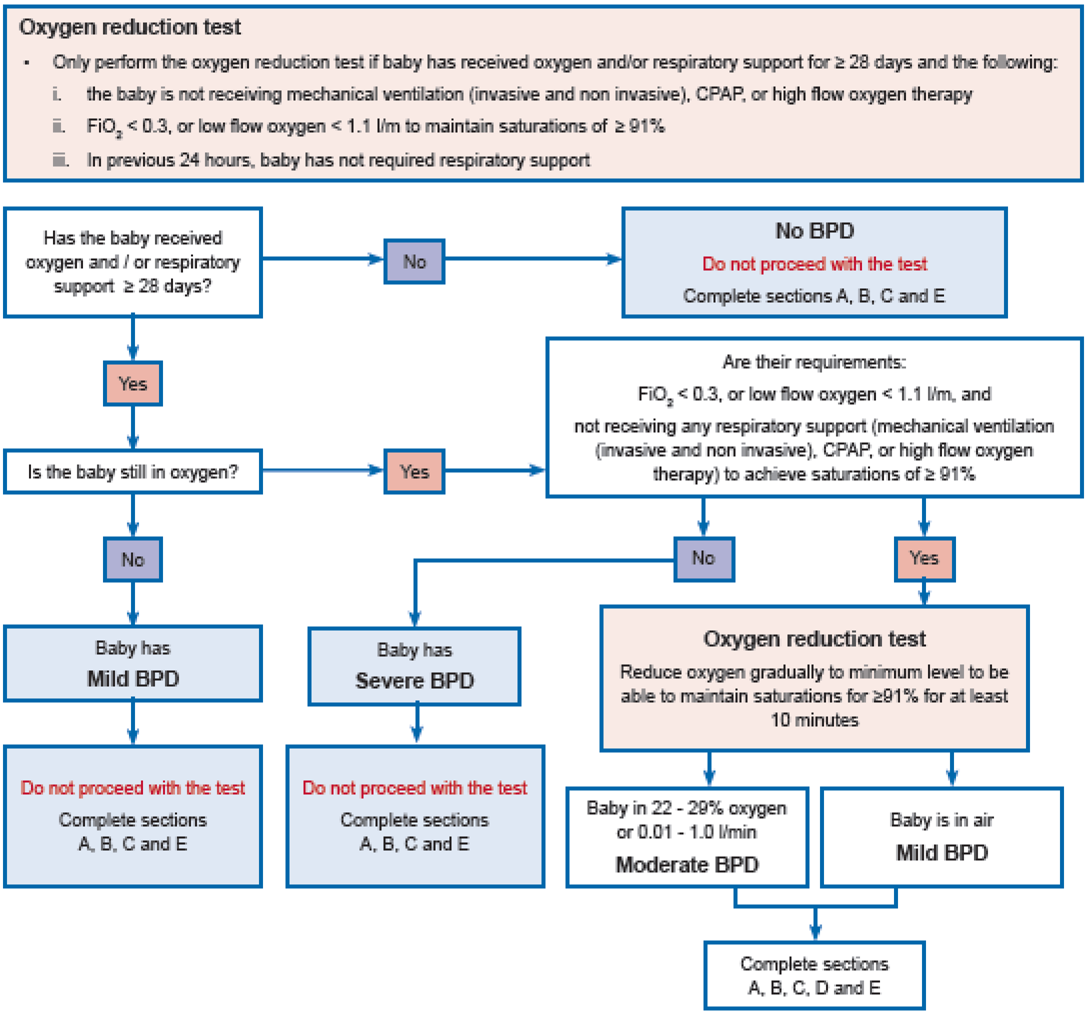

6.6. Secondary Outcomes

Secondary outcomes are divided into short and long term outcomes.

Short term outcomes

- Death at 36 weeks postmenstrual age
- Moderate or severe BPD at 36 weeks postmenstrual age
- Severity of BPD at 36 weeks postmenstrual age (see table in Section 6.5)

Incidence or duration of the following up to discharge:

- Severe intraventricular haemorrhage (IVH) (grade 3/4 with ventricular dilation or intraparenchymal bleeding)
- Cystic periventricular leukomalacia (PVL)
- Retinopathy of prematurity (ROP) requiring treatment
- Significant pulmonary haemorrhage (fresh blood in ET tube with increase in respiratory support)
- Pulmonary hypertension requiring treatment with pulmonary vasodilator

- NEC definitive and/or complicated (Bell stage II and above) confirmed by radiology and / or histopathology
- NEC requiring surgery
- Gastrointestinal bleeding within 7 days of the first dose of trial drug administration
- Spontaneous intestinal perforation
- Closed or non-significant PDA (<1.5 mm) at 3 weeks of age, confirmed by ECHO (or hospital discharge from recruiting centre, if discharged sooner)
- PDA $\geq$ 1.5 mm at 3 weeks, not treated medically or by surgical closure
- Medical rescue treatment of a symptomatic PDA with a COX inhibitor
- Rescue treatment of a symptomatic PDA by surgical treatment
- Administration and duration of inotropic support
- Total duration of respiratory support
  a) Invasive ventilation through an endotracheal tube
  b) Non-invasive support through nasal CPAP, nasal ventilation, or high flow oxygen therapy
- Discharge home on oxygen
- Duration of initial hospitalisation (birth to discharge home)
- Postnatal steroid use for chronic lung disease
- Tolerance of ibuprofen treatment within the safety reporting range described in the protocol (Section 9)

Long Term Outcomes

Secondary long term clinical outcomes assessed at 2 years of age corrected for prematurity:

- Survival
- Survival without moderate or severe neurodevelopmental disability
- Individual components of survival without moderate or severe neurodevelopmental disability (in the four domains of motor, cognitive, hearing and visual function). Cognitive disability will be assessed by determining the Parent Report Composite score obtained through the Parent Report of Cognitive Abilities-Revised (PARCA-R) assessment. The PARCA-R assessment will be adapted to include questions to assess hearing and visual function. Motor function will be assessed using the Gross Motor Function Classification System.
- Respiratory morbidity. Respiratory morbidity will be assessed by the need for oxygen or respiratory support; presence of persistent cough and/or wheeze; need for regular treatment for respiratory illness; unscheduled attendances at hospital/GP; number of re-hospitalisation episodes and duration.

A cost-effectiveness analysis will be conducted of deaths and BPD events avoided and national health services used up to 2 years of age corrected for prematurity.

6.7. Process Outcomes

Process Outcomes will be the following;

- Number of doses of trial medication received
- Adherence to protocol (e.g. protocol violations, incidence of non-symptomatic rescue treatment etc.)
- Study withdrawals

7. Trial Procedures

7.1. Trial Assessments

Procedure	Baby Hospitalisation						Infant 2 Years Corrected Age[6,7, 8]
	Screening [1]	Trial Entry and Treatment (days 1–3)	Up to 7 days after trial medication	3 weeks of Age	36 weeks PMA	Discharge	
Demography[10]		✓				✓	✓
Echocardiogram/Colour Doppler[9]	✓			✓			
Confirmation of Eligibility	✓						
Consent		✓					
Randomisation[2]		✓					
Ibuprofen/Placebo Dosing[3]		✓					
IVH / PVL ultrasound scans			✓		✓		
NEC						✓	
Oxygen Reduction Test					✓		
SAEs[4]		✓	✓				
Concomitant Medication[5]	✓	✓		✓	✓	✓	✓
PARCA-R Questionnaire Assessment[6]							✓
Visual Assessment[6]							✓
Hearing Assessment[6]							✓
Motor Assessment[7]							✓
Respiratory Assessment[8]							✓

1 Screening assessments to be completed sufficiently in advance to enable randomisation and dosing within 72 hours of birth.

2 Randomisation to be completed sufficiently in advance to enable dosing within 72 hours of birth.

3 Initial trial drug administrations to be given soon after randomisation and within 72 hours of birth. Subsequent doses to be administered 24 hours after the initial dose.

4 Only adverse events which are serious will be recorded from first dose until 7 days after trial medication. Only unforeseeable SAEs will be reported.

5 Concomitant medications to be recorded only in relation to unforeseeable SAEs. In the event of an

unforeseeable SAE all concomitant medication, including medication given to the baby's mother, 7 days prior to the onset of the event to the time of its resolution must be recorded on the SAE form.

6 Cognitive, visual and hearing function will be assessed using the PARCA-R questionnaire, expanded to include questions to assess visual and hearing function.

7 Motor function will be assessed using the Gross Motor Function Classification System (GMFCS).

8 Respiratory assessments will be performed using a separate validated questionnaire. There will be no requirement for the infants to be assessed for respiratory and / or other neurodevelopmental functions by medically qualified personnel.

9 An echocardiogram scan will be performed when the baby reaches 3 weeks of age or at hospital discharge if discharged earlier.

10 Demography and medications will be assessed through the PARCA-R and other questionnaires.

7.2. Structure and Duration of the Trial

The total duration of recruitment into this trial will consist of an internal pilot phase (9 month recruitment period) and main trial (36 month recruitment period). The aim is to recruit approximately 730 babies from participating neonatal units across the UK in about 45 months (internal pilot and main trial).

For the main trial, the recruitment period is based on an approximate yearly admittance of 2,000 babies born between 23 and 28 weeks of gestation to the 25 participating level 3 neonatal units and the assumption that about 46% of these babies will have a large PDA [Stoll et al, 2010]. This would result in approximately 900 babies being eligible for inclusion in this trial. Assuming a conservative uptake rate of 20−30% this would equate to around 250 babies being enrolled per year (1 per unit per month).

The trial will consist of an internal pilot phase, run over a period of 13 months (including a 4 month trial set up period), in four level 3 neonatal units to test whether the current trial design and associated procedures will allow overall recruitment targets to be achieved. Projections suggest that around 30 babies should be recruited in that time. Data collected from the internal pilot phase of the trial will be included in the final analysis.

The decision to progress to the main trial using the current design will be made in consultation with the Trial Steering Committee (TSC) and funder. Stop/go criteria upon which a decision will be made will be established prior to the start of the internal pilot phase. Should a decision be made not to progress to the main phase, a report on the internal pilot phase will be submitted for publication according to the publication policy.

All enrolled babies will be followed up at 2 years of age corrected for prematurity. Thus the duration of trial participation will be up to 28 months (2 years corrected age). Where we have been unable to contact families within this timeframe we will attempt to collect information about infants in the trial until the end of the funding. Further longer term follow-up at primary school age may be considered but will require separate funding. This may be undertaken as an amendment to this trial or as a separate application depending on the circumstances at the time.

7.3. Initial Eligibility Assessment

Extremely preterm babies potentially suitable for the trial will be identified by the healthcare team within the neonatal unit. Babies however will only be considered eligible for enrolment into the trial and their parent(s) approached for consent after they have undergone an echocardiogram and Doppler assessment and have been confirmed to have a large PDA.

The initial echocardiogram and Doppler assessment will incorporate:

- Size of the PDA and flow pattern according to standard trial methodology
- Size of the PDA will be determined at the site of maximum constriction (minimum diameter) using gain optimisation typically at the pulmonary end by determining the average of 3 separate clips
- If the size of the PDA is at least 1.5 mm, flow pattern will be determined by placing the pulse gate in the PDA while adjusting the velocity scale to its highest setting. If the shunt direction is >1/3 duration of a cycle being right to left, then a rescan will be attempted after a few hours.

If the echocardiogram findings raise concerns about possibility or diagnosis of congenital heart disease, a referral will be made to a paediatric cardiologist as per clinician discretion.

7.4. Informed Consent

Written informed consent will be sought from parent(s) of potentially eligible babies only after the baby has been confirmed to have a large PDA and the baby's parent(s) have been given a full verbal and written (via the Parent Information Leaflet (PIL)) explanation of the trial. Parent(s) who do not speak English will only be approached if an adult interpreter is available. Relatives may not interpret.

Written informed parental consent will be obtained by means of a dated parental signature and the signature of the person who obtained informed consent; this will be the Principal Investigator (PI) or appropriately qualified healthcare professional who has been delegated authority. A copy of the signed informed consent form (ICF) will be given to the parent(s). Further copies with be retained in the baby's medical notes and by the PI. The original signed consent form will be sent to the National Perinatal Epidemiology Unit Clinical Trials Unit (NPEU CTU).

7.5. Randomisation

Treatment allocation of ibuprofen or placebo will be in a ratio of 1:1 and masked such that the allocation will not be known by clinicians, the baby's family or the trial outcome assessors.

Randomisation will be managed via a secure web-based randomisation facility hosted by the NPEU CTU with telephone back-up available at all times (24/7, 365 days a year). The randomisation program will use a minimisation algorithm to ensure balance between the groups with respect to the size of the PDA, gestational age at birth, age at randomisation, sex, trial site, multiple births, mode of respiratory support at randomisation (1) invasive ventilation (by an endotracheal tube), or (2) non-invasive respiratory support (nasal CPAP, nasal ventilation, or high flow oxygen therapy - humidified high flow nasal cannula), or (3) receiving no mechanical or pressure support (in room air or low flow or ambient oxygen) and receiving inotropes or not at the time of randomisation. Babies of multiple births will be randomised individually.

The Senior Trials Programmer at the NPEU CTU will write the randomisation program and hold the treatment allocation codes. If necessary, the code may be broken for a single baby at the request of the site PI or clinician in charge of the baby. See Section 8.6 for the procedure for unmasking treatment allocation.

7.6. Echocardiograms

Echocardiograms are performed as part of the normal care of preterm babies. However, clinicians will be required to perform an echocardiogram within 72 hours of birth, at 3 weeks of age or at discharge from the neonatal unit if discharged before this time.

Echocardiogram scans will be reviewed by a qualified clinician, who is not involved in recruiting for the trial, to assess consistency between clinicians. All babies recruited to the internal pilot phase and a randomly selected sample from the main trial, equating to 10% of echocardiogram scans used to confirm trial eligibility, will be reviewed. Principal Investigators will be informed of the review findings.

Training will be provided during the trial to minimise any variations in practice. Any difference in measurements between the site investigator and reviewer will be documented to aid with on-going training. Details of both the echocardiogram procedures to be followed and the process for submitting scans for independent review will be described in a separate handbook.

7.7. Concomitant Medications

Concomitant medication given to a baby will be recorded in the event that an unforeseeable serious adverse event is reported for that baby. If such an event is reported, all concomitant medication given 7 days prior to onset of the event, including medication given to the baby's mother if 7 days is prior to the birth of the baby, up to its resolution will be detailed on the SAE form provided for the trial.

7.8. Permitted and Non-Permitted Medications

All prescribed medications deemed necessary to provide adequate supportive care to the baby, are permitted at any stage during the trial period. However, open treatment with indomethacin or ibuprofen or other non-steroidal anti-inflammatory drugs (NSAIDs) should be avoided unless the criteria for rescue treatment (defined in Section 8.4) are met.

As a NSAID, ibuprofen may interact with the following medicinal products:

- Diuretics – ibuprofen may reduce the effect of diuretics; diuretics can increase the risk of nephrotoxicity of NSAIDs in dehydrated patients
- Anticoagulants – ibuprofen may increase the effect of anticoagulants and enhance the risk of bleeding
- Corticosteroids – ibuprofen may increase the risk of gastrointestinal bleeding
- Nitric oxide – since ibuprofen also inhibits platelet function, combining the drugs may in theory increase the risk of bleeding
- NSAIDs – the concomitant use of more than one NSAID should be avoided because of the increased risk of adverse reactions

The concomitant administration of other medication is not restricted but should be closely monitored for an interaction by the treating clinician.

7.8.1. Supportive Care of Enrolled Babies

The management of babies including ventilator management and fluid therapy during intensive or high dependency care will be guided by the European Consensus Guidelines for Management of Respiratory Distress Syndrome (RDS) in preterm babies. An effort will be made to minimise the differences in treatment practices between sites through training on the guidelines.

7.9. Stopping Trial Interventions

The intervention may have to be (temporarily) stopped if the baby develops any adverse effects necessitating stoppage. For example, if anuria, marked oliguria (<0.6 ml/kg/hr), or clinically significant bleeding is evident at the scheduled time of the second or third dose, no additional dosage should be given until laboratory results indicate that renal function has returned to normal, or bleeding has stopped. In the event of GI perforation discontinue trial medication.

At all stages it will be made clear to the parent(s) that they remain free to withdraw their baby from the trial at any time without the need to provide any reason or explanation. Parent(s) will be made aware that a decision to withdraw their baby will have no impact on any aspect of their baby's continuing care. If parent(s) choose to withdraw their baby from trial participation, permission will be sought to complete data collection and use data up to the point of withdrawal from the trial.

A baby may also be withdrawn from the trial, if deemed by the Principal Investigator to be in their best interests.

7.10. End of Trial

The end of trial will be defined as the last infant's assessment at 2 years of age corrected for prematurity. An End of Trial Declaration will be made to the Medicines and Healthcare products Regulatory Agency (MHRA) and approving Research Ethics Committee (REC) within 3 months of this date.

7.11. Early Trial Cessation

A decision may be made by the Trial Steering Committee (TSC) to stop the trial early following a recommendation from the Data Monitoring Committee (DMC), on review of interim trial data, or evidence from other relevant studies becoming available. Guidelines for the early cessation of the trial will be agreed with the DMC and documented in the DMC Charter.

7.12. Remuneration

No financial or material incentive or other form of compensation will be given to babies or their parent(s) as a result of taking part in this trial.

8. Investigational Medicinal Product (IMP)

8.1. Dosing and Administration

Ibuprofen will be supplied as a clear sterile preservative-free solution at a concentration of 10 mg/ml in vials. Cartons containing three single use vials will be provided. Each carton will be labelled with a unique code and in compliance with the guidance given in Annexe 13 of the European Commission's guidelines for Good Manufacturing Practice.

An initial loading dose of 10 mg/kg (1 ml/kg) of ibuprofen will be administered, followed by two 5 mg/kg (0.5 ml/kg) doses at 24 and 48 hours after the initial dose. Doses are to be calculated on the birth weight of the baby. Each dose is to be given as a short intravenous infusion over 15−30 minutes. All 3 doses will be given unless there are adverse effects necessitating stoppage, as referenced in Section 7.9. Placebo will be supplied as a clear sterile solution of 0.9% saline solution for injection. Cartons identical to those for ibuprofen, each containing three identical single use vials will be provided. Volume of solution to be administered will be calculated following the calculations for ibuprofen dosing.

Following randomisation, first dose should be administered soon after randomisation and within 72 hours of birth. The recommended storage will be in line with the Full Prescribing Information and once the vial is opened the drug must be used immediately.

8.2. Distribution

Sufficient supplies of IMP will be provided to each site. Distribution and use of IMP will be tracked by the staff at the NPEU, using a 'pack management system' and additional supplies provided as and when needed.

8.3. Accountability

Trial drug packs will be dispensed by pharmacy and stocked on the neonatal units. The dispensing of the trial drug from pharmacy will require a completed prescription form. Detailed accountability records will be maintained to document which pack of medication is dispensed to which baby. Site staff will be required to write the baby's trial number and initials on the trial pack allocated. Part used packs will be kept separate from unused packs.

Pharmacy will maintain an overall inventory of stock received and dispensed.

8.4. Rescue Treatment

If the clinical condition of a baby warrants intervention, rescue treatment can be given to close the PDA (medical or surgical). The following criteria however have been devised to limit and rationalise the use of rescue treatment but it is recognised that clinicians may need to override this guidance in the best interests of the baby. Clinical responsibility for the care of the baby will remain fully with the neonatal clinical team irrespective of the trial.

Rescue treatment (both medical and surgical) is permitted within the protocol if the following minimum criteria are met and other medical management strategies have been tried. Surgical treatment however should only be considered if the PDA remains persistently large after one course of treatment with a COX inhibitor or in circumstances where medical treatment may be contraindicated or time does not permit medical rescue treatment first.

1. Inability to wean on ventilator (ventilated for at least 7 days continuously) and inability to wean oxygen, or
2. Persistent hypotension/pulmonary haemorrhage/signs of cardiac failure

AND

3. Echocardiographic findings of a large PDA (PDA $\geq$ 2.0 mm with pulsatile flow) AND hyperdynamic circulation or ductal steal (refer to Baby-OSCAR ECHO workbook).

All rescue treatment will be administered in an open fashion.

A persistent open PDA requiring open label treatment (medical or surgical) should be reported to the trial co-ordinating centre using Form 5: Rescue Medication Form.

8.5. Masking of Trial Medication

Ibuprofen and placebo will be indistinguishable from each other. To maintain masking, each baby will be issued a unique allocation number that will correspond to a carton number.

8.6. Procedure for Unmasking

In the event of an emergency, a baby's treatment allocation may be unmasked by contacting the NPEU CTU during working hours, or calling an out of hours help line managed by a company called Message Direct who will contact appropriate people. The contact details for both the NPEU CTU and Message Direct are as follows:

9:00 am to 5.00 pm	NPEU CTU:	01865 617 965
5.00 pm to 9.00 am and weekends	Message Direct:	0800 138 5451

Details of contact numbers will also be filed in the Investigator Site File.

Details of the person requesting unmasking and the reason for the request will be recorded.

Wherever possible, the unmasking of a baby's treatment allocation should be discussed with the Chief Investigator or delegate in advance.

9. Safety Reporting

9.1. Definitions

9.1.1. Adverse Event (AE)

An adverse event is any untoward medical occurrence in a participant administered a medicinal product, which does not necessarily have to have a causal relationship with this treatment. An AE can therefore be any unfavourable and unintended sign (including an abnormal laboratory finding), symptom or disease temporally associated with the use of the trial medication, whether or not considered related to the trial medication.

9.1.2. Adverse Reaction (AR)

All untoward and unintended responses to a medicinal product related to any dose. The phrase “response to a medicinal product” means that a causal relationship between trial medication and an AE is at least a reasonable possibility, i.e. the relationship cannot be ruled out.

9.1.3. Serious Adverse Event (SAE)

A serious adverse event is any untoward medical occurrence that:

- Results in death
- Is life-threatening
- Requires participant hospitalisation or prolongation of existing hospitalisation
- Results in persistent or significant disability/incapacity
- Is a congenital anomaly/birth defect
- Is an important medical event

The term ‘severe’ is often used to describe the intensity (severity) of a specific event; the event itself, however, may be of relatively minor medical significance. This is not the same as ‘serious’, which is based on participant/event outcome or action criteria usually associated with events that pose a threat to a participant’s life or functioning.

The term ‘life-threatening’ in the definition of serious refers to an event in which the participant was at risk of death at the time of the event; it does not refer to an event that hypothetically might have caused death if it were more severe.

Medical and scientific judgement should be exercised in deciding whether an adverse event is serious in other situations.

9.1.4. Foreseeable Serious Adverse Events

Foreseeable SAEs are those events which are foreseen in the patient population or as a result of the routine care/treatment of a patient.

The following serious adverse events are a foreseeable occurrence in this population of preterm babies and as such do not require reporting as SAEs:

- Death (unless unforeseeable in this population)
- Respiratory failure
- Pulmonary haemorrhage
- Necrotising enterocolitis
- Clinically significant intracranial abnormality on cranial ultrasound scan – intracranial haemorrhage or white matter injury
- Retinopathy of prematurity
- Hypotension
- Hyperbilirubinemia necessitating exchange transfusion
- Pulmonary hypertension requiring treatment with pulmonary vasodilator
- Spontaneous intestinal perforation
- Impaired renal function (urine output <0.5 mL/kg/hour, and or serum creatinine > 100 μmol/L)
- Anaemia requiring transfusion
- Hypoglycaemia
- Hyperglycaemia
- Haemothorax
- Culture proven sepsis
- Coagulopathy requiring treatment
- Sepsis / ventilator associated pneumonia
- Pneumothorax or air leaks
- Seizures not related to an intracranial event
- Gastrointestinal haemorrhage

9.1.5. Unforeseeable Serious Adverse Events

An unforeseeable SAE is any event that meets the definition of a SAE and is not detailed in the list above as foreseeable. These events should be reported on the trial SAE form provided following the procedures detailed in Section 9.2.1.

9.1.6. Serious Adverse Reaction (SAR)

A serious adverse reaction is a SAE which is considered to have been caused by the administration of trial medication. For a SAE to be considered as a reaction there must be a reasonable probability that it was related to the administration of IMP.

9.1.7. *Suspected Unexpected Serious Adverse Reaction (SUSAR)*

This is a SAR, the nature or severity of which is not consistent with the known safety profile of the trial medication (e.g. Investigator's Brochure for an unapproved IMP or Summary of Product Characteristics for an approved product). The Reference Safety Information for the L-lysine salt of ibuprofen is contained within the Full Prescribing Information which will be used to assess the expectedness of adverse events.

9.1.8. *Causality*

The relationship of each adverse event to the trial medication must be determined by a medically qualified individual according to the following definitions:

Unrelated; where an event is not considered to be related to the IMP;

Possibly; although a relationship to the IMP cannot be completely ruled out, the nature of the event, the underlying disease, concomitant medication or temporal relationship make other explanations possible;

Probably; the temporal relationship and absence of a more likely explanation suggest the event could be related to the IMP;

Definitely; the known effects of the IMP, its therapeutic class or based on challenge testing suggest that the IMP is the most likely cause.

All SAEs as discussed in Section 9.2.1 labelled possibly, probably, or definitely will be considered as related to the IMP.

9.1.9. *Assessment of Safety*

During the course of the trial, safety data will be reviewed by the Data Monitoring Committee (DMC). This will include safety data for SAEs as stated in Section 9.2.1 as well as Section 9.1.4 and 9.1.5. The DMC will, if appropriate, make recommendations regarding continuance of the trial or modification of the trial protocol. The TSC will have ultimate responsibility for deciding whether the trial should be stopped on safety grounds.

9.2. Reporting Procedures

9.2.1. *AE/SAE Reporting*

Causality of adverse events reported in the preterm newborn is difficult to assess since they may be related to the haemodynamic consequences of the patent ductus arteriosus as well as to direct effects of ibuprofen. In addition to this, high incidences of adverse events are foreseeable due to the nature of the patient population and the routine care / treatment. Consequently, only those adverse events identified as serious will be recorded for the trial.

Safety reporting as described in this section for the baby will be monitored from first dose until 7 days after trial medication. Unforeseeable Serious Adverse Events will be reported to the NPEU CTU within 24 hours of staff at the site becoming aware of the event. Details will be recorded on a SAE form (filed in the Investigator Site File) and the form faxed or emailed back to the NPEU CTU. If this is not possible, the unforeseeable SAE may be reported by telephone and the SAE form completed by staff at the NPEU CTU. Follow-up information should be reported on a new SAE form and this forwarded to the NPEU CTU by fax or email.

NPEU will review the report, request any additional information and ensure it is assessed by the CI or his delegate within the reporting timeframe. It will also be reviewed at the next DMC meeting. The CI will inform all Principal Investigators of relevant information that could adversely affect the safety of the participants.

9.2.2. SUSAR Reporting

SUSARs will be reported to the MHRA and the approving Research Ethics Committee (REC) within 7 days if the event resulted in death or was life-threatening and within 15 days for all other SUSARs. In addition, a copy of the SAE form corresponding to the event will be forwarded to the Chair of the DMC. The Chair will also be provided with details of the baby's treatment allocation if requested.

9.2.3. Development Safety Update Report (DSUR)

In addition to the expedited reporting detailed above, the CI will submit a Development Safety Update Report (DSUR) once a year throughout the duration of the trial to the MHRA and REC.

10. Statistics and Analysis

10.1. Sample Size

Evidence from the TIPP trial suggests that the risk of death or BPD in extremely low birth weight babies at 36 weeks postmenstrual age allocated placebo is 52% (95% CI 48% to 56%) [Schmidt et al, 2001]. However this trial investigated the effect of prophylactic treatment and included all babies weighing 500−999g. More recent information using data derived from the latest report of Neonatal Survey Database from the Trent region (2010) provides an approximate rate of death or BPD at 36 weeks postmenstrual age of 53% for all babies admitted to the neonatal unit. These babies would have been treated according to clinical judgement and therefore a proportion of them would have been treated with ibuprofen. Given the risk of death or BPD in babies with a large PDA is inherently higher, it is estimated that the risk in this group is 60%.

Su et al (2008) compared ibuprofen to indomethacin in babies ≤ 28 weeks of gestation having a PDA who were less than 24 hours old. The combined outcome of death within 30 days or BPD at 36 weeks postmenstrual age was observed to be 42% (95% CI 29% to 55%).

It is therefore expected, given that babies will be enrolled up to 72 hours after birth, that the treatment group incidence of death/BPD at 36 weeks will be approximately 48% in the intervention arm. This would imply an absolute risk reduction of 12% (60% to 48%) in the primary outcome of the trial for babies randomised to treatment compared to placebo, which is considered a clinically important difference.

Some babies will require rescue treatment in either the treatment or placebo arm. As rescue treatment should be limited to symptomatic babies meeting only defined criteria, it is considered to have minimal or no effect on the primary outcome. Thus adjustment of the sample size for rescue treatment is not considered necessary.

The following graph depicts a sample size curve for the primary outcome of the trial of death or BPD at 36 weeks postmenstrual age, assuming 90% power, a two-sided 5% significance level and a 60% control group event rate for the primary outcome.

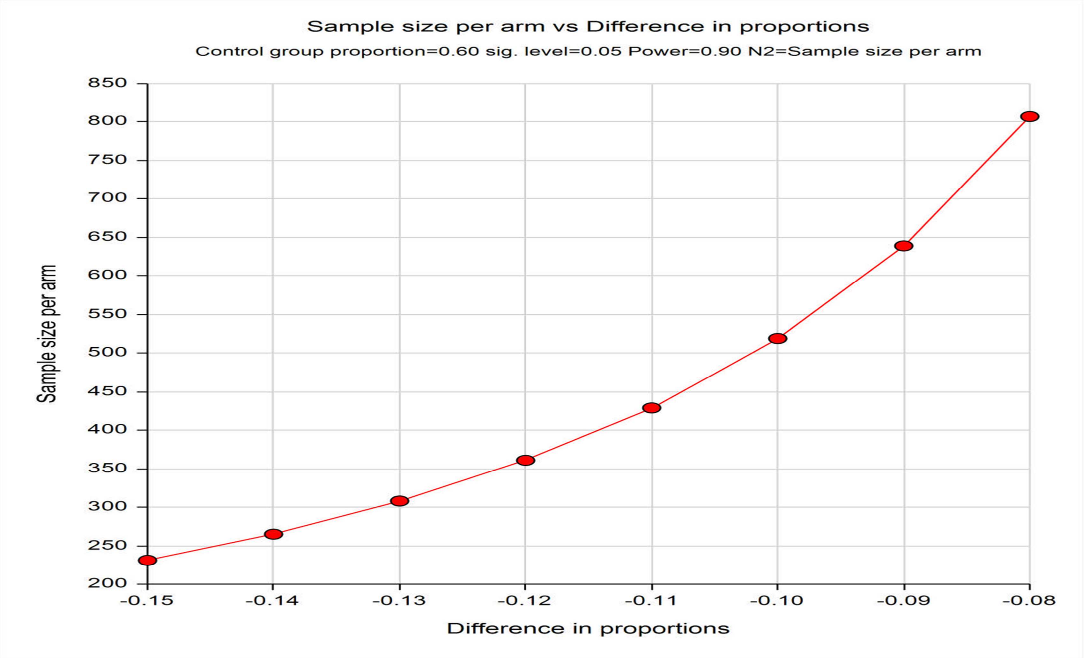

This table summarises this information and allows for 1% loss to follow-up in the primary outcome. Minimal loss to follow-up is expected for the primary outcome since it is a short term outcome and recorded whilst the baby is in hospital.

Control group event rate	Active Rx group event rate	Absolute risk reduction	Relative risk reduction	Approximate total sample size required
60%	47%	13%	22%	620
**60%**	**48%**	**12%**	**20%**	**730**
60%	49%	11%	18%	870

Thus a sample size of approximately 730 babies in total (365 per arm) would be required to detect an absolute risk reduction of 12% (power 90%, 2-sided significance level of 5%) from a control group event rate of 60% to a treatment group event rate of 48%, assuming 1% lost to follow-up.

Regarding outcomes at 2 years corrected age, assuming the risk of a child dying before two years of age is 10%, questionnaires will be sent out to around 660 parents of surviving children. Assuming an attrition rate of 20% reduces the sample size to around 530. The proportion of infants surviving to 2 years without moderate or severe neurodevelopmental disability in the control group is expected to be 55% [Mangham et al, 2009]. With outcome data available on a total sample size of around 600 (including deaths) the trial will have an 80% power to detect an increase in survival without moderate or severe neurodevelopmental disability of 11% from 55% to 66% and 90% to detect an increase of 13% from 55% to 68%.

10.2. Assessment of Outcomes

Short term outcome data will consist of routine clinical and laboratory assessments. The timing and methods for assessing and determining the short outcomes are consistent with those routinely performed for babies in intensive/high dependency care.

Long term outcomes will be assessed using validated parent report questionnaires. Standardised measures (Gross Motor Function Classification System and PARCA-R) and additional questionnaire items designed to elicit key information regarding visual and hearing impairment, respiratory morbidity and health economic outcomes will be combined into a single trial specific questionnaire, which will be posted to parents one week prior to the date on which the child would have turned 2 years old if they had been born at term (40 weeks). The Health and Social Care Information Centre (HSCIC) will be used to alert the Trial Co-ordinating Centre of deaths prior to follow-up.

To estimate the costs associated with the echocardiography/Doppler screening within 72 hours of birth in preterm babies, the following will be monitored;

- Staff resource used to carry out echocardiogram/Doppler screening within 72 hours compared with current practice
- The time, resource and unit costs associated with medication or tests and procedures as a result of earlier screening

- Requirement for neonatal medication
- Duration of stay in neonatal intensive care and inpatient days
- Admissions after discharge

Unit costs will be given to each resource item to determine an overall cost per baby. Primary cost data for many of the resources will be collected from participating hospital sites. Where possible other costs data such as cost of a clinician's time to perform an assessment will be collected from hospital finance departments. Most cost data is already available in published sources. For example, a study to investigate the costs of different levels of neonatal intensive care has already been carried out and other cost studies with relevant costs and costs associated with preterm delivery are available to supplement these [Petrou et al, 2003; Roberts et al, 2012].

10.3. Statistical Analysis

10.3.1. Primary Analysis Population

Babies will be analysed in the groups to which they are randomly assigned, comparing the outcome of all babies allocated to ibuprofen with all those allocated to placebo, regardless of deviation from the protocol or treatment received (referred to as the Intention to Treat (ITT) population).

10.3.2. Statistical Methods

Demographic and clinical data will be summarised with counts and percentages for categorical variables, means (standard deviations) for normally distributed continuous variables and medians (with interquartile or simple ranges) for other continuous variables. All comparative analyses will be performed adjusting for minimisation factors at randomisation [Brennen CK and Morris TP, 2013]. The adjusted analysis will also account for the correlation of outcomes among babies from multiple births included in the trial. Binary outcomes will be analysed using log binomial regression models. Results will be presented as adjusted risk ratios plus confidence intervals. If the model does not converge, then centre will be removed as a stratification factor in the first instance. If the model is still unstable then log Poisson regression models with robust variance estimation will be used [Zou G, 2014]. Continuous outcomes will be analysed using linear regression models and results will be presented as adjusted differences in means (plus confidence intervals). Analysis of time to event outcomes will use survival analysis techniques.

Process outcomes including the number of doses received, adherence to the protocol and study withdrawals will be summarised with counts and percentages for categorical variables, means (standard deviations) for normally distributed continuous variables and medians (with interquartile or simple ranges) for other continuous variables.

10.3.3. Pre-specified Subgroup Analysis

Pre-specified subgroup analysis will use the statistical test of interaction (or test for trend) and where appropriate, results will be presented as risk ratios with confidence intervals. Pre-specified subgroups will be based on gestational age, size of the PDA and mode of respiratory support.

10.3.4. Level of Statistical Significance

95% confidence intervals will be used for all primary outcome comparisons including subgroup analysis; to take account of the multiplicity of secondary outcomes, 99% confidence intervals will be presented.

10.3.5. Dealing with Missing Data

Missing data as a result of babies being lost to follow-up is expected to be minimal for short term outcomes. For 2 year outcomes, all reasonable measures will be taken to minimise loss to follow-up which is expected to be no more than 20% (excluding deaths after randomisation). Babies for whom no 2 year follow-up data are received will be compared to babies with 2 year data on demographic and clinical characteristics as well as short term outcomes, to assess generalisability. As there is expected to be a link between severity of disability and loss to follow-up, imputation techniques will not provide any meaningful information.

10.4. Economic Analysis

Health economic outcomes will take the form of a cost-effectiveness analysis of deaths and any moderate or severe BPD at 36 weeks postmenstrual age avoided, as well as analysis of the cost implications of secondary outcomes. Analysis will be from the perspective of the NHS so only direct NHS costs will be collected.

To determine economic outcomes, a within trial analysis will be conducted but a model based analysis beyond the end point of the trial will also be considered.

The within trial analysis will be based on two clinical endpoints. The first within trial analysis will be based on the composite clinical outcome of death avoided and/or case of moderate or severe BPD eliminated at 36 weeks postmenstrual age (this outcome can also be interpreted for the economic analysis as survival at 36 weeks postmenstrual age without severe or moderate BPD). The result of the economic evaluation will be reported as the additional cost per additional case of death or severe or moderate BPD avoided at 36 weeks postmenstrual age compared to conventional treatment. This analysis will only use data collected up to the assessment at 36 weeks postmenstrual date or discharge, whichever is later.

The second within trial analysis will be based on the clinical endpoint assessed at two years of age, of survival without severe or moderate neurodevelopmental disability. The analysis will include all cost and resource use data up to the infant reaching 2 years of age, based on data from the parent report and neurodevelopment assessment. It may be deemed appropriate to model beyond the end point of the trial if sufficient data are available. However the limitations of modelling beyond this point will be emphasised.

A bootstrapping approach to calculate the confidence intervals around differences in costs will be used to account for the skew inherent in most cost data. As a first step the analysis will take the form of a cost-consequences analysis, reporting data in a disaggregated manner on the cost and important consequences as determined in the trial. If a situation of dominance exists where, for example, the new intervention is more costly but less effective than the current intervention (dominated by the existing intervention) or conversely less costly but more effective than the existing intervention (the new intervention dominates the existing intervention), then the cost consequence analysis would establish that no further analysis is required. However, it is more likely that any additional benefit will be accompanied by additional costs and so a full incremental economic evaluation in terms of a cost effectiveness analysis will be carried out and the results presented in terms of additional cost per additional unit of effect.

10.5. Measures to Minimise Bias

The allocation of trial treatment is randomly assigned and concealed using a central secure web-based system and trials medications are masked such that medical and nursing staff, as well as outcome assessors and parents will be unaware of the trial medication administered. No crossover of groups is allowed; however rescue treatment will be permitted if certain pre-defined criteria are met.

11. Source Data/Documents

Direct access to source data/documents (including hospital records/notes, clinical charts, laboratory reports, pharmacy records and test reports) will be granted to authorised representatives from the NPEU CTU, the Sponsor, the MHRA and the host organisation to permit trial-related monitoring, audits and inspections.

12. Quality Control and Assurance

12.1. Risk Assessment

The NPEU CTU has performed a risk assessment of the trial prior to commencement that will be reviewed at regular intervals during the course of the trial.

12.2. National Registration Systems

The trial will be registered on at least one global trial register.

An International Standard Randomised Controlled Trial Number (ISRCTN) has also been sought.

All babies will be registered on the HSCIC register.

12.3. Site Initiation and Training

Initiation visits at each participating neonatal unit will be performed by the Chief Investigator or his delegate and a Local Research Nurse (LRN) once all appropriate approvals are in place and IMP has been shipped to the site to train site staff on trial procedures.

The LRN will ensure adherence to the protocol and deal with any specific site issues. They will also be responsible for organising trial days to ensure that all appropriate site staff are kept fully appraised of issues such as recruitment status, informed consent, data collection, follow-up and changing regulations.

12.4. Site Monitoring and Auditing

The LRN, along with the PI, will facilitate the day to day smooth running of the trial at the site. They will encourage recruitment, provide staff education and training, and monitor data completeness and quality.

The LRN will submit written site visit reports to an appropriate representative of the Project Management Group (PMG) based at the NPEU CTU. No routine monitoring will be carried out unless there is cause for concern regarding the conduct of the trial at a site as a result of central monitoring. Similarly, sites will only be audited if there is a reason. This level of monitoring is justified by the level of risk associated with the trial and the use of IMP.

12.5. Blinded Endpoint Review

Given the subjective nature and complexity of diagnosis for the outcomes listed below, a small number of clinicians, as well as an independent radiologist, will review all of the data relating to outcomes listed below for the internal pilot phase and at least 10% for the main trial. The outcomes are:

- Severe intraventricular haemorrhage (IVH) (grade 3/4 with ventricular dilation or intraparenchymal bleeding)
- Cystic periventricular leukomalacia (PVL)
- NEC definitive and/or complicated (Bell stage II and above) confirmed by radiography and / or histopathology.

13. Serious Breach of Good Clinical Practice or the Trial Protocol

The MHRA require that they be informed of all serious breaches in good clinical practice (GCP) or the trial protocol within 7 days of the Sponsor becoming aware of the breach.

A serious breach is defined as a breach of GCP or the trial protocol which is likely to affect to a significant degree –

- The safety or physical or mental integrity of the patient on the trial or
- The scientific value of the trial

In the event that a serious breach is suspected the Trial Co-ordinating Centre should be contacted as soon as possible. The Trial Co-ordinating Centre will refer the serious breach onto the Sponsor immediately.
The Chief Investigator or their delegate will also notify any protocol violations to the Sponsor and will notify the REC of these in accordance with trial procedures.

14. Ethics

14.1. Declaration of Helsinki

The Investigators will ensure that this trial is conducted in accordance with relevant regulations and with Good Clinical Practice.

14.2. Guidelines for Good Clinical Practice

The Investigator will ensure that this trial is conducted in accordance with relevant regulations and with Good Clinical Practice.

14.3. Approvals

The trial will only start after gaining approval from the MHRA and a registered REC. Additionally, approval of the appropriate NHS Trust Research and Development Office will be sought for individual trial sites.

Applications will be submitted through the Integrated Research Application System (IRAS).

A copy of the protocol, Parent Information Leaflet and Informed Consent Form, GP letter will be submitted to the MHRA and the REC for approval. The Chief Investigator or their delegate will submit and, where necessary, obtain approval from the MHRA and REC for any substantial amendments. Substantial amendments are defined as those that affect:

- the safety or physical or mental integrity of the participants of the trial;
- the scientific value of the trial;

- the conduct or management of the trial; or
- the quality or safety of any investigational medicinal product used in the trial.

14.4. Participant Confidentiality, Data Handling and Record Keeping

Overall responsibility for ensuring that each participant's information is kept confidential will lie with the trial sponsor. All paper documents will be stored securely and kept in strict confidence in compliance with the Data Protection Act (1998). Data collected on the data collection forms (DCFs) will be transferred for storage in an electronic database held by the Trial Co-ordinating Centre in which the participant will be identified only by a trial specific number.

Contact details of the baby's parent(s), as well as the baby's name (if known) and any other identifying details will be stored in a separate database also held at the NPEU CTU. This database will only be linked to the database containing trial data by the baby's trial number.

After the trial has been completed and the reports published, the data will be archived in a secure physical or electronic location with controlled access.

Electronic files will be stored on a file server that has restricted access. The server is in a secure location and access is restricted to a few named individuals. Access to the building in which the NPEU CTU is situated is via an electronic tag and individual rooms are kept locked when unoccupied. Authorisation to access restricted areas of the NPEU CTU network is as described in the NPEU CTU security policy. Data will be processed on a workstation by authorised staff. The computer workstations access the network via a login name and password which is changed regularly. No data are stored on individual workstations. Back-up of data is done automatically overnight to an offsite storage area. The location of the back-up computer is in a separate department which has electronic tag access. Access to the room in which the back-up machine is located is via a key-pad system.

14.5. Retention of Personal Data

Personal data will be needed to contact parents when their children are 2 years of age, to co-ordinate follow-up, and to disseminate the results of the trial to parent(s). Due to the nature of neonatal research the NPEU policy is to keep personal data for a period of no less than 25 years in order to follow-up on health related issues which may become relevant in the future. At all times personal data will be held securely and will not be used for any other purpose.

14.6. Funding

The National Institute for Health Research (NIHR) Health Technology Assessment (HTA) programme is funding the trial.

14.7. Insurance

The University has a specialist insurance policy in place which would operate in the event of any participant suffering harm as a result of their involvement in the research (Newline Underwriting Management Ltd, at Lloyd's of London). NHS indemnity operates in respect of the clinical treatment which is provided.

15. Trial Governance

15.1. Site Research and Development Approval

Individual sites will only commence recruiting participants once they receive approval from NHS Trust Research and Development (R&D) Offices. Applications to R&D offices will be submitted through the NIHR Co-ordinated System for gaining NHS permission.

15.2. Trial Sponsor

The University of Oxford is the nominated Sponsor for the trial.

15.3. Co-ordinating Centre

The trial co-ordinating centre will be at the NPEU CTU, University of Oxford where the Trial Co-ordinator will be based. The NPEU CTU will be responsible for all trial programming, randomisation, data entry, statistical analyses and, in collaboration with the Chief Investigator and the Local Research Nurse(s), manage the day-to-day running of the trial including recruitment of centres and training of staff. The NPEU CTU will also service both the DMC and TSC.

15.4. Project Management Group (PMG)

The trial will be supervised on a day-to-day basis by the Project Management Group. This group reports to the Trial Steering Committee (TSC) which is responsible to the trial sponsor. At each participating centre, a local Principal Investigator will report to the PMG via the staff based at the NPEU CTU.

The core PMG will consist of the CI and NPEU CTU staff including:

- CTU Director
- Senior Trials Manager
- Senior Trials Programmer
- Trial Co-ordinator
- Trial Statistician
- Trial Programmer
- Administrator/Data Manager

The core PMG will meet regularly (at least monthly).

15.5. Co-investigators' Group (CIG)

The CIG will meet at least twice a year. This will comprise all co-applicants and the members of the core PMG.

15.6. Trial Steering Committee (TSC)

The trial will be overseen by a TSC consisting of an independent chair and at least two other independent members. Committee members will be deemed to be independent if they are not involved in trial recruitment and are not employed by any organisation directly involved in the trial conduct.

Representatives from relevant Patient/Public Involvement groups, the Chief Investigator, other Investigators/co-applicants will be joined by observers from the NPEU CTU. The HTA programme manager will be invited to attend all TSC meetings.

The role of the TSC is to provide the overall supervision of the trial. The TSC should monitor the progress of the trial and conduct and advise on its scientific credibility. The TSC will consider and act, as appropriate, upon the recommendations of the DMC and ultimately carries the responsibility for deciding whether a trial needs to be stopped on grounds of safety or efficacy.

15.7. Data Monitoring Committee (DMC)

A DMC, independent of the applicants and of the TSC, will review the progress of the trial at least annually and provide advice on the conduct of the trial to the TSC and (via the TSC) to the HTA. The committee will periodically review trial progress and outcomes as well as secondary outcomes (e.g. death, severe IVH, etc.). The content and timings of the DMC reviews will be detailed in a DMC Charter, which will be agreed at its first meeting.

16. Publication Policy/Acknowledgement of Contribution

The success of the trial depends on a large number of neonatal nurses, neonatologists, and parent(s). Credit for the trial findings will be given to all who have collaborated and participated in the trial including all local co-ordinators and collaborators, members of the trial committees, the Baby-OSCAR Co-ordinating Centre and trial staff. Authorship at the head of the primary results paper will take the form “[name], [name] and [name] on behalf of the ‘The Baby-OSCAR Collaborative Group’”. The drafting of the paper will be the responsibility of a writing committee. All contributors to the trial will be listed at the end of the main paper, with their contribution identified.

17. Protocol Signature

17.1. Principal Investigator Signature

By signing this protocol signature page, I agree to:

- Conduct the trial in accordance with the protocol and only make changes in order to protect the safety, rights or welfare of the participants.
- Personally conduct or supervise the trial and ensure that all associates, colleagues and employees assisting in the conduct of the trial are informed about their obligations.
- Ensure requirements with regard to obtaining informed consent are adhered to.
- Report AEs/SAEs that occur during the course of the trial and maintain adequate and accurate records to enable representatives of the Sponsor or regulatory authority to confirm adherence with the protocol.

______________________________________	________________
Principal Investigator's Signature	Date

18. References

Aranda JV, Clyman R, Cox B, Van Overmeire B, Wozniak P, Sosenko I, et al. A randomized, double-blind, placebo-controlled trial on intravenous ibuprofen L-lysine for the early closure of nonsymptomatic patent ductus arteriosus within 72 hours of birth in extremely low-birth-weight infants. *Am J Perinatol* 2009;26(3):235-45.

Bancalari E, Claure N, Gonzalez A. Patent ductus arteriosus and respiratory outcome in premature infants. *Biol Neonate* 2005;88(3):192–201.

Bose CL, Laughon MM. Patent ductus arteriosus: lack of evidence for common treatments. *Arch Dis Child Fetal Neonatal Ed* 2007;92(6):F498–502.

Brennen CK, Morris TP. Analysis of multicentre trials with continuous outcomes: when and how should we account for centre effects? Statistics in Medicine 2013; 32: 1136-1149.

Clyman RI. The role of patent ductus arteriosus and its treatment in the development of bronchopulmonary dysplasia. Seminars in Perinatology 2013; 37:102–7.

Clyman RI, Cassady G, Kirklin JK, Collins M, Philips JB, 3rd. The role of patent ductus arteriosus ligation in bronchopulmonary dysplasia: reexamining a randomized controlled trial. *J Pediatr* 2009;154(6):873–6.

Clyman RI, Chorne N. Patent ductus arteriosus: evidence for and against treatment. *J Pediatr* 2007;150(3):216–9.

Clyman RI, Saha S, Jobe A, Oh W. Indomethacin prophylaxis for preterm infants: the impact of 2 multicentered randomized controlled trials on clinical practice. *J Pediatr* 2007;150(1):46–50 e2.

Cooke L, Steer PA, Woodgate PG. Indomethacin for asymptomatic patent ductus arteriosus in preterm infants. *Cochrane Database of Systematic Reviews* 2003, Issue 1. Art. No.: CD003745. DOI: 10.1002/14651858.CD003745

Dereddy N. Surgical ligation for patent ductus arteriosus. *J Pediatr* 2011;158(2):343

Fowlie PW, Davis PG, McGuire W. Prophylactic intravenous indomethacin for preventing mortality and morbidity in preterm infants. *Cochrane Database of Systematic Reviews* 2010, Issue 7. Art. No.: CD000174. DOI: 10.1002/14651858.CD000174.pub2.

Hamrick SE, Hansmann G. Patent ductus arteriosus of the preterm infant. *Pediatrics* 2010;125(5):1020–30.

Jones LJ, Craven PD, Attia J, Thakkinstian A, Wright I. Network meta-analysis of indomethacin versus ibuprofen versus placebo for PDA in preterm infants. *Arch Dis Child Fetal Neonatal Ed* 2011;96(1):F45–52.

Kluckow M, Evans N. Early echocardiographic prediction of symptomatic patent ductus arteriosus in preterm infants undergoing mechanical ventilation. *J Pediatr* 1995;127(5):774–9.

Martin AJ, Darlow BA, Salt A, Hague W, Sebastian L, McNeill N, Tarnow-Mordi W.

Performance of the Parent Report of Children's Abilities-Revised (PARCA-R) versus the Bayley Scales of Infant Development III. *Arch Dis Child Fetal Neonatal Ed*. 2013; 98: 955–8.

Mangham LJ, Petrou S, Doyle LW, Draper ES, Marlow N, The cost of preterm birth throughout childhood in England and Wales. *Pediatrics*. 2009;123(2):e312–27.

Nemerofsky SL, Parravicini E, Bateman D, Kleinman C, Polin RA, Lorenz JM. The ductus arteriosus rarely requires treatment in infants > 1000 grams. *Am J Perinatol. 2008 Nov;25(10):661-6. doi: 10.1055/s-0028-1090594. Epub 2008 Oct 10.*

Neonatal Survey Database from the Trent Region 2010; http://www.le.ac.uk/departments/health-sciences/research/timms/projects/tns

Noori S, McCoy M, Friedlich P, Bright B, Gottipati V, Seri I, Sekar K. Failure of ductus arteriosus closure is associated with increased mortality in preterm infants. *Pediatrics* 2009;123;138-e144.

Noori S. Patent ductus arteriosus in the preterm infant: to treat or not to treat? *J Perinatol* 2010;30 Suppl:S31−7.

Ohlsson A, Shah SS. Ibuprofen for the prevention of patent ductus arteriosus in preterm and/or low birth weight infants. *Cochrane Database of Systematic Reviews* 2011(1):CD004213.

Ohlsson A, Walia R, Shah SS. Ibuprofen for the treatment of patent ductus arteriosus in preterm and/or low birth weight infants. *Cochrane Database of Systematic Reviews* 2010(4):CD003481.

Oncel MY, Yurttutan S, Degirmencioglu H, Uras N, Altug N, Erdeve O, Dilmen U: Intravenous paracetamol treatment in the management of patent ductus arteriosus in extremely low birth weight infants. *Neonatology* 2013;103:166–9.

Oncel MY, Yurttutan S, Uras N, Altug N, Ozdemir R, Ekmen S, Erdeve O, Dilmen U: An alternative drug (paracetamol) in the management of patent ductus arteriosus in ibuprofen-resistant or contraindicated preterm infants. *Arch Dis Child Fetal Neonatal Ed* 2013;98:94.

Petrou S, Eddama O, Mangham L. A structured review of the recent literature on the economic consequences of preterm birth. *Arch Dis Child Fetal Neonatal Ed* 2011; 96; F225−32.

Petrou S, Mehta Z, Hockley C, Cook-Mozaffari P, Henderson J, Goldacre M. The impact of preterm birth on hospital inpatient admissions and costs during the first 5 years of life. Pediatrics. 2003; 112 (6 Pt1);1290–7.

Roberts TE, Barton PM, Auguste PE, Middleton LJ, Furmston AT, Ewer AK. Pulse oximetry as a screening test for congenital heart defects in newborn infants: a cost-effectiveness analysis. *Arch Dis Child* 2012;97(3) :221–6.

Schmidt B, Davis P, Moddemann D, Ohlsson A, Roberts RS, Saigal S, et al. Long-term effects of indomethacin prophylaxis in extremely-low-birth-weight infants. *N Engl J Med* 2001;344(26):1966–72.

Schmidt B, Roberts RS, Fanaroff A, Davis P, Kirpalani HM, Nwaesei C, et al. Indomethacin prophylaxis, patent ductus arteriosus, and the risk of bronchopulmonary dysplasia: further analyses from the Trial of Indomethacin Prophylaxis in Preterms (TIPP). *J Pediatr* 2006;148(6):730–4.

Sellmer A, Vandborg Bjerre J, Schmidt MR, McNamara PJ, Hjortdal VE, Host B, Bech BH, Henriksen TB: Morbidity and mortality in preterm neonates with patent ductus arteriosus on day 3. *Arch Dis Child Fetal Neonatal* Published online first 26 July 2013; 10.1136/archdischild-2013-303816.

Stoll BJ, Hansen NI, Bell EF, Shankaran S, Laptook AR, Walsh MC, et al. Neonatal outcomes of extremely preterm infants from the NICHD Neonatal Research Network. *Pediatrics* 2010;126(3):443–56.

Su BH, Lin HC, Chiu HY, Hsieh HY, Chen HH, Tsai YC. Comparison of ibuprofen and indometacin for early-targeted treatment of patent ductus arteriosus in extremely premature infants: a randomised controlled trial. *Arch Dis Child Fetal Neonatal Ed* 2008;93(2):F94–9.

Su BH, Watanabe T, Shimizu M, Yanagisawa M. Echocardiographic assessment of patent ductus arteriosus shunt flow pattern in premature infants. *Arch Dis Child Fetal Neonatal Ed* 1997;77(1):F36–40.

Sweet DG, Carnielli V, Greisen G, Hallman M, Ozek E, Plavka R, et al. European consensus guidelines on the management of neonatal respiratory distress syndrome in preterm infants - 2013 update. *Neonatology.* 2013;103(4):353–68.

Tin W, Wariyar U, Hey E. Changing prognosis for babies of less than 28 weeks' gestation in the north of England between 1983 and 1994. Northern Neonatal Network. *BMJ* 1997;314(7074):107–11.

Van Overmeire B, Allegaert K, Casaer A, Debauche C, Decaluwe W, Jespers A, et al. Prophylactic ibuprofen in premature infants: a multicentre, randomised, double-blind, placebo-controlled trial. *Lancet* 2004;364(9449):1945–9.

Zou G. A modification poisson regression approach to prospective studies with binary data. *Am J Epidemiol. 2004 Apr 1;159(7):702-6.*

Contact details

NPEU Clinical Trials Unit
National Perinatal Epidemiology Unit
Nuffield Department of Population Health
University of Oxford
Old Road Campus
Headington
OXFORD, OX3 7LF

T: 01865 617 965
F: 01865 289 740
E: baby-oscar@npeu.ox.ac.uk
W: www.npeu.ox.ac.uk/ctu

Follow us on Twitter @NPEU_CTU

Watch us on YouTube: http://youtube.com/NPEUOxford

Baby-OSCAR is funding by the National Institute for Health Research HTA Programme (project reference 11/92/15)

Outcome after Selective Early Treatment for Closure of Patent Ductus ARteriosus in Preterm Babies

PROTOCOL

Chief Investigator:

Professor Samir Gupta
Clinical Professor of Neonatology
Durham University
Consultant Neonatologist
University Hospital of North Tees
Hardwick Road
Stockton-On-Tees TS19 8PE
Tel: 01642 624 232

Clinical Trials Unit:

National Perinatal Epidemiology Unit
Nuffield Department of Population Health
University of Oxford
Old Road Campus Oxford
OX3 7LF
Tel: 01865 617 965

Funder:

National Institute of Health Research – Health Technology Assessment Programme (project reference 11/92/15)

Sponsor:

University of Oxford

Trial Identifiers:

EudraCT No.:	2013-005336-23
ISRCTN:	84264977
IRAS No.:	142310

Table of Contents

1. Protocol Synopsis 5
2. Trial Flow Diagram 10
3. Abbreviations 11
4. Introduction 13
4.1. Background and Rationale 13
4.2. Current Evidence Supporting Trial Rationale 14
5. Trial Objective 15
5.1. Primary Objective 15
5.2. Secondary Objectives 16
6. Trial Design 16
6.1. Summary 16
6.2. Inclusion Criteria 17
6.3. Exclusion Criteria 17
6.4. Setting 17
6.5. Primary Outcome 18
6.5.1. Oxygen Reduction Test 18
6.6. Secondary Outcomes 19
6.7. Process Outcomes 20
7. Trial Procedures 20
7.1. Trial Assessments 21
7.2. Structure and Duration of the Trial 22
7.3. Initial Eligibility Assessment 22
7.4. Echocardiograms 23
7.5. Informed Consent 24
7.6. Randomisation 24
7.7. Concomitant Medications 24
7.8. Permitted and Non-Permitted Medications 25
7.8.1. Supportive Care of Enrolled Babies 25
7.9. Stopping Trial Medication 25
7.10. Out of Hours 26
7.11. Breakages and Spoilt Ampoules 26
7.12. End of Trial 26
7.13. Early Trial Cessation 26
7.14. Remuneration 27
8. Investigational Medicinal Product (IMP) 27
8.1. Dosing and Administration 27
8.2. Distribution 27
8.3. Accountability 27
8.4. Open-label Treatment 28
8.5. Masking of Trial Medication 28

8.6. Emergency Procedure for Unmasking / Envelopes ... 28
9. Safety Reporting ... 29
9.1. Definitions ... 29
9.1.1. Adverse Event (AE) ... 29
9.1.2. Adverse Reaction (AR) ... 29
9.1.3. Serious Adverse Event (SAE) ... 29
9.1.4. Foreseeable Serious Adverse Events ... 30
9.1.5. Unforeseeable Serious Adverse Events ... 31
9.1.6. Serious Adverse Reaction (SAR) ... 31
9.1.7. Suspected Unexpected Serious Adverse Reaction (SUSAR) ... 31
9.1.8. Causality ... 31
9.1.9. Assessment of Safety ... 32
9.2. Reporting Procedures ... 32
9.2.1. AE/SAE Reporting ... 32
9.2.2. SUSAR Reporting ... 32
9.2.3. Development Safety Update Report (DSUR) ... 33
10. Statistics and Analysis ... 33
10.1. Sample Size ... 33
10.2. Assessment of Outcomes ... 35
10.3. Statistical Analysis ... 35
10.3.1. Primary Analysis Population ... 35
10.3.2. Statistical Methods ... 36
10.3.3. Pre-specified Subgroup Analysis ... 37
10.3.4. Level of Statistical Significance ... 37
10.3.5. Dealing with Missing Data ... 37
10.4. Economic Analysis ... 38
10.5. Measures to Minimise Bias ... 39
11. Source Data/Documents ... 39
12. Quality Control and Assurance ... 39
12.1. Risk Assessment ... 39
12.2. National Registration Systems ... 39
12.3. Site Initiation and Training ... 39
12.4. Site Monitoring and Auditing ... 40
13. Serious Breach of Good Clinical Practice or the Trial Protocol ... 40
14. Ethics ... 40
14.1. Declaration of Helsinki ... 40
14.2. Guidelines for Good Clinical Practice ... 40
14.3. Approvals ... 40
14.4. Participant Confidentiality, Data Handling and Record Keeping ... 41
14.5. Retention of Personal Data ... 42
14.6. Funding ... 42

14.7. Insurance 42
15. Trial Governance 42
15.1. Site Research and Development Approval 42
15.2. Trial Sponsor 42
15.3. Co-ordinating Centre 42
15.4. Project Management Group (PMG) 42
15.5. Co-investigators' Group (CIG) 43
15.6. Trial Steering Committee (TSC) 43
15.7. Data Monitoring Committee (DMC) 43
16. Publication Policy/Acknowledgement of Contribution 44
17. References 45
18. Appendix: Amendment History 48

1. Protocol Synopsis

**Trial Title:**	Outcome after Selective Early Treatment for Closure of Patent Ductus ARteriosus in Preterm Babies [Baby-OSCAR Trial]
**Internal Reference:**	Baby-OSCAR
**Clinical Phase:**	Phase III
**Trial Design:**	Multi-centre, masked, randomised placebo-controlled parallel group trial to determine short and long term health and economic outcomes of the treatment of a large Patent Ductus Arteriosus (PDA) in extremely preterm babies with ibuprofen within 72 hours of birth.<br>The main trial will be conducted after an internal pilot phase, which will be run to assess the suitability of trial procedures and likelihood of recruitment targets being achieved.
**Trial Participants:**	Extreme preterm babies with a large PDA confirmed using echocardiography.
**Inclusion Criteria:**	Babies will be considered eligible for inclusion in the trial if they are:<br>• Born at 23^{+0} to 28^{+6} weeks of gestation<br>• Less than 72 hours old<br>• Confirmed by echocardiography to have a large PDA which<br>- is at least 1.5 mm in diameter (determined by gain optimised colour Doppler),<br>*and*<br>- has unrestrictive pulsatile (left to right) flow (ratio of flow velocity in PDA Maximum (V_{max}) to Minimum (V_{min}) > 2:1)) or, growing flow pattern (< 30% right to left), and **no clinical concerns of pulmonary hypertension**<br>In addition:<br>• The responsible clinician is uncertain about whether the baby might benefit from treatment to close the PDA<br>• Written informed consent has been obtained from the parent(s).
**Exclusion Criteria:**	Babies will be excluded from participation in the trial if they have:<br>• No realistic prospect of survival<br>• Severe congenital anomaly<br>• Clinical or echocardiography suspicion of congenital structural

	heart disease that contraindicates treatment with ibuprofen<br>• Other conditions that would contraindicate the use of ibuprofen (active bleeding especially intracranial or gastrointestinal bleeding, coagulopathy, thrombocytopenia (platelet count <50,000), renal failure, life threatening infection, pulmonary hypertension, known or suspected necrotising enterocolitis (NEC))<br>• Indomethacin, ibuprofen, or paracetamol administration after birth
**Sample Size:**	Approximately 730 preterm babies in total (including those recruited during the internal pilot phase).<br>365 babies per treatment arm.
**Trial Sites:**	Approximately 30 neonatal units. 5 sites for the internal pilot phase.
**Trial Period:**	Trial period for an individual baby is defined as randomisation to 2 years of age corrected for prematurity.<br>For the purposes of regulatory notification, end of trial is defined as the last follow-up assessment at 2 years of age corrected for prematurity. The entire trial is anticipated to take 97 months to complete (including set-up, internal pilot phase and reporting).<br>Adverse Events which are serious will be recorded from first dose until 7 days after trial medication. Only Unforeseeable SAEs will be reported.
**Primary Objective:**	To determine if the selective treatment of echocardiographically confirmed large PDAs in extremely preterm babies with ibuprofen within 72 hours of birth reduces the incidence of death by 36 weeks of postmenstrual age, or moderate or severe bronchopulmonary dysplasia (BPD) at 36 weeks of postmenstrual age.
**Primary Endpoints:**	Composite outcome of incidence of death by 36 weeks of postmenstrual age, or moderate or severe BPD at 36 weeks of postmenstrual age.
**Secondary Objectives:**	To determine if the selective treatment of confirmed large PDAs in extremely preterm babies with ibuprofen within 72 hours of birth results in:<br>• A reduction in the components of the primary outcome: death by 36 weeks of postmenstrual age; moderate or severe BPD at 36 weeks of postmenstrual age, severity of BPD at 36 weeks of postmenstrual age; other secondary outcomes up to discharge (see Secondary Endpoints);<br>• Improved health outcomes at 2 years corrected age including survival without moderate or severe neurodevelopmental

	disability (long-term primary objective) and survival without respiratory morbidity (long-term secondary objective).<br>An economic evaluation will be carried out from the perspective of the health service. It will take the form of a cost-effectiveness analysis presented in terms of cost per major outcome averted. The major outcomes are those of the primary outcome, namely death and any moderate or severe BPD by 36 weeks of postmenstrual age. Additional analyses will take place on a range of secondary outcomes and on neurodevelopmental outcomes at 2 years. The incremental cost estimate for statistically significant differences in the pre-specified outcomes in primary and subgroup analyses would be computed.
**Secondary Endpoints:**	**Short Term Outcomes**<br>• Death by 36 weeks of postmenstrual age<br>• Moderate or severe BPD at 36 weeks of postmenstrual age<br>• Severity of BPD at 36 weeks of postmenstrual age (see table in Section 6.5)<br>Incidence or duration of the following up to discharge:<br>• Severe intraventricular haemorrhage (IVH) (grade III/IV with ventricular dilatation or intraparenchymal abnormality)<br>• Cystic periventricular leukomalacia (PVL)<br>• Non-cystic PVL<br>• Hydrocephalus<br>• Babies treated for Retinopathy of prematurity (ROP)<br>• Significant pulmonary haemorrhage (fresh blood in endotracheal tube with increase in respiratory support)<br>• Treated for Pulmonary hypertension with pulmonary vasodilator<br>• NEC definitive and/or complicated (Bell stage II and above) confirmed by radiography and/or histopathology<br>• NEC requiring surgery<br>• Gastrointestinal bleeding (leading to investigation or clinical treatment) within 7 days of the first dose of trial drug administration<br>• Spontaneous intestinal perforation<br>• Closed or non-significant PDA (<1.5 mm) at around 3 weeks of age (range of 18 – 24 days), confirmed by ECHO<br>• PDA ≥ 1.5 mm at around 3 weeks' (range of 18 – 24 days)

	• Medical open-label treatment of a symptomatic PDA with a COX inhibitor<br>• Open-label treatment of a symptomatic PDA by surgical treatment<br>• Administration and duration of inotropic support<br>• Total duration of respiratory support<br>a) Invasive ventilation through an endotracheal tube<br>b) Non-invasive support through, nasal CPAP, nasal ventilation, humidified high flow nasal cannula therapy, or low flow oxygen ≥ 1.1 L/min<br>• Discharge home on oxygen<br>• Duration of initial hospitalisation (birth to discharge home)<br>• Postnatal steroid use for chronic lung disease<br>• Tolerance of ibuprofen treatment within the foreseeable SAE reporting range, described in the protocol, section 9.1.4<br>• Weight gain: a change in z score between birth and discharge (or death if sooner)<br>• Head circumference: a change in head size z score between randomisation and discharge (or death if sooner)<br>**Long Term Outcomes assessed at 2 years of age corrected for prematurity**<br>• Survival without moderate or severe neurodevelopmental disability (main long term outcome)<br>• Survival<br>• Individual components of survival without moderate or severe neurodevelopmental disability (in the four domains of motor, cognitive, hearing and visual function). Cognitive disability will be assessed by determining the standardised non-verbal cognitive subscale and language subscale scores obtained through the Parent Report of Cognitive Abilities-Revised (PARCA-R) assessment. The PARCA-R assessment will be adapted to include questions to assess gross motor, hearing and visual function.<br>• Survival without respiratory morbidity. Respiratory morbidity will be assessed by the need for oxygen or respiratory support; presence of persistent cough and/or wheeze; need for regular treatment for respiratory illness; unscheduled attendances at hospital/GP; readmission to hospital for respiratory problems.

	• Duration of oxygen supplementation from randomisation<br>A cost-effectiveness analysis will be conducted of deaths and BPD events avoided and national health services used up to 2 years of age corrected for prematurity.
**Process Outcomes**	Process outcomes will be the following;<br>• Number of doses of trial medication received<br>• Adherence to protocol (e.g. protocol violations, incidence of non-symptomatic open-label treatment etc.)<br>• Study withdrawals
**Investigational Medicinal Product:**	Ibuprofen will be provided as a clear sterile solution for intravenous injection. An initial dose of 10 mg/kg will be followed by two doses of 5 mg/kg at 24 and 48 hours after the initial dose. The solution of ibuprofen is provided at a concentration of 5 mg/ml in a single-use 2 ml ampoule, thus 2 ml/kg, followed by two administrations of 1 ml/kg will be required.<br>Placebo will be supplied as a clear sterile solution of 0.9% Sodium Chloride for injection. The solution will be indistinguishable from that of ibuprofen. It will be given as a 2 ml/kg infusion followed by two infusions of 1 ml/kg at 24 and 48 hours.<br>Doses to be calculated on birth weight and administered as a short infusion over 15 minutes, preferably undiluted. If required the IMP can be diluted to appropriate volume with 5% glucose or 0.9% Sodium Chloride and first dose administered soon after randomisation, after 6 hours of age and within 72 hours of birth.<br>Open-label treatment will be permitted if defined clinical and echocardiography criteria are met.

2. Trial Flow Diagram

Inclusion criteria

- Born at 23^{+0} to 28^{+6} weeks of gestation
- Less than 72 hours old
- Confirmed by echocardiography to have a large PDA which
  - is at least 1.5 mm in diameter (determined by gain optimised colour Doppler), *and*
  - has unrestrictive pulsatile (left to right) flow (ratio of flow velocity in PDA Maximum (V_{max}) to Minimum (V_{min}) > 2:1)) or, growing flow pattern (< 30% right to left), and no clinical concerns of pulmonary hypertension
- The responsible clinician is uncertain about whether the baby might benefit from treatment to close the PDA
- Written informed consent has been obtained from the parent(s)

Exclusion criteria

- No realistic prospect of survival
- Severe congenital anomaly
- Clinical or echocardiography suspicion of congenital structural heart disease that contraindicates treatment with ibuprofen
- Contraindication to use of ibuprofen
- Indomethacin, ibuprofen, or paracetamol administration after birth

Randomisation (1:1 ratio)

Web-based randomisation hosted by NPEU Clinical Trials Unit

Active treatment

Ibuprofen

10, 5, 5 mg/kg each 24 hours apart

(2, 1, 1 ml/kg of 5 mg/ml solution)

OR

Placebo

0.9% Sodium Chloride

2, 1, 1 ml/kg each 24 hours apart

Primary Outcome by 36 weeks of postmenstrual age (PMA)

- Death, or
- Moderate or severe bronchopulmonary dysplasia (BPD)

Secondary Short Term Outcomes

- Death by 36 weeks of PMA
- Moderate or severe BPD at 36 weeks of PMA
- Severity of BPD at 36 weeks of PMA
- Other secondary outcomes (see protocol) up to discharge

Cost Effectiveness Analysis

At discharge from neonatal unit and at 2 years of age corrected for prematurity:

- Deaths and BPD events avoided
- NHS services used up to 2 years of age corrected for prematurity

Secondary Long Term Outcomes (at 2 years of age corrected for prematurity)

- Survival without moderate or severe neurodevelopmental disability (main long term outcome)
- Survival
- Individual components of survival without moderate or severe disability (in the four domains of motor, cognitive, hearing and visual function)
- Survival without respiratory morbidity
- Duration of oxygen supplementation from randomisation

3. Abbreviations

**ADR**	Adverse Drug Reaction
**AE**	Adverse Event
**AR**	Adverse Reaction
**ARR**	Absolute Risk Reduction
**BPD**	Bronchopulmonary Dysplasia
**CI**	Chief Investigator
**CIG**	Co-Investigator Group
**COX**	Cyclo-oxygenase
**CPAP**	Continuous Positive Airway Pressure
**DA**	Ductus Arteriosus
**DMC**	Data Monitoring Committee
**DSUR**	Development Safety Update Report
**ECHO**	Echocardiography
**GCP**	Good Clinical Practice
**GP**	General Practitioner
**HSCIC**	Health and Social Care Information Centre
**HRA**	Health Research Authority
**HTA**	Health Technology Assessment
**ICF**	Informed Consent Form
**ICH**	International Conference on Harmonisation
**IMP**	Investigational Medicinal Product
**IRAS**	Integrated Research Application System
**ITT**	Intention to Treat
**IVH**	Intraventricular Haemorrhage
**LCRN**	Local Clinical Research Network
**LRN**	Local Research Nurse
**MCRN**	Medicines for Children Research Network
**MHRA**	Medicines and Healthcare products Regulatory Agency
**nCPAP**	Nasal Continuous Positive Airway Pressure
**NEC**	Necrotising Enterocolitis
**NHS**	National Health Service
**NIHR**	National Institute for Health Research
**NPEU CTU**	National Perinatal Epidemiology Unit Clinical Trials Unit

**NSAID**	Non-Steroidal Anti-inflammatory Drug
**OR**	Odds Ratio
**PARCA-R**	Parent Report of Cognitive Abilities-Revised
**PDA**	Patent Ductus Arteriosus
**PI**	Principal Investigator
**PIL**	Parent Information Leaflet
**PMA**	Postmenstrual Age
**PMG**	Project Management Group
**PVL**	Cystic Periventricular Leukomalacia
**R&D**	NHS Trust Research and Development Department
**REC**	Research Ethics Committee
**RDS**	Respiratory Distress Syndrome
**ROP**	Retinopathy of Prematurity
**SAE**	Serious Adverse Event
**SAR**	Serious Adverse Reaction
**SmPC**	Summary of Product Characteristics
**SUSAR**	Suspected Unexpected Serious Adverse Reaction
**TSC**	Trial Steering Committee

4. Introduction

4.1. Background and Rationale

The Ductus Arteriosus (DA) is a vessel that allows blood from the right ventricle to bypass the fetal lungs to the placenta. In term babies it closes spontaneously after birth when breathing is established and is structurally closed after a few days. However, in a large number of preterm babies, the vessel does not close spontaneously resulting in a condition known as Patent Ductus Arteriosus (PDA). Around 7,000 extremely preterm babies (<29 weeks of gestation) are born in the UK every year. In 40% the PDA will fail to close spontaneously even by 4 months of age. [Nemerofsky et al, 2008].

PDA is associated with a number of serious and life-threatening short and long term complications including low blood pressure (hypotension), bleeding in the lungs (pulmonary haemorrhage) and brain (intraventricular haemorrhage (IVH)), systemic complications such as necrotising enterocolitis (NEC), bronchopulmonary dysplasia (BPD), and long term health problems such as neurodevelopmental disability and chronic respiratory problems. The persistence of PDA is associated with an 8-fold rise in neonatal mortality [Noori S et al, 2009]. In addition, as PDA is very common in extreme preterm babies and is associated with a prolonged need for respiratory support and hospitalisation, it places a significant financial burden on the National Health Service (NHS).

Historically, clinicians who have been concerned about the complications associated with a PDA have attempted to close PDAs utilising medical (pharmacological) or surgical treatment. Traditionally, medical treatment is instituted as prophylactic treatment (within 24 hours of birth) or symptomatic treatment (usually 5−7 days after birth). Prophylactic pharmacological treatment of all preterm babies unnecessarily exposes a large proportion of babies to the potentially serious side effects of drug treatment, when their PDA would have closed spontaneously. Symptomatic treatment on the contrary delays treatment while waiting for symptoms to appear and could result in a loss of treatment benefit as irreversible damage may have already been done.

Moreover, the practice of a conservative approach of not treating, seems to originate from uncertainty regarding the management of PDA rather than evidence favouring no intervention. This is due to the fact that most studies conducted to date have involved more mature preterm babies (over 1,000 g or 28 weeks of gestation) whose PDA is more likely to close spontaneously. The studies were also largely designed to assess PDA closure rates rather than clinically important outcomes.

It is now suggested that large PDAs (those with a diameter of ≥1.5 mm) through which blood flow is pulsatile and unrestricted are less likely to close spontaneously. Targeted early

treatment of large PDAs whilst asymptomatic has the potential to overcome the disadvantages of both the prophylactic and symptomatic approaches. Although clinical detection of PDA whilst asymptomatic is challenging, it can be assessed using bedside echocardiography.

Non-steroidal anti-inflammatory drugs, especially indomethacin and ibuprofen have been widely used for the treatment of PDA. Short term efficacy of indomethacin and ibuprofen are equivalent in the treatment of PDA [Su BH et al, 2008]. Ibuprofen however appears to reduce the risk of NEC and is associated with fewer clinical gastrointestinal and renal side effects compared to indomethacin, hence it is the drug of choice for this trial. Paracetamol, has also been recently reported in case studies for closure of symptomatic PDA but further research needs to be done to establish its effectiveness [Oncel MY et al, 2013].

The aim of this trial is to examine whether the pharmacological closure of a large PDA (identified by echocardiography) in extremely preterm babies whilst asymptomatic has a clinically important impact on both short and long term health and economic outcomes.

4.2. Current Evidence Supporting Trial Rationale

Although the number of extremely preterm babies that survive has increased due to improvements in neonatal care, the proportion of babies with moderate or severe disability has remained largely unchanged. Concern regarding this is reflected in the results of a survey conducted by the Medicines for Children Research Network (MCRN) which identified PDA and BPD as key areas in which clinicians believed further evidence and research is most needed.

To date, the majority of studies of prophylactic or symptomatic treatment of PDA have included babies up to 34 weeks of gestation, have been small in size, were designed to assess PDA closure rates rather than short or long term clinical outcomes and are now relatively old, all of which limit the ability to draw meaningful conclusions from the results. Furthermore, there are no recent trials reporting outcomes after selective early treatment of PDA based both on duct size and haemodynamic assessment. Thus the current literature falls short of providing substantive evidence on the management of PDA among extreme preterm babies leading to uncertainty and heterogeneity in clinical practices.

A recent cohort trial identified presence of a large PDA (defined as a PDA dimension of ≥1.5 mm) on day 3 in babies born before 28 weeks of gestation with threefold increase in odds of death or severe morbidity compared with neonates without PDA (Odds Ratio (OR) 3.4; 95% Confidence Interval (CI) 1.1 to 11.0). Neonates with a large PDA were also reported to have increased odds of IVH (OR 4.2; 95% CI 1.3 to 14.0) and BPD (OR 3.7; 95% CI 1.0 to 14.0) compared with neonates with no PDA [Sellmer A et al, 2013]. In preclinical trials, pharmacologic PDA closure is reported to improve alveolarisation and minimise the impaired postnatal alveolar development that is the pathologic hallmark of ‘‘new bronchopulmonary dysplasia (BPD)”

[Clyman RI, 2013]. An early selective treatment approach for closure of a PDA is suggested to trial its effect on BPD, which is the hypothesis of this trial.

Both indomethacin and ibuprofen have been shown to have comparable efficacy in closing PDA. The relative risks of treatment strategies adapted from the Cochrane Collaboration reviews are outlined in this table [Fowlie PW, 2010; Ohlsson A, 2011; Ohlsson A, 2010; Cooke I, 2009].

		Relative risk (95% Confidence interval)						^^
Author	Intervention	Symptomatic PDA	Death before 36 Weeks	BPD at 36 weeks	NEC	Severe IVH	Death / Severe Disability 18–24 Months	Duration of Ventilation (days)
Fowlie PW, *Cochrane Review* 2010, comparison 1	Prophylactic indomethacin	0.44<br>(0.38, 0.50)*	0.82<br>(0.65, 1.03)	1.06<br>(0.92, 1.22)	1.09<br>(0.82, 1.46)	0.66<br>(0.53, 0.82)*	1.02<br>(0.90, 1.15)	-1.83<br>(-5.53, 1.87)
Ohlsson A, *Cochrane Review* 2011, comparison 1	Prophylactic ibuprofen	0.17<br>(0.11, 0.26)*	0.90<br>(0.62, 1.30)	1.04<br>(0.87, 1.25)	1.04<br>(0.63, 1.70)	0.82<br>(0.54, 1.26)	-	1.02<br>(-1.99, 4.03)
Ohlsson A, *Cochrane Review* 2010, comparison 2	Symptomatic PDA (indomethacin vs. ibuprofen)	1.28<br>(0.48, 3.38)	1.12<br>(0.59, 2.11)	1.12<br>(0.77, 1.61)	0.68<br>(0.47, 0.99)*	1.21<br>(0.74, 1.98)	-	-1.96<br>(-4.97, 1.05)
Cooke L, *Cochrane Review* 2009, comparison 1	Early asymptomatic indomethacin	0.36<br>(0.19, 0.68)*	1.32<br>(0.45, 3.86)	0.91<br>(0.62, 1.35)	0.41<br>(0.05, 3.68)	-	-	-5.00<br>(-17.33, 3.34)
Ohlsson A, *Cochrane Review* 2010, comparison 1	Early asymptomatic ibuprofen	0.27<br>(0.12, 0.60)*	0.8<br>(0.34, 1.90)	0.99<br>(0.88, 1.11)	1.00<br>(0.64, 1.55)	1.00<br>(0.47, 2.15)	-	-

*p<0.05
^^ Weighted Mean Differences [WMD] (95% Confidence interval)

5. Trial Objective

5.1. Primary Objective

To determine if selective early treatment of echocardiographically confirmed large PDAs in extremely preterm babies with ibuprofen within 72 hours of birth reduces the incidence of death by 36 weeks of postmenstrual age or moderate or severe bronchopulmonary dysplasia (BPD) at 36 weeks of postmenstrual age.

5.2. Secondary Objectives

To determine if the selective treatment of confirmed large PDAs in extremely preterm babies with ibuprofen within 72 hours of birth results in:

- A reduction in the components of the primary outcome: death by 36 weeks of postmenstrual age; moderate or severe BPD at 36 weeks of postmenstrual age, severity of BPD at 36 weeks of postmenstrual age; other secondary outcomes up to discharge (see Secondary Endpoints);

- Improved health outcomes at 2 years corrected age including survival without moderate or severe neurodevelopmental disability (long-term primary objective) and survival without respiratory morbidity (long-term secondary objective).

An economic evaluation: an economic evaluation will be carried out from the perspective of the health service. It will take the form of a cost-effectiveness analysis presented in terms of cost per major outcome averted. The major outcomes are those of the primary outcome, namely death and moderate or severe BPD by 36 weeks of postmenstrual age. Additional analyses will take place on a range of secondary outcomes and on neurodevelopmental outcomes at 2 years. The incremental cost estimate for statistically significant differences in the pre-specified outcomes in primary and subgroup analyses would be computed.

6. Trial Design

6.1. Summary

This is a multicentre, masked, randomised, placebo-controlled parallel group trial to determine if the treatment of a large PDA with ibuprofen in extremely preterm babies (23^{+0} to 28^{+6} weeks of gestation) improves short and long term health and economic outcomes. The main trial will be preceded by an internal pilot phase which will be used to assess the suitability of trial procedures and likelihood of recruitment targets being achieved.

The entire trial is anticipated to take 97 months to complete and aims to recruit a total of approximately 730 extremely preterm babies.

6.2. Inclusion Criteria

Babies will be considered eligible for inclusion into the trial if they are:

- Born at 23^{+0} to 28^{+6} weeks of gestation
- Less than 72 hours old
- Confirmed by echocardiography as having a large PDA which
  - is at least 1.5 mm in diameter (determined by gain optimised colour Doppler)

  and

  - has unrestrictive pulsatile (left to right) flow (ratio of flow velocity in PDA Maximum (V_{max}) to Minimum (V_{min}) > 2:1) or, growing flow pattern (< 30% right to left), and **no clinical concerns of pulmonary hypertension**

In addition:

- The responsible clinician is uncertain about whether the baby might benefit from treatment to close the PDA
- Written informed consent has been obtained from the parent(s)

6.3. Exclusion Criteria

Babies will be excluded from participation in the trial if they have:

- No realistic prospect of survival
- Severe congenital anomaly
- Clinical or echocardiography suspicion of congenital structural heart disease that contraindicates treatment with ibuprofen
- Other conditions that would contraindicate the use of ibuprofen (active bleeding especially intracranial or gastrointestinal bleeding, coagulopathy, thrombocytopenia (platelet count <50,000), renal failure, life threatening infection, pulmonary hypertension, known or suspected necrotising enterocolitis (NEC))
- Indomethacin, ibuprofen, or paracetamol administration after birth

6.4. Setting

The trial will be conducted in approximately 30 neonatal units (5 units will be involved in the internal pilot phase).

Only units that are in equipoise in the way that they manage PDA, are able and agree to perform echocardiograms within 72 hours of birth to confirm the presence of a large PDA.

6.5. Primary Outcome

The primary outcome is defined as a composite outcome of death by 36 weeks of postmenstrual age, or moderate or severe BPD at 36 weeks of postmenstrual age.

TABLE: Severity-Based Diagnostic Criteria for BPD

Time point of assessment:	36 weeks of postmenstrual age
Therapy with oxygen > 21% and/or respiratory support for ≥ 28 days and the following:	
Mild BPD;	Baby is breathing room air
Moderate BPD;	Baby is in 22–29% oxygen, or 0.01–1.0 L/min
Severe BPD;	$FiO_2 \geq 0.3$, or low flow oxygen ≥ 1.1 L/min, or the baby is receiving any respiratory support (ventilation, CPAP, or high flow oxygen therapy) to achieve saturations of ≥ 91%

The need for oxygen is subjective and hence oxygen dependency will be confirmed using an 'oxygen reduction test'. This is based on the threshold at which the baby is able to maintain oxygen saturations ≥ 91% whilst breathing in air or at a given minimum FiO_2. Babies unable to achieve this will be considered to be oxygen dependent. This test will only apply to those babies whose oxygen requirements are < 0.3, or low flow oxygen < 1.1 L/min, and who have not received any additional respiratory support in the previous 24 hours. Babies outside of this will not be tested, but their oxygen requirements will be captured on the relevant case report form.

6.5.1. *Oxygen Reduction Test*

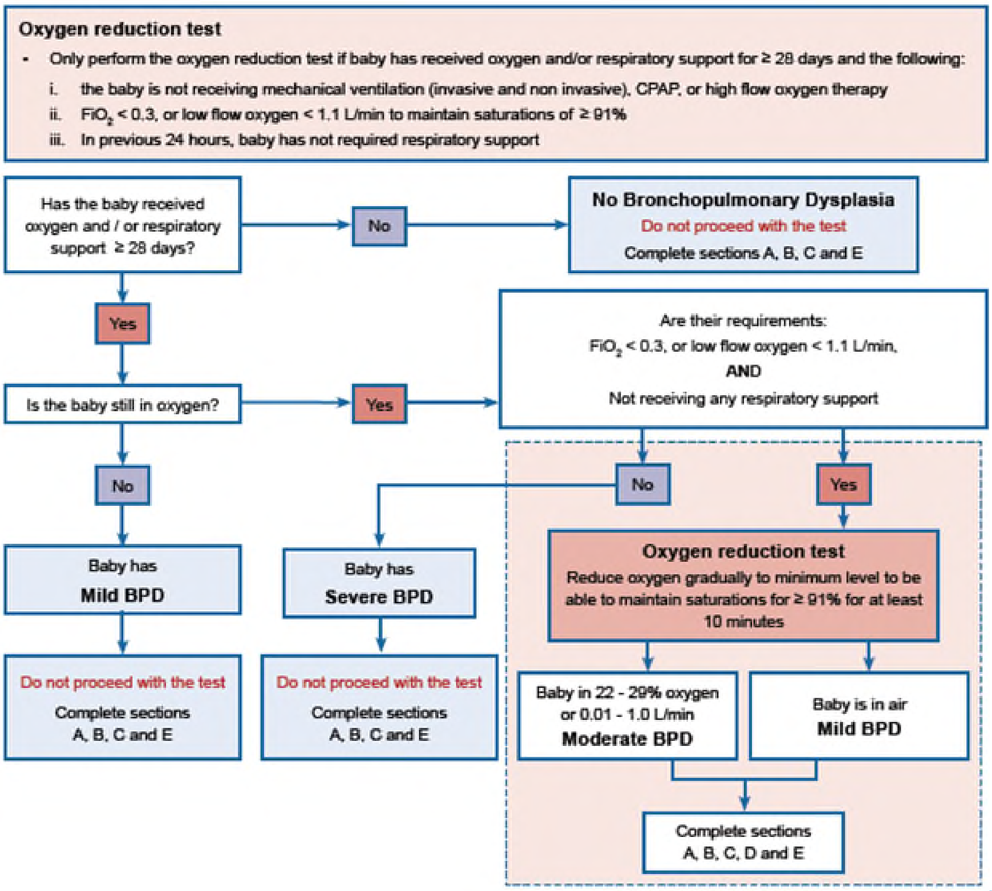

6.6. Secondary Outcomes

Secondary outcomes are divided into short and long term outcomes.

Short term outcomes

- Death by 36 weeks of postmenstrual age
- Moderate or severe BPD at 36 weeks of postmenstrual age
- Severity of BPD at 36 weeks of postmenstrual age (see table in Section 6.5)

Incidence or duration of the following up to discharge:

- Severe intraventricular haemorrhage (IVH) (grade III/IV with ventricular dilatation or intraparenchymal abnormality)
- Cystic periventricular leukomalacia (PVL)
- Non-cystic PVL
- Hydrocephalus
- Babies treated for Retinopathy of prematurity (ROP)
- Significant pulmonary haemorrhage (fresh blood in endotracheal tube with increase in respiratory support)
- Treated for pulmonary hypertension with pulmonary vasodilator
- NEC definitive and/or complicated (Bell stage II and above) confirmed by radiology and/or histopathology
- NEC requiring surgery
- Gastrointestinal bleeding (leading to investigation or clinical treatment) within 7 days of the first dose of trial drug administration
- Spontaneous intestinal perforation
- Closed or non-significant PDA (<1.5 mm) at around 3 weeks of age (range of 18 – 24 days), confirmed by ECHO
- PDA ≥ 1.5 mm at around 3 weeks' (range of 18 – 24 days)
- Medical open-label treatment of a symptomatic PDA with a COX inhibitor
- Open-label treatment of a symptomatic PDA by surgical treatment
- Administration and duration of inotropic support
- Total duration of respiratory support
  - a) Invasive ventilation through an endotracheal tube
  - b) Non-invasive support through, nasal CPAP, nasal ventilation, humidified high flow nasal cannula therapy, or low flow oxygen ≥ 1.1L/min

- Discharge home on oxygen
- Duration of initial hospitalisation (birth to discharge home)
- Postnatal steroid use for chronic lung disease
- Tolerance of ibuprofen treatment within the foreseeable SAE reporting range, described in the protocol, section 9.1.4
- Weight gain: a change in z score between birth and discharge (or death if sooner)
- Head circumference: a change in head size z score between randomisation and discharge (or death if sooner)

Long Term Outcomes assessed at 2 years of age corrected for prematurity

- Survival without moderate or severe neurodevelopmental disability (main long term outcome)
- Survival
- Individual components of survival without moderate or severe neurodevelopmental disability (in the four domains of motor, cognitive, hearing and visual function). Cognitive disability will be assessed by determining the standardised non-verbal cognitive subscale and language subscale scores obtained through the Parent Report of Cognitive Abilities-Revised (PARCA-R) assessment. The PARCA-R assessment will be adapted to include questions to assess gross motor, hearing and visual function.
- Survival without respiratory morbidity. Respiratory morbidity will be assessed by the need for oxygen or respiratory support; presence of persistent cough and/or wheeze; need for regular treatment for respiratory illness; unscheduled attendances at hospital/GP; readmission to hospital for respiratory problems
- Duration of oxygen supplementation from randomisation

A cost-effectiveness analysis will be conducted of deaths and BPD events avoided and national health services used up to 2 years of age corrected for prematurity.

6.7. Process Outcomes

Process Outcomes will be the following;

- Number of doses of trial medication received
- Adherence to protocol (e.g. protocol violations, incidence of non-symptomatic open-label treatment etc.)
- Study withdrawals

7. Trial Procedures

7.1. Trial Assessments

Procedure	Baby Hospitalisation					
	Screening [1]	Trial Entry and Treatment (days 1–3)	Up to 7 days after trial medication	3 weeks of Age	36 weeks of PMA[10]	Discharge
Demography[9]		✓				✓
Echocardiogram/Colour Doppler[8]	✓			✓		
Confirmation of Eligibility	✓					
Consent		✓				
Randomisation[2]		✓				
Ibuprofen/Placebo Dosing[3]		✓				
IVH / PVL ultrasound scans[10]			✓		✓	
NEC						✓
Oxygen Reduction Test					✓	
SAEs[4]		✓	✓			
Concomitant Medication[5]	✓	✓		✓	✓	✓

Infant at 2 Years Corrected Age[6,7]	
Demography[9]	✓
Visual Assessment[6]	✓
Hearing Assessment[6]	✓
Motor Assessment[6]	✓
Respiratory Assessment[7]	✓

1 Screening assessments to be completed sufficiently in advance to enable randomisation and dosing within 72 hours of birth. If consent cannot be obtained before echocardiographic evaluation for eligibility, echocardiographic assessment should continue and consent obtained when possible if a baby is deemed eligible.

2 Randomisation to be completed sufficiently in advance to enable dosing within 72 hours of birth.

3 Initial trial drug administrations to be given soon after randomisation, after 6 hours of age and within 72 hours of birth. Subsequent doses to be administered 24 hours after the initial dose.

4 Only adverse events which are serious will be recorded from first dose until 7 days after trial medication. Only unforeseeable SAEs will be reported.

5 Concomitant medications to be recorded only in relation to unforeseeable SAEs. In the event of an unforeseeable SAE all concomitant medication, including medication given to the baby's mother, 7 days prior to the onset of the event to the time of its resolution must be recorded on the SAE form.

6 Gross Motor, Cognitive, visual and hearing function will be assessed using the PARCA-R questionnaire, expanded to include questions to assess visual and hearing function.

7 Respiratory assessments will be performed using a separate validated questionnaire. There will be no requirement for the infants to be assessed for respiratory and/or other neurodevelopmental functions by medically qualified personnel.

8 An echocardiogram scan will be performed when the baby reaches around 3 weeks of age (range of 18 – 24 days) or at hospital discharge if discharged earlier.

9 Demography and medications will be assessed through the PARCA-R and other questionnaires.

10 If a baby transfers from the recruiting site to a continuing care site for on-going care details of any scan would be helpful.

7.2. Structure and Duration of the Trial

The total duration of recruitment into this trial will consist of an internal pilot phase (9-month recruitment period) and main trial (36-month recruitment period). The aim is to recruit approximately 730 babies from participating neonatal units across the UK in about 65 months (internal pilot and main trial).

For the main trial, the recruitment period is based on an approximate yearly admittance of 2,000 babies born between 23 and 28 weeks of gestation to approximately 30 participating neonatal units and the assumption that about 46% of these babies will have a large PDA [Stoll et al, 2010]. This would result in approximately 900 babies being eligible for inclusion in this trial. Assuming a conservative uptake rate of 20−30% this would equate to around 250 babies being enrolled per year (1 per unit per month).

The trial will consist of an internal pilot phase, run over a period of 13 months (including a 4-month trial set-up period), in five level 3 neonatal units to test whether the current trial design and associated procedures will allow overall recruitment targets to be achieved. Projections suggest that around 30 babies should be recruited in that time. Data collected from the internal pilot phase of the trial will be included in the final analysis.

The decision to progress to the main trial using the current design will be made in consultation with the Trial Steering Committee (TSC) and funder. Stop/go criteria upon which a decision will be made will be established prior to the start of the internal pilot phase. Should a decision be made not to progress to the main phase, a report on the internal pilot phase will be submitted for publication according to the publication policy.

All enrolled babies will be followed up at 2 years of age corrected for prematurity. Thus the duration of trial participation will be up to 28 months (2 years corrected age). Where we have been unable to contact families within this timeframe we will attempt to collect information about infants in the trial until the end of the funding. Further longer term follow-up at primary school age may be considered but will require separate funding. This may be undertaken as an amendment to this trial or as a separate application depending on the circumstances at the time.

7.3. Initial Eligibility Assessment

Extremely preterm babies potentially suitable for the trial will be identified by the healthcare team within the neonatal unit. Information about the trial can be provided antenatally or soon after birth. Parent(s) should be approached for consent after birth followed by echocardiographic evaluation for eligibility. However, if consent cannot be obtained echocardiographic assessment should continue and consent obtained when possible if a baby is deemed eligible.

The initial echocardiogram and Doppler assessment will incorporate:

- Size of the PDA and flow pattern according to standard trial methodology
- Size of the PDA will be determined at the site of maximum constriction (minimum diameter) using gain optimisation typically at the pulmonary end by determining the average of 3 separate clips
- If the size of the PDA is at least 1.5 mm, flow pattern will be determined by placing the pulse gate in the PDA while adjusting the velocity scale to its highest setting. If the shunt direction is >1/3 duration of a cycle being right to left, then a rescan will be attempted after a few hours.

Please refer to the ECHO workbook for the Baby-OSCAR Trial.
If the echocardiogram findings raise concerns about possibility or diagnosis of congenital heart disease, a referral will be made to a paediatric cardiologist as per clinician discretion.

7.4. Echocardiograms

Echocardiograms are performed as part of the normal care of preterm babies. However, clinicians will be required to perform an echocardiogram within 72 hours of birth, at around 3 weeks of age (range of 18 – 24 days) or at discharge from the neonatal unit if discharged before this time.

Echocardiogram scans will be reviewed by a qualified clinician, who is not involved in recruiting for the trial, to assess consistency between clinicians. All babies recruited to the internal pilot phase and a randomly selected sample from the main trial, equating to 10% of echocardiogram scans used to confirm trial eligibility, will be reviewed. Principal Investigators will be informed of the review findings.

Training will be provided during the trial to minimise any variations in practice. Any difference in measurements between the site investigator and reviewer will be documented to aid with on-going training. Details of both the echocardiogram procedures to be followed and the process for submitting scans for independent review will be described in a separate handbook.

7.5. Informed Consent

Written informed consent will be sought from parent(s) after they have been given a full verbal and written explanation of the trial. Written explanation will be given via the Parent Information Leaflet. Parent(s) who do not speak English will only be approached if an adult interpreter is available. Relatives will not interpret.

Written informed parental consent will be obtained by means of a dated parental signature and the signature of the person who obtained informed consent; this will be the Principal Investigator (PI) or healthcare/research professional with delegated authority. A copy of the signed informed consent form (ICF) will be given to the parent(s). A further copy will be retained in the baby's medical notes, a copy will be retained by the PI and the original will be sent to the co-ordinating Centre in Oxford.

7.6. Randomisation

Treatment allocation of ibuprofen or placebo will be in a ratio of 1:1 and masked such that the allocation will not be known by clinicians, the baby's family or the trial outcome assessors.

Randomisation will be managed via a secure web-based randomisation facility hosted by the NPEU CTU with telephone back-up available at all times (24/7, 365 days a year). The randomisation program will use a minimisation algorithm to ensure balance between the groups with respect to the size of the PDA, gestational age at birth, age at randomisation, sex, trial site, multiple births, mode of respiratory support at randomisation (1) invasive ventilation (by an endotracheal tube); or (2) non-invasive respiratory support through, nasal CPAP, nasal ventilation, humidified high flow nasal cannula therapy or, low flow oxygen ≥ 1.1L/min; or (3) receiving no mechanical ventilation, or pressure support (in room air, or low flow oxygen <1.1 L/min, or ambient oxygen) and receiving inotropes or not at the time of randomisation. Babies of multiple births will be randomised individually.

The Senior Trials Programmer at the NPEU CTU will write the randomisation program and hold the treatment allocation codes. If necessary, the code may be broken for a single baby at the request of the site PI or clinician in charge of the baby. See Section 8.6 for the procedure for unmasking treatment allocation.

7.7. Concomitant Medications

Concomitant medication given to a baby will be recorded in the event that an unforeseeable serious adverse event is reported for that baby. If such an event is reported, all concomitant medication given 7 days prior to onset of the event, including medication given to the baby's mother, if within the 7 days is prior to the birth, up to its resolution will be detailed on the SAE form provided for the trial.

7.8. Permitted and Non-Permitted Medications

All prescribed medications deemed necessary to provide adequate supportive care to the baby, are permitted at any stage during the trial period. However, open treatment with indomethacin or ibuprofen or other non-steroidal anti-inflammatory drugs (NSAIDs) should be avoided unless the criteria for open-label treatment (defined in Section 8.4) are met.

As a NSAID, ibuprofen may interact with the following medicinal products:

- Diuretics – ibuprofen may reduce the effect of diuretics; diuretics can increase the risk of nephrotoxicity of NSAIDs in dehydrated patients
- Anticoagulants – ibuprofen may increase the effect of anticoagulants and enhance the risk of bleeding
- Corticosteroids – ibuprofen may increase the risk of gastrointestinal bleeding
- Nitric oxide – since ibuprofen also inhibits platelet function, combining the drugs may in theory increase the risk of bleeding
- NSAIDs – the concomitant use of more than one NSAID should be avoided because of the increased risk of adverse reactions.

The concomitant administration of other medication is not restricted but should be closely monitored for an interaction by the treating clinician.

7.8.1. Supportive Care of Enrolled Babies

The management of babies including ventilator management and fluid therapy during intensive or high dependency care will be guided by the European Consensus Guidelines for Management of Respiratory Distress Syndrome (RDS) in preterm babies. An effort will be made to minimise the differences in treatment practices between sites through training on the guidelines.

7.9. Stopping Trial Medication

The intervention may have to be (temporarily) stopped if the baby develops any adverse effects necessitating stoppage. For example, if anuria, marked oliguria (<0.6 ml/kg/hr), or clinically significant bleeding is evident at the scheduled time of the second or third dose, no additional dosage should be given until laboratory results indicate that renal function has returned to normal, or bleeding has stopped. In the event of GI perforation discontinue trial medication.

At all stages it will be made clear to the parent(s) that they remain free to withdraw their baby from the trial at any time without the need to provide any reason or explanation. Parent(s) will be made aware that a decision to withdraw their baby will have no impact on any aspect of

their baby's continuing care. If parent(s) choose to withdraw their baby from trial participation, permission will be sought to complete data collection and use data up to the point of withdrawal from the trial.

A baby may also be withdrawn from the trial, if deemed by the Principal Investigator to be in their best interests.

7.10. Out of Hours

In the case of urgent out-of-hours queries please phone 0800 138 5451. When you call this Freephone number you will go through to Message Direct who will ask for your name, the hospital you are calling from, your full phone number and the name of the trial (Baby-OSCAR) before they are able to address your urgent query.
The contact details for both the NPEU CTU and Message Direct are as follows:

Office hours	NPEU CTU:	01865 617 965
Out of office and weekends	Message Direct:	0800 138 5451

Details of contact numbers will also be filed in the Investigator Site File.

7.11. Breakages and Spoilt Ampoules

Packs of trial medication must only be administered to the baby to whom it was allocated. If any ampoules break or are spoilt, discard the ampoule(s) (recording it on the IMP Accountability Log), log onto the website and re-allocate another trial medication pack for the remaining doses if the initial allocated pack does not have sufficient doses.

7.12. End of Trial

The end of trial will be defined as the date when the trial database is locked. An End of Trial Declaration will be made to the Medicines and Healthcare Products Regulatory Agency (MHRA) and approving Research Ethics Committee (REC).

7.13. Early Trial Cessation

A decision may be made by the Trial Steering Committee (TSC) to stop the trial early following a recommendation from the Data Monitoring Committee (DMC), on review of interim trial data, or evidence from other relevant studies becoming available. Guidelines for the early cessation of the trial will be agreed with the DMC and documented in the DMC Charter.

7.14. Remuneration

No financial or material incentive or other form of compensation will be given to babies or their parent(s) as a result of taking part in this trial.

8. Investigational Medicinal Product (IMP)

8.1. Dosing and Administration

Ibuprofen will be supplied as a clear sterile solution at a concentration of 5 mg/ml in ampoules. Cartons containing four 2 ml single use ampoules will be provided. Each carton will be labelled with a unique code and in compliance with the guidance given in Annexe 13 of the European Commission's guidelines for Good Manufacturing Practice.

An initial loading dose of 10 mg/kg (2 ml/kg) of ibuprofen will be administered, followed by two 5 mg/kg (1 ml/kg) doses at 24 and 48 hours after the initial dose. Doses are to be calculated on the birth weight of the baby and preferably administered undiluted. If required, the IMP can be diluted to appropriate volume with 5% glucose or 0.9% Sodium Chloride. Each dose is to be given as a short intravenous infusion over 15 minutes. All 3 doses will be given unless there are adverse effects necessitating stoppage, as referenced in Section 7.9. Placebo will be supplied as a clear sterile solution of 0.9% Sodium Chloride for injection. Cartons identical to those for ibuprofen, each containing four identical single use ampoules will be provided. Volume of IMP to be withdrawn from the ampoule will be calculated following the calculations for ibuprofen dosing.

Following randomisation, first dose should be administered soon after randomisation, after 6 hours of age and within 72 hours of birth. The recommended storage will be in line with the Summary of Product Characteristics (SmPC) and once the ampoule is opened the drug must be used immediately.

8.2. Distribution

Sufficient supplies of IMP will be provided to each site. Distribution and use of IMP will be tracked by the staff at the NPEU, using a 'pack management system' and additional supplies provided as and when needed.

8.3. Accountability

Trial drug packs will be allocated by the central randomisation system and will be recorded by the NPEU CTU and the dispensing pharmacy. Detailed accountability records will be maintained to document which pack of medication is allocated to which baby. Site staff will be required to write the baby's trial number and initials on the trial pack allocated. Part used packs

will be kept separate from unused packs.

8.4. Open-label Treatment

If the clinical condition of a baby warrants intervention, open-label treatment can be given to close the PDA (medical or surgical). The following criteria, however, have been devised to limit and rationalise the use of open-label treatment but it is recognised that clinicians may need to override this guidance in the best interests of the baby. Clinical responsibility for the care of the baby will remain fully with the neonatal clinical team irrespective of the trial.

Open-label treatment (both medical and surgical) is permitted within the protocol if the following minimum criteria are met and other medical management strategies have been tried. Surgical treatment however should only be considered if the PDA remains persistently large after one course of treatment with a COX inhibitor or in circumstances where medical treatment may be contraindicated or time does not permit medical open-label treatment first.

1. Inability to wean on ventilator (ventilated for at least 7 days continuously) and any of: inability to wean oxygen; persistent hypotension; pulmonary haemorrhage; signs of cardiac failure

AND

2. Echocardiographic findings of a large PDA (PDA ≥ 2.0 mm with pulsatile flow)

AND

3. Echocardiographic findings of hyperdynamic circulation or ductal steal (refer to Baby-OSCAR ECHO workbook).

All open-label treatment will be administered in an open fashion.

A persistent open PDA requiring open-label treatment (medical or surgical) should be reported to the Trial Co-ordinating centre using Form 5: Open Treatment of PDA.

8.5. Masking of Trial Medication

Ibuprofen and placebo will be indistinguishable from each other. To maintain masking, each baby will be issued a unique trial medication pack ID that will correspond to a carton ID.

8.6. Emergency Procedure for Unmasking / Envelopes

In the event of an emergency, a baby may be unmasked by the clinician at the recruiting site by logging in to the randomisation website using a single-use access code provided in a sealed envelope. The reason for unmasking must be recorded. Clinicians are reminded to exercise discretion when the allocation has been unmasked.

Clinicians carrying out emergency unmasking must be satisfied that it is a genuine emergency and that knowledge of the treatment allocation (either ibuprofen or placebo) is needed to guide

the appropriate clinical management of the baby. In some cases, this may be achieved without unmasking, by treating the baby as if they have received ibuprofen.

Where the baby has been transferred out of the recruiting site for onward care, the treating health care professional should contact the PI or any clinician on the delegation log at the recruiting site to unmask.

As it is best practice to not unmask babies until any follow-up is completed, all other requests for unmasking must be made in writing to the NPEU CTU, who along with Chief Investigator will consider the request.

9. Safety Reporting

9.1. Definitions

9.1.1. Adverse Event (AE)

An adverse event is any untoward medical occurrence in a participant administered a medicinal product, which does not necessarily have to have a causal relationship with this treatment. An AE can therefore be any unfavourable and unintended sign (including an abnormal laboratory finding), symptom or disease temporally associated with the use of the trial medication, whether or not considered related to the trial medication.

9.1.2. Adverse Reaction (AR)

All untoward and unintended responses to a medicinal product related to any dose. The phrase "response to a medicinal product" means that a causal relationship between trial medication and an AE is at least a reasonable possibility, i.e. the relationship cannot be ruled out.

9.1.3. Serious Adverse Event (SAE)

A serious adverse event is any untoward medical occurrence that:

- Results in death
- Is life-threatening
- Requires participant hospitalisation or prolongation of existing hospitalisation
- Results in persistent or significant disability/incapacity
- Is a congenital anomaly/birth defect
- Is an important medical event

The term 'severe' is often used to describe the intensity (severity) of a specific event; the event itself, however, may be of relatively minor medical significance. This is not the same as 'serious', which is based on participant/event outcome or action criteria usually associated with events that pose a threat to a participant's life or functioning.

The term 'life-threatening' in the definition of serious refers to an event in which the participant was at risk of death at the time of the event; it does not refer to an event that hypothetically might have caused death if it were more severe.

Medical and scientific judgement should be exercised in deciding whether an adverse event is serious in other situations.

9.1.4. Foreseeable Serious Adverse Events

Foreseeable SAEs are those events which are foreseen in the patient population or as a result of the routine care/treatment of a patient.

The following serious adverse events are a foreseeable occurrence in this population of preterm babies and as such do not require reporting as SAEs:

- Anaemia requiring transfusion
- Clinically significant intracranial abnormality on cranial ultrasound scan – intracranial haemorrhage or white matter injury
- Coagulopathy requiring treatment
- Culture proven sepsis
- Death (unless unforeseeable in this population)
- Fluid retention
- Gastrointestinal bleeding
- Haematuria
- Haemothorax
- High blood creatinine level (defined as >100 µmol/L)
- Hyperbilirubinemia necessitating exchange transfusion
- Hyperglycaemia
- Hypoglycaemia
- Hypotension treated with inotropes
- Impaired renal function (urine output <0.5 ml/kg/hour, and or serum creatinine > 100 µmol/L)
- Low serum sodium level/hyponatremia (defined as sodium <130 mmol/L)
- Necrotising enterocolitis
- Neutropenia (defined as <1.0 mmol/L)
- Pneumothorax requiring treatment
- Pulmonary hypertension requiring treatment with pulmonary vasodilator
- Respiratory failure
- Seizures requiring treatment
- Significant pulmonary haemorrhage
- Spontaneous intestinal perforation
- Thrombocytopenia

Hypoglycaemia and hyperglycaemia are commonly encountered in preterm babies born below 29 weeks' gestation receiving neonatal intensive care. As per the product characteristics, the risk of hypo or hyperglycaemia does not increase with the use of Pedea® (IMP in Baby-OSCAR trial). Hence, we will include hypoglycaemia and hyperglycaemia as an expected SAE in the trial participants, but will not report data on its occurrence in the trial study groups while babies receive standard neonatal intensive care.

Given that the babies in the trial are extremely pre-term, and the expectation is that they will be in various stages of respiratory failure, the occurrence of respiratory failure will not be reported in relation to trial medication.

9.1.5. Unforeseeable Serious Adverse Events

An unforeseeable SAE is any event that meets the definition of a SAE and is not detailed in the list above as foreseeable. These events should be reported on the trial SAE form provided following the procedures detailed in Section 9.2.1.

9.1.6. Serious Adverse Reaction (SAR)

A serious adverse reaction is a SAE which is considered to have been caused by the administration of trial medication. For a SAE to be considered as a reaction there must be a reasonable probability that it was related to the administration of IMP.

9.1.7. Suspected Unexpected Serious Adverse Reaction (SUSAR)

This is a SAR, the nature or severity of which is not consistent with the known safety profile of the trial medication (e.g. Investigator's Brochure for an unapproved IMP or Summary of Product Characteristics for an approved product). The Reference Safety Information for Sodium Chloride of ibuprofen is contained within the SmPC which will be used to assess the expectedness of adverse events.

9.1.8. Causality

The relationship of each adverse event to the trial medication must be determined by a medically qualified individual according to the following definitions:

Unrelated; where an event is not considered to be related to the IMP;

Possibly; although a relationship to the IMP cannot be completely ruled out, the nature of the event, the underlying disease, concomitant medication or temporal relationship make other explanations possible;

Probably; the temporal relationship and absence of a more likely explanation suggest the

event could be related to the IMP;

Definitely; the known effects of the IMP, its therapeutic class or based on challenge testing suggest that the IMP is the most likely cause.

All SAEs as discussed in Section 9.2.1 labelled possibly, probably, or definitely will be considered as related to the IMP.

9.1.9. Assessment of Safety

During the course of the trial, safety data will be reviewed by the Data Monitoring Committee (DMC). This will include safety data for SAEs as stated in Section 9.2.1 as well as Section 9.1.4 and 9.1.5. The DMC will, if appropriate, make recommendations regarding continuance of the trial or modification of the trial protocol. The TSC will have ultimate responsibility for deciding whether the trial should be stopped on safety grounds.

9.2. Reporting Procedures

9.2.1. AE/SAE Reporting

Causality of adverse events reported in the preterm new-born is difficult to assess since they may be related to the haemodynamic consequences of the patent ductus arteriosus as well as to direct effects of ibuprofen. In addition to this, high incidences of adverse events are foreseeable due to the nature of the patient population and the routine care/treatment. Consequently, only those adverse events identified as serious will be recorded for the trial.

Safety reporting as described in this section for the baby will be monitored from first dose until 7 days after trial medication. Unforeseeable Serious Adverse Events will be reported to the NPEU CTU within 24 hours of staff at the site becoming aware of the event. Details will be recorded on a SAE form (filed in the Investigator Site File) and the form faxed or emailed back to the NPEU CTU. If this is not possible, the unforeseeable SAE may be reported by telephone and the SAE form completed by staff at the NPEU CTU. Follow-up information should be reported on a new SAE form and this forwarded to the NPEU CTU by fax or email.

NPEU will review the report, request any additional information and ensure it is assessed by the CI or his delegate within the reporting timeframe. It will also be reviewed at the next DMC meeting. The CI will inform all Principal Investigators of relevant information that could adversely affect the safety of the participants.

9.2.2. SUSAR Reporting

SUSARs will be reported to the MHRA and the approving Research Ethics Committee (REC) within 7 days if the event resulted in death or was life-threatening, and within 15 days for all

other SUSARs. In addition, a copy of the SAE form corresponding to the event will be forwarded to the Chair of the DMC. The Chair will also be provided with details of the baby's treatment allocation.

9.2.3. Development Safety Update Report (DSUR)

In addition to the expedited reporting detailed above, the CI will submit a Development Safety Update Report (DSUR) once a year throughout the duration of the trial to the MHRA and REC.

10. Statistics and Analysis

10.1. Sample Size

Evidence from the TIPP trial suggests that the risk of death or BPD in extremely low birth weight babies at 36 weeks of postmenstrual age allocated placebo is 52% (95% CI 48% to 56%) [Schmidt et al, 2001]. However, this trial investigated the effect of prophylactic treatment and included all babies weighing 500−999g. More recent information using data derived from the latest report of Neonatal Survey Database from the Trent region (2010) provides an approximate rate of death or BPD by 36 weeks of postmenstrual age of 53% for all babies admitted to the neonatal unit. These babies would have been treated according to clinical judgement and therefore a proportion of them would have been treated with ibuprofen. Given that the risk of death or BPD in babies with a large PDA is inherently higher, it is estimated that the risk in this group is 60%.

Su et al (2008) compared ibuprofen to indomethacin in babies ≤ 28 weeks of gestation having a PDA who were less than 24 hours old. The combined outcome of death within 30 days or BPD at 36 weeks of postmenstrual age was observed to be 42% (95% CI 29% to 55%).

It is therefore expected, given that babies will be enrolled up to 72 hours after birth, that the treatment group incidence of death/BPD at 36 weeks of postmenstrual age will be approximately 48% in the intervention arm. This would imply an absolute risk reduction of 12% (60% to 48%) in the primary outcome of the trial for babies randomised to treatment compared to placebo, which is considered a clinically important difference.

Some babies will require open-label treatment in either the treatment or placebo arm. As open-label treatment should be limited to symptomatic babies meeting only defined criteria, it is considered to have minimal or no effect on the primary outcome. Thus adjustment of the sample size for open-label treatment is not considered necessary.

The following graph depicts a sample size curve for the primary outcome of the trial of death or BPD by 36 weeks of postmenstrual age, assuming 90% power, a two-sided 5% significance

level and a 60% control group event rate for the primary outcome.

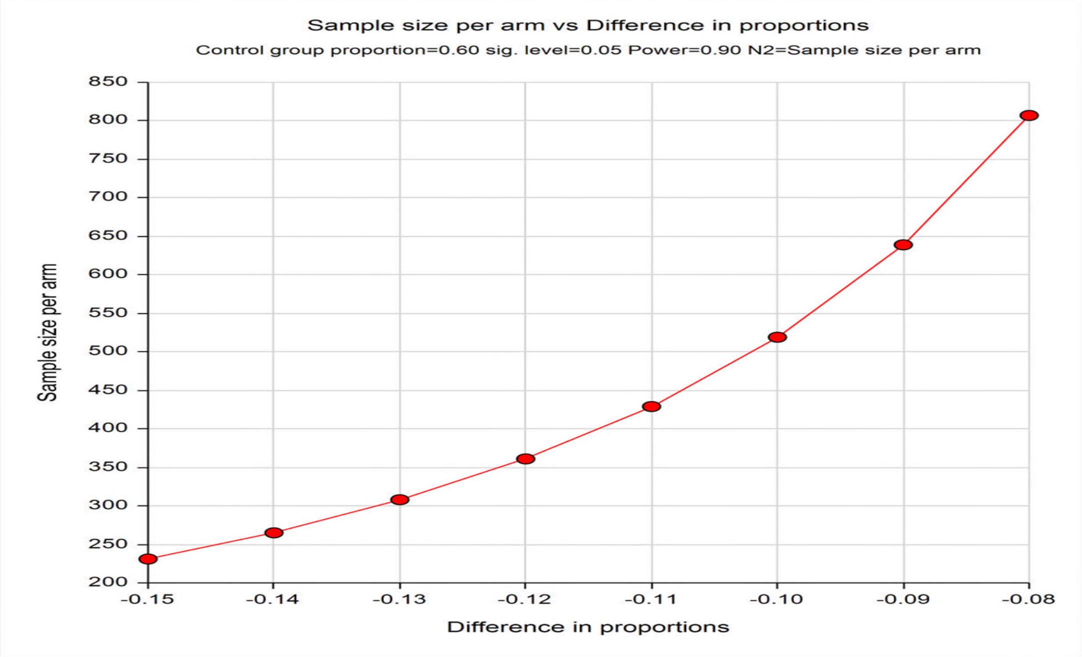

This table summarises this information and allows for 1% loss to follow-up in the primary outcome. Minimal loss to follow-up is expected for the primary outcome since it is a short term outcome and recorded whilst the baby is in hospital.

Control group event rate	Active Rx group event rate	Absolute risk reduction	Relative risk reduction	Approximate total sample size required
60%	47%	13%	22%	620
**60%**	**48%**	**12%**	**20%**	**730**
60%	49%	11%	18%	870

Thus a sample size of approximately 730 babies in total (365 per arm) would be required to detect an absolute risk reduction of 12% (power 90%, 2-sided significance level of 5%) from a control group event rate of 60% to a treatment group event rate of 48%, assuming 1% lost to follow-up.

Regarding outcomes at 2 years corrected age, assuming the risk of a child dying before two years of age is 10%, questionnaires will be sent out to around 660 parents of surviving children. Assuming an attrition rate of 20% reduces the sample size to around 530. The proportion of infants surviving to 2 years without moderate or severe neurodevelopmental disability in the control group is expected to be 55% [Mangham et al, 2009]. With outcome data available on a total sample size of around 600 (including deaths) the trial will have an 80% power to detect an increase in survival without moderate or severe neurodevelopmental disability of 11% from 55% to 66% and 90% to detect an increase of 13% from 55% to 68%.

10.2. Assessment of Outcomes

Short term outcome data will consist of routine clinical and laboratory assessments. The timing and methods for assessing and determining the short outcomes are consistent with those routinely performed for babies in intensive/high dependency care.

Long term outcomes will be assessed using validated parent report questionnaires. Standardised measures (PARCA-R) and additional items designed to elicit key information regarding visual and hearing impairment, respiratory morbidity and health economic outcomes will be combined into a single trial-specific questionnaire, which will be posted to parents one week prior to the date on which the child would have turned 2 years old if they had been born at term (40 weeks'). The Health and Social Care Information Centre (HSCIC) (or a named derivative) will be used to alert the Trial Co-ordinating Centre of deaths prior to follow-up.

To estimate the costs associated with the echocardiography/Doppler screening within 72 hours of birth in preterm babies, the following will be monitored;

- Staff resource used to carry out echocardiogram/Doppler screening within 72 hours compared with current practice
- The time, resource and unit costs associated with medication or tests and procedures as a result of earlier screening
- Requirement for neonatal medication
- Duration of stay in neonatal intensive care and inpatient days
- Admissions after discharge.

Unit costs will be given to each resource item to determine an overall cost per baby. Primary cost data for many of the resources will be collected from participating hospital sites. Where possible other costs data, such as cost of a clinician's time to perform an assessment will be collected from hospital finance departments. Most cost data are already available in published sources. For example, a study to investigate the costs of different levels of neonatal intensive care has already been carried out and other cost studies with relevant costs and costs associated with preterm delivery are available to supplement these [Petrou et al, 2003; Roberts et al, 2012].

10.3. Statistical Analysis

10.3.1. Primary Analysis Population

Babies will be analysed in the groups to which they are randomly assigned, comparing the outcome of all babies allocated to ibuprofen with all those allocated to placebo, regardless of deviation from the protocol or treatment received (referred to as the Intention to Treat (ITT) population).

10.3.2. Statistical Methods

Baseline characteristics and outcomes will be summarised with counts and percentages for categorical variables, means and standard deviations for normally distributed continuous variables, or median and interquartile range for other non-normally distributed continuous or time-to-event variables.

For binary outcomes, risk ratios and confidence intervals will be calculated using log binomial regression, or if a model fails to converge a Poisson regression model with a robust variance estimator will be used. Continuous outcomes will be analysed using linear regression models, with mean differences and confidence intervals presented for approximately normally distributed outcomes. Skewed continuous outcomes will be analysed using quantile regression models, with median differences and confidence intervals presented. Time-to-event outcomes will be analysed using Cox regression and hazard ratios with confidence intervals will be presented.

Analyses will be adjusted for all minimisation factors and the correlation between siblings from multiple births where possible. Both crude and adjusted effect estimates will be presented, but the primary inference will be based on the adjusted estimates.

Due to the multiple number of short term outcomes, and correlation between some outcomes, statistical inference will be restricted to a predefined list of tested outcomes. Summary data by trial arm will be provided for all other outcomes, but statistical tests (or the calculation of confidence intervals) will not be performed.

Long-term outcomes

The main long-term outcome assessed at two years of age corrected for prematurity is survival without moderate or severe neurodevelopmental disability (defined as moderate or severe disability of motor, cognitive, hearing or visual function). Cognitive disability will be assessed by determining the standardised non-verbal cognitive subscale and language subscale scores obtained through the PARCA-R assessment (with scores of 54 or less in either classed as severe disability, and scores of 55 to 69 in either classed as moderate disability).

Standardised PARCA-R scores cannot be calculated for infants whose questionnaires were completed outside of 23.5 to 27.5 months of age corrected for prematurity, although their raw scores may be available. It could therefore be assumed that these standardised scores will be missing at random. A multiple imputation analysis will be performed for this long-term outcome, imputing standardised scores for this group of infants. (see section 10.3.5). Estimates from this multiple

imputation analysis will be presented as the primary inference. The other components of neurodevelopmental disability (motor, hearing and visual function) are not restricted by age in this way, so it is not expected that imputation for these will be required.

10.3.3. Pre-specified Subgroup Analysis

.

Pre-specified subgroup analysis will use the statistical test of interaction (or test for trend) and where appropriate, results will be presented as risk ratios with confidence intervals.

Pre-specified subgroups on the primary outcome and its components will be based on:

- gestational age at birth
- size of the PDA
- mode of respiratory support at randomisation.

A further pre-specified subgroup analysis of NEC Bell stage II and above will be conducted by size of the PDA.

10.3.4. Level of Statistical Significance

95% confidence intervals will be used for all pre-specified outcome comparisons including subgroup analysis. Due to the large number of secondary outcomes, a pre-specified list of tested and untested outcomes will be included in the Statistical Analysis Plan.

10.3.5. Dealing with Missing Data

Missing data as a result of babies being lost to follow-up is expected to be minimal for short term outcomes. For 2 year outcomes, all reasonable measures will be taken to minimise loss to follow-up which is expected to be no more than 20% (excluding deaths after randomisation). Babies for whom no 2 year follow-up data are received will be compared to babies with 2 year data on demographic and clinical characteristics, as well as short term outcomes, to assess generalisability.

For the PARCA-R non-verbal and language subscale scores (to measure cognitive disability), strategies detailed in the PARCA-R manual [Johnson et al, 2019] will be employed where items are missing.

Standardised PARCA-R scores can only be calculated for questionnaires completed within 23.5 to 27.5 months of age corrected for prematurity (inclusive). Scores outside this age range

will be imputed using multiple imputation based on the raw scores and other baseline characteristics [Enders, 2010], and this will be reported as the primary inference. A further sensitivity analysis will be conducted, excluding infants whose assessments were completed outside this range. Assessments completed outside this range will be treated as missing.

10.4. Economic Analysis

Health economic outcomes will take the form of a cost-effectiveness analysis of deaths and any moderate or severe BPD at 36 weeks of postmenstrual age avoided, as well as analysis of the cost implications of secondary outcomes. Analysis will be from the perspective of the NHS, so only direct NHS costs will be collected.

To determine economic outcomes, a within-trial analysis will be conducted but a model based analysis beyond the end point of the trial will also be considered.

The within-trial analysis will be based on two clinical endpoints. The first within-trial analysis will be based on the composite clinical outcome of death avoided and/or case of moderate or severe BPD eliminated at 36 weeks of postmenstrual age (this outcome can also be interpreted for the economic analysis as survival at 36 weeks of postmenstrual age without severe or moderate BPD). The result of the economic evaluation will be reported as the additional cost per additional case of death or severe or moderate BPD avoided by 36 weeks of postmenstrual age compared to conventional treatment. This analysis will only use data collected up to the assessment at 36 weeks of postmenstrual date or discharge, whichever is later.

The second within-trial analysis will be based on the clinical endpoint assessed at two years of age, of survival without severe or moderate neurodevelopmental disability. The analysis will include all cost and resource use data up to the infant reaching 2 years of age, based on data from the parent report and neurodevelopment assessment. It may be deemed appropriate to model beyond the end point of the trial if sufficient data are available. However, the limitations of modelling beyond this point will be emphasised.

A bootstrapping approach to calculate the confidence intervals around differences in costs will be used to account for the skew inherent in most cost data. As a first step the analysis will take the form of a cost-consequences analysis, reporting data in a disaggregated manner on the cost and important consequences as determined in the trial. If a situation of dominance exists where, for example, the new intervention is more costly but less effective than the current intervention (dominated by the existing intervention) or conversely less costly but more effective than the existing intervention (the new intervention dominates the existing intervention), then the cost consequence analysis would establish that no further analysis is required. However, it is more likely that any additional benefit will be accompanied by additional costs and so a full incremental economic evaluation in terms of a cost effectiveness analysis will be carried out and the results presented in terms of additional cost per additional unit of effect.

10.5. Measures to Minimise Bias

The allocation of trial treatment is randomly assigned and concealed using a central secure web-based system. Trials medications are masked such that medical and nursing staff, as well as outcome assessors and parents will be unaware of the trial medication administered. No crossover of groups is allowed; however, open-label treatment will be permitted if certain pre-defined criteria are met.

11. Source Data/Documents

Direct access to source data/documents (including hospital records/notes, clinical charts, laboratory reports, pharmacy records and test reports) will be granted to authorised representatives from the NPEU CTU, the Sponsor, the MHRA and the host organisation to permit trial-related monitoring, audits and inspections.

12. Quality Control and Assurance

12.1. Risk Assessment

The NPEU CTU has performed a risk assessment of the trial prior to commencement that will be reviewed at regular intervals during the course of the trial.

12.2. National Registration Systems

The trial will be registered on at least one global trial register.

An International Standard Randomised Controlled Trial Number (ISRCTN) has also been sought.

All babies will be registered on the HSCIC register.

12.3. Site Initiation and Training

Initiation visits at each participating neonatal unit will be performed by the Chief Investigator or his delegate and a Local Research Nurse (LRN) once all appropriate approvals are in place and IMP has been shipped to the site. Site staff will be trained on trial procedures.

The LRN will ensure adherence to the protocol and deal with any specific site issues. They will also be responsible for organising trial days to ensure that all appropriate site staff are kept fully appraised of issues such as recruitment status, informed consent, data collection, follow-up and changing regulations.

12.4. Site Monitoring and Auditing

The LRN, along with the PI, will facilitate the day-to-day smooth running of the trial at the site. They will encourage recruitment, provide staff education and training, and monitor data completeness and quality.

The LRN will submit written site visit reports to an appropriate representative of the Project Management Group (PMG) based at the NPEU CTU. No routine monitoring will be carried out unless there is cause for concern regarding the conduct of the trial at a site as a result of central monitoring. Similarly, sites will only be audited if there is a reason. This level of monitoring is justified by the level of risk associated with the trial and the use of IMP.

13. Serious Breach of Good Clinical Practice or the Trial Protocol

The MHRA require that they be informed of all serious breaches in good clinical practice (GCP) or the trial protocol within 7 days of the Sponsor becoming aware of the breach.

A serious breach is defined as a breach of GCP or the trial protocol which is likely to affect to a significant degree –

- The safety or physical or mental integrity of the patient in the trial or
- The scientific value of the trial

In the event that a serious breach is suspected the Trial Co-ordinating Centre should be contacted as soon as possible. The Trial Co-ordinating Centre will refer the serious breach onto the Sponsor immediately.
The Chief Investigator or their delegate will also notify any protocol violations to the Sponsor and will notify the REC of these in accordance with trial procedures.

14. Ethics

14.1. Declaration of Helsinki

The Investigators will ensure that this trial is conducted in accordance with the principles of the Declaration of Helsinki.

14.2. Guidelines for Good Clinical Practice

The Investigators will ensure that this trial is conducted in accordance with relevant regulations and with Good Clinical Practice.

14.3. Approvals

The trial will only start after gaining approval from the MHRA and a registered REC.

Additionally, approval of the appropriate NHS Trust Research and Development Office will be sought for individual trial sites.

Applications will be submitted through the Integrated Research Application System (IRAS).

A copy of the protocol, Parent Information Leaflet and Informed Consent Form, and GP letter will be submitted to the MHRA and the REC for approval. The Chief Investigator or their delegate will submit and, where necessary, obtain approval from the MHRA and REC for any substantial amendments. Substantial amendments are defined as those that affect:

- the safety or physical or mental integrity of the participants of the trial;
- the scientific value of the trial;
- the conduct or management of the trial; or
- the quality or safety of any investigational medicinal product used in the trial.

14.4. Participant Confidentiality, Data Handling and Record Keeping

Overall responsibility for ensuring that each participant's information is kept confidential will lie with the trial Sponsor. All paper documents will be stored securely and kept in strict confidence in compliance with the Data Protection Act (1998). Data collected on the Case Report Forms will be transferred for storage in an electronic database held by the Trial Co-ordinating Centre in which the participant will be identified only by a trial specific number.

Contact details of the baby's parent(s), as well as the baby's name (if known) and any other identifying details will be stored in a separate database also held at the NPEU CTU. This database will only be linked to the database containing trial data by the baby's trial number.

After the trial has been completed and the reports published, the data will be archived in a secure physical or electronic location with controlled access.

Electronic files will be stored on a file server that has restricted access. The server is in a secure location and access is restricted to a few named individuals. Access to the building in which the NPEU CTU is situated is via an electronic tag and individual rooms are kept locked when unoccupied. Authorisation to access restricted areas of the NPEU CTU network is as described in the NPEU CTU security policy. Data will be processed on a workstation by authorised staff. The computer workstations access the network via a login name and password which is changed regularly. No data are stored on individual workstations. Back-up of data is done automatically overnight to an offsite storage area. The location of the back-up computer is in a separate department, which has electronic tag access. Access to the room in which the back-up machine is located is via a key-pad system.

14.5. Retention of Personal Data

Personal data will be needed to contact parents when their children are 2 years of age, to co-ordinate follow up, and to disseminate the results of the trial to parent(s). Due to the nature of neonatal research the NPEU policy is to keep personal data for a period of no less than 25 years in order to follow-up on health related issues, which may become relevant in the future. At all times personal data will be held securely and will not be used for any other purpose.

14.6. Funding

The National Institute for Health Research (NIHR) Health Technology Assessment (HTA) programme is funding the trial.

14.7. Insurance

The University has a specialist insurance policy in place which would operate in the event of any participant suffering harm as a result of their involvement in the research (Newline Underwriting Management Ltd, at Lloyd's of London). NHS indemnity operates in respect of the clinical treatment which is provided.

15. Trial Governance

15.1. Site Research and Development Approval

Individual sites will only commence recruiting participants once they receive approval from NHS Trust Research and Development (R&D) Offices. Applications to R&D offices will be submitted through the NIHR Co-ordinated System for gaining NHS permission.

15.2. Trial Sponsor

The University of Oxford is the nominated Sponsor for the trial.

15.3. Co-ordinating Centre

The Trial Co-ordinating Centre will be at the NPEU CTU, University of Oxford where the Trial Co-ordinator will be based. The NPEU CTU will be responsible for all trial programming, randomisation, data entry, statistical analyses and, in collaboration with the Chief Investigator and the Local Research Nurse(s), manage the day-to-day running of the trial including recruitment of centres and training of staff. The NPEU CTU will also service both the DMC and TSC.

15.4. Project Management Group (PMG)

The trial will be supervised on a day-to-day basis by the Project Management Group. This

group reports to the Trial Steering Committee (TSC) which is responsible to the trial sponsor.

The core PMG will consist of the CI and NPEU CTU staff including:

- CTU Director
- Head of Trials Programming
- Senior Trials Manager
- Senior Trials Programmer
- Trial Co-ordinator
- Trial Statistician
- Trial Programmer
- Administrator/Data Manager

The core PMG will meet regularly (at least monthly).

15.5. Co-investigators' Group (CIG)

The CIG will meet at least twice a year. This will comprise all co-applicants and the members of the core PMG.

15.6. Trial Steering Committee (TSC)

The trial will be overseen by a TSC consisting of an independent chair and at least two other independent members. Committee members will be deemed to be independent if they are not involved in trial recruitment and are not employed by any organisation directly involved in the trial conduct.

Representatives from relevant Patient/Public Involvement groups, the Chief Investigator, other investigators/co-applicants will be joined by observers from the NPEU CTU. The HTA programme manager will be invited to attend all TSC meetings.

The role of the TSC is to provide the overall supervision of the trial. The TSC should monitor the progress of the trial and conduct and advise on its scientific credibility. The TSC will consider and act, as appropriate, upon the recommendations of the DMC and ultimately carries the responsibility for deciding whether a trial needs to be stopped on grounds of safety or efficacy.

15.7. Data Monitoring Committee (DMC)

A DMC, independent of the applicants and of the TSC, will review the progress of the trial at least annually and provide advice on the conduct of the trial to the TSC and (via the TSC) to the HTA. The committee will periodically review trial progress and outcomes as well as secondary outcomes (e.g. death, severe IVH, etc.). The content and timings of the DMC reviews will be detailed in a DMC Charter, which will be agreed at its first meeting.

16. Publication Policy/Acknowledgement of Contribution

The success of the trial depends on a large number of neonatal nurses, neonatologists, and parent(s). Credit for the trial findings will be given to all who have collaborated and participated in the trial including all local co-ordinators and collaborators, members of the trial committees, the Baby-OSCAR Co-ordinating Centre and trial staff. Authorship at the head of the primary results paper will take the form "[name], [name] and [name] on behalf of the 'The Baby-OSCAR Collaborative Group'". The drafting of the paper will be the responsibility of a writing committee. All contributors to the trial will be listed at the end of the main paper, with their contribution identified.

It is the intention of the Baby-OSCAR Collaborative Group to publish the protocol, and three peer-reviewed articles detailing, (i) the analysis of key short-term outcomes, including the incidence of death by 36 weeks of postmenstrual age, or moderate or severe BPD at 36 weeks of postmenstrual age, and other secondary outcomes up to discharge from the neonatal unit; (ii) long-term outcomes including survival without moderate or severe neurodevelopmental disability at 2 years of age corrected for prematurity, and (iii) the economic analysis.

Parents will be sent a summary of trial publications if they wish, which will contain full references.

17. References

Aranda JV, Clyman R, Cox B, Van Overmeire B, Wozniak P, Sosenko I, et al. A randomized, double-blind, placebo-controlled trial on intravenous ibuprofen L-lysine for the early closure of nonsymptomatic patent ductus arteriosus within 72 hours of birth in extremely low-birth-weight infants. *Am J Perinatol* 2009;26(3):235–45.

Bancalari E, Claure N, Gonzalez A. Patent ductus arteriosus and respiratory outcome in premature infants. *Biol Neonate* 2005;88(3):192‒201.

Bose CL, Laughon MM. Patent ductus arteriosus: lack of evidence for common treatments. *Arch Dis Child Fetal Neonatal Ed* 2007;92(6):F498‒502.

Brennen CK, Morris TP. Analysis of multicentre trials with continuous outcomes: when and how should we account for centre effects? Statistics in Medicine 2013; 32: 1136–1149.

Clyman RI. The role of patent ductus arteriosus and its treatment in the development of bronchopulmonary dysplasia. Seminars in Perinatology 2013; 37:102‒7.

Clyman RI, Cassady G, Kirklin JK, Collins M, Philips JB, 3rd. The role of patent ductus arteriosus ligation in bronchopulmonary dysplasia: re-examining a randomized controlled trial. *J Pediatr* 2009;154(6):873‒6.

Clyman RI, Chorne N. Patent ductus arteriosus: evidence for and against treatment. *J Pediatr* 2007;150(3):216‒9.

Clyman RI, Saha S, Jobe A, Oh W. Indomethacin prophylaxis for preterm infants: the impact of 2 multicentered randomized controlled trials on clinical practice. *J Pediatr* 2007;150(1):46‒50 e2.

Cooke L, Steer PA, Woodgate PG. Indomethacin for asymptomatic patent ductus arteriosus in preterm infants. *Cochrane Database of Systematic Reviews* 2003, Issue 1. Art. No.: CD003745. DOI: 10.1002/14651858.CD003745

Dereddy N. Surgical ligation for patent ductus arteriosus. *J Pediatr* 2011;158(2):343

Enders CK. *Applied Missing Data Analysis.* New York: Guilford Press, 2010.

Fowlie PW, Davis PG, McGuire W. Prophylactic intravenous indomethacin for preventing mortality and morbidity in preterm infants. *Cochrane Database of Systematic Reviews* 2010, Issue 7. Art. No.: CD000174. DOI: 10.1002/14651858.CD000174.pub2.

Hamrick SE, Hansmann G. Patent ductus arteriosus of the preterm infant. *Pediatrics* 2010;125(5):1020‒30.

Johnson S, Bountziouka V, Linsell L, Brocklehurst P, Marlow N, Wolke D, Manktelow B. Parent Report of Children's Abilities – Revised (PARCA-R). Technical and Interpretive Manual. University of Leicester, Leicester, 2019.

Jones LJ, Craven PD, Attia J, Thakkinstian A, Wright I. Network meta-analysis of indomethacin versus ibuprofen versus placebo for PDA in preterm infants. *Arch Dis Child*

Fetal Neonatal Ed 2011;96(1):F45−52.

Kluckow M, Evans N. Early echocardiographic prediction of symptomatic patent ductus arteriosus in preterm infants undergoing mechanical ventilation. *J Pediatr* 1995;127(5):774–9.

Martin AJ, Darlow BA, Salt A, Hague W, Sebastian L, McNeill N, Tarnow-Mordi W. Performance of the Parent Report of Children's Abilities-Revised (PARCA-R) versus the Bayley Scales of Infant Development III. *Arch Dis Child Fetal Neonatal Ed*. 2013; 98: 955–8.

Mangham LJ, Petrou S, Doyle LW, Draper ES, Marlow N, The cost of preterm birth throughout childhood in England and Wales. *Pediatrics*. 2009;123(2):e312–27.

Nemerofsky SL, Parravicini E, Bateman D, Kleinman C, Polin RA, Lorenz JM. The ductus arteriosus rarely requires treatment in infants > 1000 grams. *Am J Perinatol. 2008 Nov;25(10):661-6. doi: 10.1055/s-0028-1090594. Epub 2008 Oct 10.*

Neonatal Survey Database from the Trent Region 2010; http://www.le.ac.uk/departments/health-sciences/research/timms/projects/tns

Noori S, McCoy M, Friedlich P, Bright B, Gottipati V, Seri I, Sekar K. Failure of ductus arteriosus closure is associated with increased mortality in preterm infants. *Pediatrics* 2009;123; 138–e144.

Noori S. Patent ductus arteriosus in the preterm infant: to treat or not to treat? *J Perinatol* 2010;30 Suppl:S31−7.

Ohlsson A, Shah SS. Ibuprofen for the prevention of patent ductus arteriosus in preterm and/or low birth weight infants. *Cochrane Database of Systematic Reviews* 2011(1):CD004213.

Ohlsson A, Walia R, Shah SS. Ibuprofen for the treatment of patent ductus arteriosus in preterm and/or low birth weight infants. *Cochrane Database of Systematic Reviews* 2010(4):CD003481.

Oncel MY, Yurttutan S, Degirmencioglu H, Uras N, Altug N, Erdeve O, Dilmen U: Intravenous paracetamol treatment in the management of patent ductus arteriosus in extremely low birth weight infants. *Neonatology* 2013;103:166–9.

Oncel MY, Yurttutan S, Uras N, Altug N, Ozdemir R, Ekmen S, Erdeve O, Dilmen U: An alternative drug (paracetamol) in the management of patent ductus arteriosus in ibuprofen-resistant or contraindicated preterm infants. *Arch Dis Child Fetal Neonatal Ed* 2013;98:94.

Petrou S, Eddama O, Mangham L. A structured review of the recent literature on the economic consequences of preterm birth. *Arch Dis Child Fetal Neonatal Ed* 2011; 96; F225−32.

Petrou S, Mehta Z, Hockley C, Cook-Mozaffari P, Henderson J, Goldacre M. The impact of preterm birth on hospital inpatient admissions and costs during the first 5 years of life. Pediatrics. 2003; 112 (6 Pt1);1290–7.

Roberts TE, Barton PM, Auguste PE, Middleton LJ, Furmston AT, Ewer AK. Pulse oximetry

as a screening test for congenital heart defects in newborn infants: a cost-effectiveness analysis. *Arch Dis Child* 2012;97(3) :221–6.

Schmidt B, Davis P, Moddemann D, Ohlsson A, Roberts RS, Saigal S, et al. Long-term effects of indomethacin prophylaxis in extremely-low-birth-weight infants. *N Engl J Med* 2001;344(26):1966–72.

Schmidt B, Roberts RS, Fanaroff A, Davis P, Kirpalani HM, Nwaesei C, et al. Indomethacin prophylaxis, patent ductus arteriosus, and the risk of bronchopulmonary dysplasia: further analyses from the Trial of Indomethacin Prophylaxis in Preterms (TIPP). *J Pediatr* 2006;148(6):730–4.

Sellmer A, Vandborg Bjerre J, Schmidt MR, McNamara PJ, Hjortdal VE, Host B, Bech BH, Henriksen TB: Morbidity and mortality in preterm neonates with patent ductus arteriosus on day 3. *Arch Dis Child Fetal Neonatal* Published online first 26 July 2013; 10.1136/archdischild-2013-303816.

Stoll BJ, Hansen NI, Bell EF, Shankaran S, Laptook AR, Walsh MC, et al. Neonatal outcomes of extremely preterm infants from the NICHD Neonatal Research Network. *Pediatrics* 2010;126(3):443–56.

Su BH, Lin HC, Chiu HY, Hsieh HY, Chen HH, Tsai YC. Comparison of ibuprofen and indomethacin for early-targeted treatment of patent ductus arteriosus in extremely premature infants: a randomised controlled trial. *Arch Dis Child Fetal Neonatal Ed* 2008;93(2):F94–9.

Su BH, Watanabe T, Shimizu M, Yanagisawa M. Echocardiographic assessment of patent ductus arteriosus shunt flow pattern in premature infants. *Arch Dis Child Fetal Neonatal Ed* 1997;77(1):F36–40.

Sweet DG, Carnielli V, Greisen G, Hallman M, Ozek E, Plavka R, et al. European consensus guidelines on the management of neonatal respiratory distress syndrome in preterm infants - 2013 update. *Neonatology.* 2013;103(4):353–68.

Tin W, Wariyar U, Hey E. Changing prognosis for babies of less than 28 weeks' gestation in the north of England between 1983 and 1994. Northern Neonatal Network. *BMJ* 1997;314(7074):107–11.

Van Overmeire B, Allegaert K, Casaer A, Debauche C, Decaluwe W, Jespers A, et al. Prophylactic ibuprofen in premature infants: a multicentre, randomised, double-blind, placebo-controlled trial. *Lancet* 2004;364(9449):1945–9.

Zou G. A modification poisson regression approach to prospective studies with binary data. *Am J Epidemiol. 2004 Apr 1;159(7): 702–6.*

Amendment No.	Protocol Version No.	Date issued	Author(s) of changes	Details of Changes made
19	8.0	28.06.2022	Sarah Turner	Amendment made to total trial duration, following the recent trial extension granted to Baby-OSCAR

NPEU Clinical Trials Unit
National Perinatal Epidemiology Unit (NPEU)
Nuffield Department of Population Health
University of Oxford, Old Road Campus, Headington
Oxford, OX3 7LF

01865 617 965 01865 289 740
baby-oscar@npeu.ox.ac.uk
www.npeu.ox.ac.uk/baby-oscar

Baby-OSCAR is funded by the National Institute for Health Research HTA Programme (project reference 11/92/15)

Summary of all protocol amendments

Version	Date	Summary of changes
2.0	10th Dec 2014	First version approved by MHRA and REC.<br><br>Protocol Version 2, 10 Dec 2014 has been created taking into account the recent comments (28/11/14) from the MHRA to the CTA in sections 7.9, Stopping Trial Intervention and 8.1, Dosing and Administration. Further amendments have been made to provide clarity to the reader/health care professional and are summarised below:<br>• Further clarification with regards to flow velocity in PDA<br>• Structural heart disease requiring treatment has been removed and been replaced with 'Clinical or echocardiography suspicion of congenital structural heart disease that contraindicates treatment with ibuprofen'<br>• Antenatal exposure to cyclo-oxygenase (COX) inhibitors has been removed since we could be excluding a number of babies unnecessarily<br>• Further clarification with regards to the recording of Adverse Events, from first dose until 7 days after of trial medication and only Unforeseeable SAEs will be recorded<br>• Clarification on the secondary endpoints have been made in particular; Death at 36 weeks' postmenstrual age; Moderate or severe BPD at 36 weeks postmenstrual age; Severity of BPD at 36 weeks postmenstrual age; Closed or non-significant PDA (<1.5mm) at 3 weeks of age, confirmed by ECHO; PDA =1.5mm at 3 weeks, not treated medically or by surgical closure; Medical rescue treatment of a symptomatic PDA with a COX inhibitor; Medical rescue treatment of a symptomatic PDA by a surgical treatment.<br>• Total duration of respiratory support has also been broken into invasive ventilation and non-invasive support<br>• Survival has been added to 'Long term outcomes'<br>• Process outcomes have also been added to incorporate:<br>- Number of doses received<br>- Adherence to protocol<br>- Study withdrawals<br>• Foreseeable Serious Adverse Events have been reviewed, in addition a section on 'Causality' of the IMP and adverse event has been written.<br>• Reporting Procedures have also been reviewed to make them more explicit.<br><br>The first version of the protocol draft stated the primary outcome as death or severe neuro-developmental disability at 2 years corrected age assessed using Bayley's scale of infant development III. This was amended after discussion and before the NIHR-HTA funding application submission and registries were updated. The primary outcome in version 2 of the protocol was amended to a composite outcome of death or bronchopulmonary dysplasia (BPD) at 36 weeks' post-menstrual age using an

		oxygen reduction test. Version 2 of the protocol was the first Research Ethics Committee (REC) and Medicines and Healthcare products Regulatory Agency (MHRA) approved protocol for use in the trial. This primary outcome remained constant in subsequent approved versions of protocol, but was clarified explicitly by describing it as a composite outcome of death by 36 weeks' post-menstrual age or moderate or severe BPD at 36 weeks' post-menstrual age, defined using severity-based diagnostic criteria for BPD. (Reference: Jobe AH, Bancalari E. Bronchopulmonary dysplasia. Am J Respir Crit Care Med. 2001 Jun;163(7):1723-9. doi: 10.1164/ajrccm.163.7.2011060. PMID: 11401896).
3.0	20 April 2015	Amendments to the duration of the infusion (pages 9 and 27) have been in accordance with the Reference Safety Information for NeoProfen.
4.0	8 June 2015	The number of trial sites was increased from 4 to 5 (pages 6, 17); bell stage and IVH grades were converted from numerical to roman numerals (as per standard conventions) - pages 6, 7, 16, 19, 38 page 48 - typing amendment (funding to funded).
5.0	4 Oct 2016	Changes to dosages, reflecting use of Pedea® (dosing and safety) and further changes necessitated by the change in the placebo manufacturer.
6.0	17 Nov 2016	In summary, the amendments are as follows:<br>The front page:<br>Telephone number and IRAS No. has been incorporated; the signatures for the Chief Investigator and statistician have been removed - they will be documented separately and filed with the protocol in the Trial Master File.<br><br>Inclusion criteria:<br>Updated under 'Echocardiography'; The Pulsatile flow is clarified by using the flow ratio of > 2:1. This will objectively differentiate a closing pattern (ratio < 2:1 - not eligible for trial inclusion) from pulsatile pattern (ratio > 2:1 – eligible for inclusion in the trial) on echo screening. The growing pattern (< 30% right to left) is still considered transitional change similar to Pulsatile flow for haemodynamic significance of PDA. Any right to left flow > 30% is an exclusion criterion because such babies are monitored for development of pulmonary hypertension (in addition to other clinical and investigational criteria).<br><br>Exclusion criteria under 'Other conditions':<br>Haemorrhages have been further clarified.<br><br>Some grammatical amendments throughout the protocol, primarily to the primary objective/endpoints and secondary objectives e.g. weeks of postmenstrual age, rather than weeks' PMA.<br><br>Secondary Objectives:

		This is a *NEW* update where we have condensed the secondary objectives, as, i) this reduces unnecessary repetition, and ii) the outcomes are fully detailed under the 'Secondary Endpoints' section.<br><br>Secondary Endpoints:<br>Short term outcomes have been updated and expanded, including 'non-cystic PVL' - these are self-explanatory standard neonatal terms and follow a standard approach for clinical or ultra-sonographic diagnosis, as detailed.<br>They are routine assessments on premature babies requiring intensive care and hence further explanation is not provided. Assessment of growth is vital and the anthropometric assessments reflect growth. Growth measurements encompass the measurement of height, weight and head circumference. An abnormal rate of growth could suggest an underlying problem that could be a complication of prematurity or be multifactorial. This is a standard assessment tool to reflect the response to clinical interventions. An increasing head circumference for example could be the first or only indicator of a problem such as hydrocephalus. The long term outcomes at 2 years of age reflect the benefits of intervention sustained after discharge and are of clinical and public health interest. We, in fact, plan to follow these babies even longer at 5 years' age depending on securing separate funding.<br><br>A cost-effectiveness analysis will be conducted of deaths and BPD events avoided and national health services used up to 2 years of age corrected for prematurity was removed in error and has been reinstated.<br><br>2 Trial Flow Diagram:<br>Updated to 'weeks of postmenstrual age.<br><br>Abbreviations in section 3:<br>Updated.<br><br>Sections 5.1, 5.2, 6.2 and 6.5 – updated as per previous comments.<br><br>6.5.1<br>*NEW*<br>Flow diagram has been updated to give clearer guidance.<br><br>Section 6.6 has been updated, as detailed before.<br><br>7.1 Trial Assessments table:<br>This has been reworked.<br>Clarity as to when echocardiographic assessment can be conducted has also been given.

		7.4 Echocardiograms:<br>Range given in days as well as weeks.<br><br>7.5 Informed Consent:<br>In the previous version, changes to the informed consent process were made as the pilot study showed that consent is possible prior to the echocardiograhpic assessment. We included the option of telephone consent for those rare instances where a baby was transferred to a recruiting site from a hospital not participating in Baby-OSCAR and the mother, due to various reasons, was not transferred with the baby.<br>During the pilot study there were a few babies who could have been eligible for the trial as the recruiting site staff had a good telephonic relationship with the parents, but because no procedures were in place, recruitment was not feasible.<br>The REC were not happy with this as it was felt that parents would not have enough time to consider the information, and it was suggested to rework it. Due to time constraints the Project Management Group felt that it was best to revisit this procedure with the REC at a later date.<br><br>Sections 7.6, 7.7, 7.8, 7.9 and 7.11 have minor grammatical updates.<br><br>8.3 Accountability:<br>This has been reworked.<br><br>8.4 Open-label Treatment:<br>*NEW*<br>Grammatical changes to the text (colons versus commas).<br><br>9.1.4 Foreseeable Serious Adverse Events:<br>For ease, the conditions have been sorted alphabetically and this was hard to demonstrate tracked, so we have crossed the section out and inserted the conditions by alpha order.<br>There were some changes, namely to:<br>- Fluid retention;<br>- Gastrointestinal bleeding (not haemorrhage);<br>- High blood creatinine has been added;<br>- hypotension ‘treated with inotropes’ added;<br>- Low serum level is new;<br>- Defined neutropenia;<br>- ROP removed – as these are secondary outcome measures;<br>- Sepsis has been removed;<br>- Seizures reworked;

		- Pulmonary haemorrhage was repeated, so one has been removed the word 'significant' included.<br>- Thrombocytopenia has been added, in line with the SmPC for Pedea®.<br><br>Hypoglycemia and hyperglycemia are commonly encountered in preterm babies born below 29 weeks' gestation receiving neonatal intensive care. As per the product characteristics, the risk of hypo or hyperglycemia does not increase with the use of Pedea® (IMP in Baby-OSCAR trial). Hence, we will include hypoglycemia and hyperglycemia as an expected SAE in the trial participants, but will not report data on its occurrence in the trial study groups while babies receive standard neonatal intensive care.<br>Given that the babies in the trial are extremely pre-term, and the expectation is that they will be in various stages of respiratory failure, the occurrence of respiratory failure will not be reported in relation to trial medication.<br><br>10.1 Sample Size:<br>Minor grammatical amendments.<br><br>10.2 Assessment of Outcomes:<br>Minor grammatical amendments.<br>We have also been informed that HSCIC is changing their name and have included 'or a named derivative', should their name change again during the course of the trial.<br><br>10.3.5 Dealing with missing data:<br>More grammatical changes.<br><br>Sections 10.5, 12.5, 13, 14.3, 14.4 and 16: Grammar updates.<br><br>12.5 Blinded Endpoint Review:<br>Updated in line with other outcomes.<br><br>Section 17 Protocol Signatures:<br>This page has been removed as the protocol will be appended to the site agreement, circulated separately and the principal investigator must sign the agreement and appended documents before the trial can start at their site.<br>The site agreements are renewed every 12 months. Should there be any further protocol amendments between this period, an updated protocol will be circulated, along with a separate sign off page for the PI, which will be collected and shall remain in the Trial Master File.

7.0	10 Aug 2020	Changes to Protocol:<br>• Statistical methods for the short-term outcomes were reviewed and updated in line with the SAP (section 10.3.2 Statistical Methods)<br>• The long-term PARCA-R outcome was updated to the standardised scores recommended by the recently published manual and a sensitivity analysis was added<br>• An additional long term outcome was added – duration of oxygen supplementation since randomisation<br>• Added subgroup analysis of NEC Bell stage II and above by size of the PDA<br>• All pre-specified outcome comparisons will be presented with 95% confidence intervals, changed from the 99% confidence intervals for secondary outcomes. There will be a pre-specified list of tested and untested outcomes included in the Statistical Analysis Plan (section 10.3.4 Level of Statistical Significance)<br>• the Blinded Endpoint Review (section 12.5) has been removed<br>• included new NPEU CTU logo<br>• updated references (section 17)
8.0	4 July 2022	Amend to the length of trial to reflect the extension granted to BABY OSCAR 2022

Outcome after Selective Early Treatment for Closure of Patent Ductus ARteriosus in Preterm Babies

Statistical Analysis Plan for Long Term Outcomes

Version 1.0

Date: 20 April 2023

Author: *Charlotte Clarke (Statistical Fellow, NDPH)*
Reviewers: *Pollyanna Hardy (Director, NPEU CTU)*
Heather O'Connor (Senior statistician, NPEU CTU)
Prof Samir Gupta (Chief Investigator)
Prof Samantha Johnson (Co-investigator)

Contents

1. Introduction 5
2. Background 6
3. Interventions 6
   3.1 Intervention (ibuprofen) group 6
   3.2 Control (placebo) group 6
4. Description of two-year outcomes 6
   4.1 Main long-term outcome 6
   4.2 Other long-term outcomes 7
      4.2.1 Individual components of the main long-term outcome 7
      4.2.2 Respiratory morbidity 7
5. Sample size and power 7
6. Random allocation 8
7. Protocol and data non-compliances 8
   7.1 Major protocol non-compliers 8
   7.2 Minor protocol non-compliers 8
   7.3 Data non-compliers 9
8. Interim analyses 9
9. Data collection 10
10. Derivation of variables 10
   10.1 Corrected age 10
   10.2 Survival at 24 months corrected age 10
   10.3 Infant neurodevelopment 10
   10.4 Survival without moderate or severe neurodevelopmental impairment 11
   10.5 Respiratory morbidity 12
   10.6 Survival without respiratory morbidity 12
   10.7 Duration of oxygen supplementation from randomisation 12
11. Participant groups for analysis 13
   11.1 Primary analysis strategies 13
   11.2 Post-randomisation exclusions 13
   11.3 Descriptive analysis population 13
   11.4 Comparative analysis population 13
   11.5 Interim analysis population 13
12. Descriptive analyses 13
   12.1 Representativeness of trial population and participant throughput 13

12.2 Baseline comparability of randomised groups 14
12.4 Loss to follow-up 15
13. Comparative analyses 15
13.1 Main long-term outcome 16
13.2 Other long-term outcomes 16
14. Secondary analysis 16
15. Pre-specified subgroup analysis 17
16. Sensitivity analysis 17
17. Safety data analysis 17
18. Statistical significance 17
19. Procedure for accounting for missing data 17
20. Deviation from the protocol 18
21. Statistical software employed 18
22. Additional exploratory analysis 18
23. Dummy tables 18
24. Health economic analysis 18
25. References 19
26. Additional documents 19
27. Approval 19

List of abbreviations:

BERC	Blinded End-point Review Committee
BPD	Bronchopulmonary dysplasia
CI	Confidence interval
CONSORT	Consolidated standards of reporting trials
COX	Cyclo-oxygenase
CPAP	Continuous Positive Airway Pressure
CRIB II	Clinical risk index for babies score II
CRF	Case report form
CTU	Clinical trials unit
DMC	Data monitoring committee
ECHO	Echocardiography
GP	General practitioner
HTA	Health technology assessment
IMP	Investigational Medicinal Product
IVH	Intraventricular haemorrhage
ITT	Intention to treat
Kg	Kilograms
L	Litre
mg	Milligram
min	Minute
ml	Millilitre
mm	Millimetre
NEC	Necrotising enterocolitis
NIHR	National Institute for Health Research
NNU	Neonatal unit
NPEU	National Perinatal Epidemiology Unit
PARCA-R	Parent Report of Children's Abilities – Revised
PDA	Patent ductus arteriosus
PVL	Periventricular leukomalacia
RCT	Randomised controlled trial
ROP	Retinopathy of prematurity
UK	United Kingdom

1. Introduction

This document details the proposed presentation and analyses for the long-term outcomes of the Baby-OSCAR trial, a multi-centre, masked, randomised controlled trial (RCT) funded by the National Institute for Health Research (NIHR) Health Technology Assessment (HTA) programme. The analysis strategy for short-term outcomes at hospital discharge are documented in a separate analysis plan.

The analysis plan will be available upon request when the principal manuscripts are submitted for publication. Suggestions for subsequent analyses by journal editors or referees will be considered carefully and carried out, as far as possible, in line with the principles of this analysis plan. If reported, the source of the suggestion will be acknowledged. The health economic outcomes and analysis will be outlined in a separate analysis plan.

Any deviations from the statistical analysis plan will be described along with the rationale given in the final report of the trial. The analysis will be carried out by an identified, appropriately qualified and experienced statistician, who will ensure the integrity of the data during processing. This document and the final analysis will be produced in line with NPEU Standard Operating Procedures ST105 Statistical Analysis Plan and ST107 Statistical Analysis and Reporting.

2. Background

The Baby-OSCAR trial is a masked, multi-centre, randomised, placebo-controlled, parallel group trial to determine short- and long-term health and economic outcomes of the treatment of a large Patent Ductus Arteriosus (PDA) in extremely preterm babies with ibuprofen within 72 hours of birth.

The trial followed an internal pilot phase, which was run to assess the suitability of trial procedures and likelihood of recruitment targets being achieved. A total of 653 out of a target of 730 infants were recruited (including those recruited during the internal pilot phase) between 12 July 2015 and 31 Dec 2020 from 35 UK tertiary neonatal units (NNUs).

3. Interventions

3.1 Intervention (ibuprofen) group

The intervention was given as an initial loading dose of 10 mg/kg (2 ml/kg) of ibuprofen, followed by two 5 mg/ml (1 ml/kg) doses at 24 and 48 hours after the initial dose. Doses were calculated on the birth weight of the baby. If required, the investigational medicinal product (IMP) was diluted to appropriate volume with 5% glucose or 0.9% Sodium Chloride. Each dose was given as a short dose intravenous infusion over 15 minutes. All 3 doses were given unless there were adverse effects necessitating stoppage, as referenced in the trial protocol, section 7.9.

3.2 Control (placebo) group

The placebo was given as a clear sterile solution of 0.9% Sodium Chloride for injection. Cartons identical to those for ibuprofen, each containing four identical single use ampoules, were provided. The volume to be withdrawn from the ampoule was calculated following the same methods for ibuprofen dosing.

4. Description of two-year outcomes

All outcomes at 24 months of age corrected for prematurity.

4.1 Main long-term outcome

Survival without moderate or severe neurodevelopmental impairment (defined as any one or more of moderate or severe non-verbal cognitive, language, gross motor, hearing or visual impairment, each of which will additionally be presented descriptively only):

- Moderate or severe cognitive impairment; PARCA-R non-verbal cognitive scale score <70, or moderate or severe impairment classified by the Blinded End-point Review Committee (BERC).
- Moderate or severe language impairment; PARCA-R language scale score <70, or moderate or severe impairment classified by the BERC.
- Moderate or severe gross motor impairment; unable to walk and/ or sit independently, as reported by parents or classified by the BERC.

- Moderate or severe hearing impairment; hearing loss corrected with aids, or some hearing loss, not corrected by aids, or deaf, as reported by parents or classified by the BERC.
- Moderate or severe visual impairment; reduced vision, uncorrected with aids, or blind in one eye, or blind/ can perceive light only, as reported by parents or classified by the BERC.

4.2 Other long-term outcomes

4.2.1 Individual components of the main long-term outcome

- Death (up to 24 months of age corrected for prematurity).
- Moderate or severe neurodevelopmental impairment (defined as above), in survivors.

4.2.2 Respiratory morbidity

- Survival without respiratory morbidity (defined as any 2 or more of need for oxygen or respiratory support; presence of a persistent cough and/ or wheeze; need for regular treatment for respiratory illness; 4 or more unscheduled attendances at hospital/ GP for respiratory problems; 1 or more readmission to hospital for respiratory problems).
- Respiratory morbidity (defined as above), in survivors.
- Individual components of respiratory morbidity, in survivors, as follows (presented descriptively only):
    - Need for oxygen or respiratory support.
    - Presence of a persistent cough and/ or wheeze.
    - Need for regular treatment for respiratory illness.
    - 4 or more unscheduled attendances at hospital/ GP for respiratory problems.
    - 1 or more readmission to hospital for respiratory problems.
- Duration of oxygen supplementation from randomisation.

5. Sample size and power

The sample size for the Baby-OSCAR trial was based on clinical evidence suggesting the risk of death or bronchopulmonary dysplasia (BPD) in babies with a large PDA to be approximately 60%, and for those receiving the intervention risk of death or BPD to be approximately 48%. Assuming 1% were lost to follow-up, a sample size of 730 babies was required to detect this 12% reduction in absolute risk with 90% power and a 2-sided significance level of 5%.

The proportion of infants surviving to 24 months corrected age without moderate or severe neurodevelopmental impairment in the control group is expected to be 55%. If outcome data are available on a total sample size of 600 (including deaths), the trial will have an 80% power to detect an increase in survival without moderate or severe neurodevelopmental

impairment of 11% from 55% to 66%, and a 90% power to detect an increase of 13% from 55% to 68%.

A total number of 653 infants were recruited, of which 646 were included in the short-term outcomes analysis.

6. Random allocation

Randomisation was managed via a secure web-based randomisation facility hosted by the NPEU CTU with telephone back-up available at all times. The allocation ratio of intervention (ibuprofen) to control (placebo) was 1:1. A minimisation algorithm was used to ensure balance between the two groups with respect to the size of the PDA, gestational age at birth, age at randomisation, sex, trial site, multiple births, mode of respiratory support at randomisation; (1) invasive ventilation (by an endotracheal tube); or (2) non-invasive respiratory support through, nasal CPAP, nasal ventilation, humidified high flow nasal cannula therapy or, low flow oxygen ≥ 1.1 L/min; or (3) receiving no mechanical ventilation, or pressure support (in room air, or low flow oxygen < 1.1 L/min or ambient oxygen) and receiving inotropes or not at the time of randomisation. Babies of multiple births were randomised individually.

7. Protocol and data non-compliances

All protocol non-compliances were listed in the final report of short-term outcomes, and there are no further changes anticipated since this analysis. Protocol non-compliances are defined as below.

7.1 Major protocol non-compliers

Data considered to be fraudulent is defined as a major protocol non-compliance.

7.2 Minor protocol non-compliers

The following were defined as minor protocol non-compliances:

Participants randomised in error

These include infants:

- Who are < 23 weeks or ≥ 29 weeks of gestation.
- Who are ≥ 72 hours old.
- With a PDA < 1.5 mm in diameter OR who does not have unrestrictive pulsatile left to right flow or, growing pattern with right to left flow of 30% or more.
- Who have clinical or echocardiography evidence of pulmonary hypertension.
- Where written informed consent has not been obtained from the parent(s).
- With a severe congenital anomaly.
- With contraindications to the use of ibuprofen.
- Who have had indomethacin, ibuprofen, or paracetamol administered after birth.

Treatment non-compliances

These include infants who:

- Do not receive allocated intervention. These include infants who were allocated ibuprofen, who instead received placebo, and vice versa.
- Do not receive the correct number of doses. These include infants who received less than 3 doses of the trial medication.
- Do not receive medication at the correct time. These include infants who received their first dose later than 72 hours after birth, or received their 2nd or 3rd dose outside the specified dosing window (< 18 hours or > 72 hours between doses 1 and 2, or doses 2 and 3, or dose 3 completed > 7 days after first dose administered).
- Received open-label treatment without meeting the criteria. These include infants who received open-label treatment but did not meet the defined criteria for doing so:
    1. Inability to wean on ventilator (ventilated for at least 7 days continuously) and any of: inability to wean oxygen; persistent hypotension; pulmonary haemorrhage; signs of cardiac failure,
       AND
    2. Echocardiographic findings of a large PDA (PDA ≥ 2.0 mm with pulsatile flow)
       AND
    3. Echocardiographic findings of hyper-dynamic circulation or ductal steal.

Trial procedure non-compliances

- ECHO not done around 3 weeks (18-24 days) of age.
- Oxygen reduction test will be reported in a process outcomes table.

7.3 Data non-compliers

The following were defined as data non-compliers:

Non-verbal cognitive and language sections of the two-year follow-up parent report questionnaire completed outside of the time window for deriving PARCA-R standard scores

These include infants:

- Who were aged less than 23.5 months or more than 27.5 months corrected age when the 24 month follow-up questionnaire was completed.

8. Interim analyses

An independent Data Monitoring Committee (DMC) was established, whose remit was to review the progress and conduct of the trial. The DMC are independent of the trial organisers and the terms of reference are documented in the DMC charter. The DMC does not plan to review any interim analyses of the two-year follow-up data.

9. **Data collection**

Data for the two-year outcomes were collected by questionnaire using the following CRF(s) sent to parents when the child was 24 months corrected age:

- Baby-OSCAR 2 Year Form – V2.0 July 2018; your child's health and development at 2 years.

This included the PARCA-R to assess cognitive and language impairment, and validated parent report items to assess gross motor, vision, and hearing impairment.

10. **Derivation of variables**

10.1 Corrected age
The expected date of delivery for each child will be used to calculate their age at 24 months corrected for prematurity:

- (Date 2-year follow-up parent report questionnaire was completed minus expected date of delivery)/ (365 divided by 12)

10.2 Survival at 24 months corrected age
Deaths before hospital discharge are recorded in CRF Form 6: Baby Outcomes. Section D of this form will determine whether the infant died before discharge. Deaths after discharge and up to 24 months of age are recorded on the Baby-OSCAR administrative database. All deaths after discharge were checked directly with the recruiting hospital.

10.3 Infant neurodevelopment
Non-verbal cognitive and language development

The PARCA-R is a parent completed questionnaire that is used to assess children's non-verbal cognitive and language development at 23.5 to 27.5 months of age. It is comprised of a non-verbal cognitive scale and a language scale. The first subscale comprises 34 items to assess non-verbal cognition. For each item, the parent is asked to respond 'yes', 'no', or 'don't know' to whether their child has exhibited a specific ability. The number of 'yes' responses are summed to produce a non-verbal cognition subscale raw score with a range from 0 to 34. The language subscale score comprises a 100-word vocabulary checklist, from which the number of words the child can say is summed to produce a score with a range from 0 to 100, along with 18 forced-choice items to obtain information regarding the child's use of sentences to produce a score with a range of 0 to 24. These are summed together to produce the language subscale raw score with a range of 0 to 124 [1]. On both the non-verbal cognitive scale and the language scale, raw scores are converted to age-standardised scores with a normative mean of 100 (SD 15).

Children with non-verbal and language assessments completed outside of the corrected age range of 23.5 to 27.5 months with no classification from the BERC will be treated as missing data and a multiple imputation analysis performed to estimate these infants' PARCA-R standardised scores (see section 13.1). Scores for missing questions on the non-verbal

cognitive scale will be substituted with the average of the score of each individual child for completed questions if 4 or fewer questions are missing. If more than 4 questions are missing, a non-verbal cognition standardised score cannot be derived. These will therefore be treated as missing data and a multiple imputation analysis will be performed to estimate the PARCA-R standardised scores of infants with 4 or more missing non-verbal cognitive questions. Reasons for missing data will be reported and described by type, based on whether the assessments were completed outside of the corrected age range, or whether there were 4 or more data points missing in the non-verbal cognitive subscale.

PARCA-R standardised scores will then be used to classify non-verbal cognitive and language impairment for which two binary outcomes (non-verbal and language) will be derived. On each scale, children with scores < -2 SD (standard score <70) will be classified with moderate or severe impairment.

Gross motor, vision and hearing impairment

Gross motor, vision and hearing impairment were assessed using parent report items with impairment classified into binary outcomes as follows:

- Moderate or severe gross motor impairment: the child is unable to sit on the floor independently (Q4 = can sit with support or with help from an adult, or unable to sit), and/ or the child is unable to walk independently (Q5 = can only walk if helped by an adult or a walking aid, or unable to walk, even with help).
- Moderate or severe hearing impairment: the child has some hearing loss corrected with aids (Q3 = has a cochlear implant or hearing aid, but hears well with it), or the child has some hearing loss, not corrected by aids (Q3 = has difficulty hearing, even with a cochlear implant or hearing aid), or the child is deaf (Q3 = my child is deaf).
- Moderate or severe visual impairment: the child has reduced vision, uncorrected with aids (Q2 = has difficulty seeing, even when wearing glasses), or the child is blind in one eye (Q2 = is blind in one eye, but has good vision in the other eye), or the child is blind or can perceive light only (Q2 = is able to see light only or is blind).

Blinded Endpoint Review Committee

The BERC reviewed and classified impairment using clinical data relating to the child's two year neurodevelopmental assessment where available for children who survived to 24 months corrected age for whom: a 2-year study questionnaire was not completed by a parent or carer; a 2-year study questionnaire was completed outside of the timeframe of 23.5 to 27.5 months corrected age; or, there are missing data on questionnaire items precluding classification of one of more of the individual components of the main 2-year outcome. The purpose of the BERC is to make a final determination on whether these children have moderate or severe neurodevelopmental impairment (for each domain and overall). For further details see the Baby-OSCAR Follow-up BERC Charter (see section 26).

10.4 Survival without moderate or severe neurodevelopmental impairment

The main long term outcome of survival without moderate or severe neurodevelopmental impairment is a binary outcome defined as surviving to 24 months corrected age without

moderate or severe neurodevelopmental impairment in non-verbal cognitive, language, gross motor, hearing or visual function.

10.5 Respiratory morbidity

Respiratory morbidity will be assessed by the need for oxygen or respiratory support which, using section A: Your Child's Health and Development of the Baby-OSCAR 2 Year Form, will be defined as having two or more of the following:

- Need for oxygen or respiratory support; the child was discharged home on oxygen using a nasal cannula (Q6=Yes), or the child received oxygen using a nasal cannula at any other time since discharge (Q6ii=Yes) or the child has received any other breathing support since being discharged home after birth (Q7=Yes).
- Presence of a persistent cough and/ or wheeze; the child has suffered from a persistent wheeze (Q8=Yes), or persistent cough (Q9=Yes and Q9i=cough affects at least one of feeding, sleeping, or physical activity), since being discharged home after birth.
- Need for regular treatment for respiratory illness; the child has required at least one of the following regular treatments since being discharged home after birth (Q10=Yes, and at least one treatment ticked): inhaler – relievers, e.g. Ventolin or Bricanyl (blue); inhaler – preventers, e.g. Pulmicort (brown), or Flixotide (yellow); steroids, e.g. Prednisolone; other (to be specified).
- Unscheduled attendances at hospital/ GP for respiratory problems; the child has been taken to a GP or Accident and Emergency department for any respiratory illness more than 3 times since being discharged home after birth (Q11=Yes and Q11i=4-12 times, or more than 12 times).
- Readmission to hospital for respiratory problems; the child has been admitted to hospital for any respiratory illness since being discharged home after birth at least once (Q12=Yes).

10.6 Survival without respiratory morbidity

Survival without respiratory morbidity is therefore a binary outcome defined as surviving to 24 months corrected age with fewer than two of the following: a need for oxygen or respiratory support, presence of a persistent cough and/ or wheeze, need for regular treatment for respiratory illness, unscheduled attendances at the hospital/ GP for respiratory problems, or readmission to hospital for respiratory problems (see section 10.5).

10.7 Duration of oxygen supplementation from randomisation

Duration of oxygen supplementation from randomisation will be determined by the sum of the total number of days up to 24 months corrected age the child was receiving invasive ventilation by ET tube, the number of days the child was receiving non-invasive respiratory support by nasal ventilation, CPAP, or high flow oxygen therapy, and the number of days the child was receiving ambient or low-flow oxygen. If the child was still receiving oxygen supplementation at 24 months corrected age this will be calculated as the number of days between their date of birth and the date the 2-year parent report questionnaire was completed.

11. **Participant groups for analysis**

11.1 Primary analysis strategies

For the two-year outcomes, the primary inference will be based on an intention-to-treat (ITT) analysis, i.e. infants will be analysed in the groups to which they were randomised, regardless of treatment they received.

11.2 Post-randomisation exclusions

Exclusions to the analysis population post-randomisation consist of the following:

- Infants for whom a written consent form from the parent(s) was not received
- Infants for whom consent to use their data was withdrawn by the parent(s)
- Infants for whom an entire record of fraudulent data was detected (should fraudulent data be detected, consideration will be given to excluding all data for the site where such data were found)

11.3 Descriptive analysis population

Baseline demographic and clinical characteristics will be reported for all infants and their mothers in the ITT population by randomised group for whom main long-term outcome data are available (including deaths).

11.4 Comparative analysis population

All infants randomised with available outcome data at two years, minus post-randomisation exclusions (see section 11.2).

11.5 Interim analysis population

The DMC does not plan to review any interim analyses of the two-year follow-up data.

12. **Descriptive analyses**

12.1 Representativeness of trial population and participant throughput

The flow of participants through each stage of the trial will be summarised by randomised group using a CONSORT diagram. This will described the following numbers of infants:

- Assessed for eligibility
- Eligible
- Randomised
- Allocated to ibuprofen
- Allocated to placebo
- Withdrawn consent
- Included in analysis of short-term primary outcome
- Deaths
- Lost to follow-up following discharge home
    - Non-response at two years with no classification by the BERC

  - o Response at two years but with too much missing data and no classification by the BERC
- Included in analysis population for the main long-term outcome

12.2 Baseline comparability of randomised groups
The following demographic and clinical characteristics collected at baseline and two years, and important short-term outcomes, will be described by randomised group for infants who had available data on the main long-outcome data at two years.

Mother's baseline characteristics:

- Ethnicity
- Age (years)
- Deprivation index
- Antenatal steroid use (any)
  - o < 24 hours before birth
  - o ≥ 24 hours before birth
- Antenatal COX inhibitor use
- Antenatal magnesium sulphate use for neuroprotection

Infant's characteristics at trial entry:

- Enrolling centre
- Born in enrolling centre
- Postnatal age at randomisation (hours)
- Gestational age at birth (weeks)
- Mode of delivery
- Forceps or Ventouse used in delivery
- Main cause of preterm birth
- Birth weight (g)
- Birth weight z score
- Head circumference (cm)
- Head circumference z score
- Sex
- Baby is one of a multiple pregnancy
- Sibling enrolled in the study (in multiple pregnancies)
- APGAR score 5 minutes after birth
- Baby's worst base excess at first hour after birth
- CRIB II (without temperature)
- Size of PDA
- Mode of respiratory support at randomisation
- Receiving inotropes at randomisation

Data collected at two years:

- Family history of asthma or wheezing

- Environmental factors

Short-term outcomes:

- **Primary outcome:** composite outcome of death by 36 weeks' postmenstrual age, or moderate or severe BPD at 36 weeks' postmenstrual age.
- Moderate or severe BPD at 36 weeks' postmenstrual age.
- Severe intraventricular haemorrhage (IVH) (grade III/ IV with ventricular dilation or intraparenchymal abnormality).
- Cystic periventricular leukomalacia (PVL).
- Babies treated for retinopathy of prematurity (ROP).
- Necrotising enterocolitis (NEC) definitive and/ or complicated (Bell stage II and above) confirmed by radiology and/ or histopathology.
- PDA ≥ 1.5 mm at around 3 weeks (range of 18-24 days), not treated medically or by surgical closure.
- Discharge home on oxygen.
- Postnatal steroid use for chronic lung disease.

The number and percentage will be presented for binary and categorical variables. The mean and standard deviation or the median and interquartile range - where data are skewed - will be presented for continuous variables, or the range if appropriate.

12.4 Loss to follow-up

The number and percentage of infants for whom there is no two-year outcome data will be reported for the two trial arms, and the reasons will be recorded. All deaths will be reported separately. Demographic and clinical characteristics at baseline and collected at two years, and important short-term outcomes, will also be described for infants for whom main long-term outcome data were and were not obtained at two years, within each trial arm.

13. **Comparative analyses**

All models will be adjusted for minimisation factors where possible (see section 6). Size of PDA, gestational age at birth, and age at randomisation will be treated as continuous variables. Sex, multiple births, and whether the infant received inotropes at the time of randomisation will be treated as binary variables. Trial site and mode of respiratory support at randomisation will be treated as categorical variables. Centre and multiple births will be treated as random effects and all other factors will be treated as fixed effects. Correlations between siblings from multiple births will be accounted for in the adjusted model by nesting "multiple" cluster as a random effect within centre. If a quantile regression is necessary, all factors will be treated as fixed effects, as random effects cannot be modelled using these methods of analysis.

Both crude and adjusted effect estimates will be presented, but the primary inference will be based on the adjusted estimates.

13.1 Main long-term outcome
A mixed-effect log binomial regression model will be fit to estimate the risk ratio (RR) and 95% confidence interval (CI) and p-value for survival without moderate or severe neurodevelopmental impairment at two-years corrected age between the control group and the intervention group. Children with PARCA-R questionnaires completed outside of the 23.5 to 27.5 months of age who did not have cognitive and language impairment classified by the BERC will be regarded as having missing standardised PARCA-R scores and will be treated as missing at random.

A multiple imputation analysis will be performed for the non-verbal cognitive and language PARCA-R standardised scores for children with PARCA-R questionnaires completed outside of the 23.5 to 27.5 months corrected age range or for those with 4 or more missing non-verbal cognitive scale questions, and without classification of cognitive and language impairment by the BERC. The continuous value of the standardised PARCA-R score will be imputed and this will then be converted to the binary outcome. The multiple imputation model will include raw PARCA-R score, corrected age, sex and any other baseline characteristics and short-term outcomes associated with the missing status of the PARCA-R score. If there is evidence that the variables used to inform the imputation are not strongly associated with the primary outcome, or if there are non-significant concerns around the specification of the imputation model, Predictive Mean Matching will be used, with the 10 closest observations (donors) informing the imputation. The estimates from this multiple imputation analysis will be presented as the primary inference [2, 3].

If the model fails to converge, a Poisson regression model with a robust variance estimator will be used. If the Poisson model fails to converge, centre will be removed as a random effect.

13.2 Other long-term outcomes
For continuous outcomes, a mixed-effect linear regression model will be fitted to estimate the mean difference and 95% CIs for the outcome variables between the control group and intervention group, assuming model assumptions are satisfied (i.e. independence and normality of residuals). If model assumptions are not satisfied, a quantile regression model will be employed, with median differences and 95% CIs presented.

For binary outcomes, log-binomial regression models will be used to calculate risk ratios and 95% CIs. Details of procedures in place for the log-binomial regression failing to converge are outlined in section 13.1.

Analysis of the main long-term outcome will be clearly delineated from the other long-term outcomes in any statistical reports produced.

14. **Secondary analysis**

There are no planned secondary analyses.

15. **Pre-specified subgroup analysis**

Pre-specified subgroup analyses will use the statistical test of interaction and where appropriate, results will be presented as risk ratios with 95% CIs.

Pre-specified subgroups on the main long-term outcome will be based on:

- Gestational age at birth (< 26 weeks; ≥ 26 weeks).
- Size of the PDA (1.5 mm to < 2.0 mm; 2.0 mm to < 3.0 mm; ≥ 3.0 mm).
- Mode of respiratory support at randomisation invasive ventilation (defined as ventilation via an endotracheal tube); or, non-invasive or no respiratory support (defined as ventilation via nasal CPAP, nasal ventilation, humidified high flow nasal cannula therapy, or low flow oxygen ≥ 1.1 L/min, or receiving no mechanical ventilation, or pressure support (in room air, or low flow oxygen < 1.1 L/min, or ambient oxygen)).

16. **Sensitivity analysis**

PARCA-R questionnaires completed outside of the range of 23.5 to 27.5 months of age with no classification of cognitive and language impairment by the BERC, and PARCA-R questionnaires with >4 missing items on the non-verbal cognition scale, will be treated as missing data with results from the multiple imputation analysis treated as the primary inference. A further sensitivity analysis will be conducted on the primary outcome, excluding those infants whose PARCA-R scores were imputed.

17. **Safety data analysis**

No further safety data was collected post hospital discharge home

18. **Statistical significance**

95% CIs will be used along with two-sided 5% statistical tests of superiority for all pre-specified outcome comparisons including subgroup analyses.

19. **Procedure for accounting for missing data**

Missing data will be described, by presenting the number of individuals in the missing category.

Standardised PARCA-R scores for non-verbal cognitive and language impairment cannot be calculated for infants whose questionnaires were completed outside of the age range 23.5 to 27.5 months, although raw scores may be available. Standardised PARCA-R scores for non-verbal cognitive impairment cannot be calculated for infants with 4 or more missing

questions. It is assumed that these standardised scores are missing at random, with a multiple imputation analysis to be performed on the PARCA-R assessment scores, imputing standardised scores for these infants (see section 13.1).

The other components of neurodevelopment impairment assessed using PARCA-R, including motor, hearing and visual function, are not restricted by age in the same way. Therefore, imputation for these scores will not be performed.

20. **Deviation from the protocol**

Minor changes to definitions and additions of variables absent from the protocol appeared in this SAP. The secondary long term outcome named in the protocol as survival is defined here as death, to aid interpretation of the individual components of the main long-term outcome. This will not impact the outcome of the statistical test. Moderate or severe neurodevelopmental impairment in survivors, and moderate or severe respiratory morbidity in survivors were additional variables defined in this SAP not included in the protocol.

The pre-specified subgroup analyses outlined in the protocol were only identified as part of the short-term outcomes analysis. These same sub-group analyses will be performed on the main long-term outcome as well.

Imputing missing data for those children with 4 or more missing questions in the non-verbal cognitive subscale was not specified in the protocol, however this is a standard approach recommended for the use of PARCA-R.

21. **Statistical software employed**

The statistical software Stata version 17 (or later) for Windows will be used for all analyses and R version 4.2.3 (or later) will be used for figures.

22. **Additional exploratory analysis**

Any additional analyses not specified in the analysis protocol will be exploratory in nature and will be documented in a separate statistical analysis plan. Any post-hoc analysis requested by a steering committee, investigator, journal editor or referees will be clearly labelled as such.

23. **Dummy tables**

Dummy tables are provided in a separate document.

24. **Health economic analysis**

The health economic analysis is described in a separate analysis plan.

25. **References**

1. Johnson S, Bountziouka V, Linsell L, Brocklehurst P, Marlow N, Wolke D, Manktelow B. Parent Report of Children's Abilities – Revised (PARCA-R). Technical and Interpretive Manual. University of Leicester, Leicester, 2019
2. White IR, Royston P, Wood AM. Multiple imputation using chained equations: issues and guidance for practice. Statistics in medicine. 2011 Feb 20;30(4):377-99.
3. Morris TP, White IR, Royston P. Tuning multiple imputation by predictive mean matching and local residual draws. BMC medical research methodology. 2014 Dec;14:1-3.

26. **Additional documents**

Charter for Blinded Endpoint Review Committee follow up data_Final_v1.0.

27. **Approval**

Senior Statistician	Name: Heather O'Connor	
	Signature	Date
Chief Investigator	Name: Samir Gupta	
	Signature	Date
Chair of Trial Steering Committee (or delegate)	Name: Michael Weindling	
	Signature	Date

Summary of all SAP amendments

There have been no amendments made to the SAP.

Version 1.0 20 Apr 2023 remains the first and current version
