# Supplementary material for: Two-year outcomes after selective early treatment of patent ductus arteriosus with ibuprofen in preterm babies: follow-up of Baby-OSCAR–a randomised controlled trial
Source: eClinicalMedicine. 2025 Aug 20;87:103424. doi: 10.1016/j.eclinm.2025.103424 (PMC12396396; doi:10.1016/j.eclinm.2025.103424)
Supplement: Charter for BERC [file mmc3.pdf]

Full Title: Outcome after Selective Early Treatment for Closure of Patent Ductus ARteriosus in Preterm Babies

Chief Investigator: Professor Samir Gupta

ISCTRN: 84264977
EudraCT No: 2013-005336-23
REC reference: 14/EM/0172

Blinded Endpoint Review Committee (BERC) Charter

Version 1.0 13 December 2021

Contributors:

Dr. Samir Gupta, Chief Investigator
Dr. Samantha Johnson, Co-Investigator
Dr. Pollyanna Hardy, Director of Clinical Trials Unit
Kayleigh Stanbury, Head of Operations
Andy King, Head of Trials Programming
David Murray, Senior Trials Programmer
Clare Edwards, Trial Manager

Baby OSCAR
Outcome after selective early closure of PDA

Contents

1. Name and ID of trial 2
2. Purpose of Blinded Endpoint Review 2
3. Scope of charter 2
4. Qualifications and remit of Blinded Endpoint Review Committee members 3
5. BERC scope and workflow 3
6. Endpoint definitions 5
7. Endpoint data capture 5
Document Sign Off 6
Document History 6
Appendix 1. Classification of 2-year outcome from clinical information and parent questionnaire 7
Appendix 2. Examples of Badgernet report forms 12

1. Name and ID of trial

Full title: Outcome after Selective Early Treatment for Closure of Patent Ductus ARteriosus in Preterm Babies
ISRCTN: 84264977
REC reference: 14/EM/017
Sponsor: University of Oxford
Funder: NIHR Health Technology Assessment programme (HTA)

2. Purpose of Blinded Endpoint Review

A Blinded Endpoint Review Committee (BERC) is a panel of experts with the role of centrally reviewing and classifying endpoints in a blinded and objective fashion, determining whether they fulfil the trial protocol definition, and to harmonise and standardise endpoint assessments. The endpoint review process is intended to enhance the consistency, validity and integrity of the trial's endpoints and/or outcome measures.

3. Scope of charter

This document describes the membership, terms of reference, roles, responsibilities, authority, decision-making and relationships of the Blinded Endpoint Review Committee (BERC) for data relating to 2-year neurodevelopmental outcomes for the Baby-OSCAR trial (see Section 4). It documents the review process, outcome definitions and review conventions to be used during endpoint review.

4. Qualifications and remit of Blinded Endpoint Review Committee members

The BERC will be chaired by Professor Samir Gupta who will take overall responsibility for the endpoint review process. A schedule of agreed reviewers will be maintained, and where additional reviewers are added, a new version of the BERC Charter and committee schedule will be produced, naming a BERC member in the schedule of committee members and authorised by the Chair prior to them undertaking BERC duties.

BERC reviewers will be professionals who are expert in the fields of conditions for which endpoint data is being collected for analysis. The reviews performed will be compliant with the Data Protection Act. Reviewers are not required to be independent of the trial as reviews will be carried out blinded to allocation; the potential for bias in the absence of knowledge of the allocation is considered minimal. Participation in the trial as an investigator at a participating site will not preclude membership of the BERC as a reviewer. The reviewers will be deemed to have no financial conflicts of interest other than being a trial co-investigator.

The composition of the BERC will be as follows:

Name	Role	Expertise
Professor Samir Gupta	Chair of BERC and Chief Investigator of Baby-OSCAR	Professor of Neonatology with expertise in assessing 2-year neurodevelopmental outcomes
Professor Samantha Johnson	Reviewer and co-investigator	Developmental psychologist and Professor of Child Development with expertise in the assessment of neurodevelopmental outcomes
Dr. Nimish Subhedar	Reviewer and co-investigator	Consultant Neonatologist with expertise in assessing 2-year neurodevelopmental outcomes

5. BERC scope and workflow

Participant data will be reviewed if any of the following criteria are met:

- Children for whom a 2-year Baby-OSCAR questionnaire was not completed by a parent or carer.

- Children for whom a 2-year Baby-OSCAR questionnaire was completed outside of the timeframe of 23.5 to 27.5 months corrected age required for deriving PARCA-R standard scores.
- Children for whom there are missing data on questionnaire items precluding classification of one or more of the individual components of the main 2-year outcome

Reviews will be conducted as follows:

- Professionals will review the available data on developmental outcome for each child.
- Each case will be reviewed independently by two professionals with expertise in neurodevelopmental outcome assessment.
- The two reviewers will then compare reviews and determine whether there is agreement in classification of the main outcome and its individual components (see Section X) or whether further review is required.
- The decision made will be recorded on the BERC review form supplied in the case pack provided by the CTU.
- If agreement between both reviewers is reached, the case is complete.
- If a discrepancy occurs but consensus can be reached with discussion between the two reviewers, the case is complete.
- If a discrepancy occurs but agreement cannot be reached with discussion between the two reviewers, the case will be referred to a third reviewer with expertise in assessing neurodevelopmental outcomes. The third reviewer will review the available data independently as well as the two assessment forms completed by the reviewers. The assessment of the 3rd reviewer will be the final decision regarding classification of outcome and the case completed.
- If the two reviewers are unable to classify outcome as there is too much missing data, this will be indicated on the form and the case closed.
- Results of the BERC will not be fed back to the site Principal Investigator as the endpoint was not originally derived at site level.

Decisions will be documented on paper or electronic forms generated for the review and will be signed and dated by each professional undertaking the review. The information from the paper documents will then be added to the Baby-OSCAR OpenClinica database by a member of the trial team based at NPEU CTU following the data entry SOP DM108.

The trial office at NPEU CTU will prepare a review pack for each participant requiring a review. Trial staff will ensure that there is no information identifying the infant or the treatment group the participant was randomised to.

Actions following review:

- If Reviewer 1 and 2 are IN EXACT AGREEMENT:
  The information on the Reviewer 1 form will be entered into the OpenClinica database

  If Reviewer 1 and 2 are NOT in exact agreement BUT A CONSENSUS IS REACHED: The Final Review / Arbitration Form will be completed, signed and dated by Reviewer 1 and Reviewer 2. The information from this form will be entered into the OpenClinica database.

- If Reviewer 1 and Reviewer 2 are NOT in agreement AND NO CONSENSUS IS REACHED:
  The review pack and the two reviews will be independently reviewed by a third Reviewer who will complete, sign and date the Final Review / Arbitration Form. The information on this form will be entered into the clinical database.

If reviewers indicate that there is not enough information available to reach a consensus, trial staff may approach sites to source additional data if they believe there is a reasonable chance that this would be available. If any new data is sourced, the review pack will be generated again with this additional data included, and the case re-reviewed. In the event of no further data being available, the participant will be marked as lost to follow-up.

6. Endpoint definitions

For the purposes of the blinded endpoint review of data relating to neurodevelopment at 2 years, the following outcomes will be considered:

- Moderate or severe neurodevelopmental impairment at 24 months of age corrected for prematurity. This is the main outcome.
- Individual components required to classify the main outcome, as follows:
  - Moderate or severe cognitive impairment
  - Moderate or severe language impairment
  - Moderate or severe hearing impairment
  - Moderate or severe visual impairment
  - Moderate or severe gross motor impairment

The definitions and classifications of all outcomes using parent-reported and routine clinical data are shown in Appendix 1.

7. Endpoint data capture

Parents of all surviving participants are sent a study questionnaire two weeks before their child reaches 24 months of age (corrected for prematurity). The study questionnaire includes the Parent Report of Children's Abilities-Revised (PARCA-R), a standardised parent-report questionnaire that assesses children's cognitive and language development at age 23.5 to 27.5 months, and items to assess children's vision, hearing and gross motor function. Where parents do not return the

questionnaire, the outcomes will be classified using clinical data as outlined in Appendix 1. Data will be obtained from clinical records held by the child's clinical neonatal team. Trial staff will request that, where possible, this is in the form of a summary downloaded from routine data collection sources (BadgerNet) providing an outline of the results of the child's two-year follow-up assessment, or a completed proforma based on the National Neonatal Audit Programme (NNAP) form, including any clinic letters where available. [See Appendix 2 for examples of forms.]

Document Sign Off

	NAME	TITLE	SIGNATURE	DATE
**Author:**	Clare Edwards	Baby-OSCAR Trial Manager		
**Approved by:**	Professor Samir Gupta	Baby-OSCAR Chief Investigator		
**Approved by:**	Professor Samantha Johnson	Co-Investigator		
**Approved by:**	Kayleigh Stanbury	Head of Operations		
**QA sign off:**	Mrs Joy Wiles	Quality Assurance Manager		

Document History

Version	Date	Edited by	Comments
0.1	12/10/21	CE	First draft version
0.5	9/11/21	CE	Addition of Charter to BERC guidelines
0.6	25/11/21	CE	Addressing comments and changes made by contributors
0.9	06/12/2021	AK	Minor updates to formatting
0.10	13/12/2021	CE	Incorporating an extra criteria to add infants where the questionnaire has been completed but data is not complete.

Appendix 1. Classification of 2-year outcome from clinical information and parent questionnaire

Domain and Source of information	No/mild impairment	Moderate impairment	Severe impairment
**COGNITIVE FUNCTION:**<br>**BAPM 2008 criteria**	**-**	**Score -2SD to -3SD below norm (DQ 55-70)**	**Score < -3 SDs below norm (DQ < 55)**
**Parent report**: PARCA-R	PARCA-R non-verbal cognition scale standard score ≥ -2 SD (score ≥ 70)	PARCA-R non-verbal cognition scale standard score <-2 SD to -3 SD (score 55 to 69)	PARCA-R non-verbal cognition scale standard score < –3 SD (score < 55)
**Clinical information**: Bayley-III test score	Bayley-III Cognitive Scale Composite score ≥ -2 SD (adjusted for inflated norms)† score ≥ 80	Bayley-III Cognitive Scale Composite score <-2 SD to -3 SD (adjusted for inflated norms)† score 65 to 79	Bayley-III Cognitive Scale Composite score < –3 SD (adjusted for inflated norms)† score < 65
**Clinical information**: Other standardised cognitive test score	Cognitive test standard score ≥ -2 SD (score ≥ 70)	Cognitive test standard score <-2 SD to -3 SD (score 55 to 69)	Cognitive test standard score < –3 SD (score < 55)
**Clinical information**: Clinical assessment	No evidence of difficulties with cognitive function<br>*Or*<br>Child's development is 3-6 months behind corrected age - Yes (8a)<br>*And*<br>8b and 8c are coded No	Child's development is 6-12 months behind corrected age - Yes (8b)<br>*And*<br>8c is coded No	Development is more than 12 months behind corrected age - Yes (8c)

Baby OSCAR

Outcome after selective early closure of PDA

**LANGUAGE & COMMUNICATION:**<br><br>**BAPM 2008 criteria**	**-**	**Some but fewer than 5 words or signs**<br><br>***Or***<br><br>**Unable to comprehend un-cued command but able to comprehend a cued command.**	**No meaningful words or signs**<br><br>***Or***<br><br>**Unable to comprehend cued command (ie commands only understood in familiar situation or with visual cues (eg gestures).**
**Parent report**: PARCA-R	PARCA-R language scale standard score ≥ -2 SD (score ≥ 70)	PARCA-R language scale standard score <-2 SD to -3 SD (score 55 to 69)	PARCA-R language scale standard score < –3 SD (score <55)
**Clinical information**: Bayley-III test score	Bayley-III Language Scale Composite score ≥ -2 SD (adjusted for inflated norms)† score ≥ 80	Bayley-III Language Scale Composite score <-2 SD to -3 SD (adjusted for inflated norms)† score 65 to 79	Bayley-III Language Scale Composite score < –3 SD (adjusted for inflated norms)† score < 65
**Clinical information**: Other standardised language test score	Language test standard score ≥ -2 SD (score ≥ 70)	Language test standard score <-2 SD to -3 SD (score 55 to 69)	Language test standard score < –3 SD (score < 55)
**Clinical information**: Clinical assessment	Does this child have any difficulty with communication? – Yes (10a)<br><br>*Or*<br><br>Does this child have difficulty with speech (<10 words/signs)? – Yes (10b)<br><br>*Or*<br><br>Does this child have difficulty with understanding outside of familiar context? – Yes (10d)<br><br>*And*<br><br>10c and 10e are coded No	Does the child have <5 meaningful words, vocalisations or signs? - Yes (10c)<br><br>*And*<br><br>10e is coded No	Is this child is unable to understand words or signs? - Yes (10e)

**VISION:**<br>**BAPM 2008 criteria**	-<br>-	**Seems to have moderately reduced vision but better than severe visual impairment**<br>***Or***<br>**Blind in one eye with good vision in the contralateral eye**	**Blind**<br>***Or***<br>**Can only perceive light or light reflecting objects**
**Parent report**: Question A2	No difficulties<br>*Or*<br>Needs to wear glasses but sees well wearing them	Has difficulty seeing, even when wearing glasses<br>*Or*<br>Is blind in one eye but has good vision in the other eye	Is able to see light only or is blind
**Clinical information**: Clinical assessment	No evidence of visual impairment<br>*Or*<br>Does this child have any visual problems (including squint)? – Yes (9d)<br>*And*<br>9e and 9f are coded No	Does this child have visual defect that is not fully correctable? – Yes (9e)<br>*And*<br>9f is coded No	Is this child blind or sees light only? – Yes (9f)
**HEARING:**<br>**BAPM 2008 criteria**	-	**Hearing loss corrected by aids (usually moderate 40-70dBHL)**<br>**Or**<br>**Some hearing loss but not corrected by aids (usually severe 70-90dBHL)"**	**No useful hearing even with aids (profound >90dBHL)**

**Parent report**: Question A3	No difficulties<br>*Or*<br>Has some difficulty hearing but does not need a hearing aid or cochlear implant	Has a cochlear implant or hearing aid, but hears well with it	Has difficulty hearing, even with a cochlear implant or hearing aid<br>*Or*<br>My child is deaf
**Clinical information**: Clinical assessment	No evidence of hearing impairment<br>*Or*<br>Does this child have a hearing impairment? – Yes (9a)<br>*And*<br>9b and 9c are coded No	Does this child have hearing impairment corrected by aids? – Yes (9b)<br>*And*<br>9c is coded No	Does this child have hearing impairment not correctable with aids? – Yes (9c)
**GROSS MOTOR:**<br>**BAPM 2008 criteria**	-	**Cerebral Palsy with GMFCS level 2**	**Cerebral Palsy with GMFCS level 3, 4 or 5**
**Parent report**: Questions A4 & A5	No difficulties walking alone<br>*Or*<br>No difficulties sitting alone<br>*Or*<br>Can walk a few steps without help<br>*Or*<br>Can sit alone but is unstable (may need to use his or her hands for support)	Can only walk if helped by an adult or walking aid<br>*Or*<br>Can only sit with support or with help from an adult	Unable to walk even with help<br>*Or*<br>Unable to sit

**Clinical information**: Bayley-III test score	Bayley-III Motor Scale Composite score ≥ -2 SD (adjusted for inflated norms)† score ≥ 80	Bayley-III Motor Scale Composite score <-2 SD to -3 SD (adjusted for inflated norms)† score 65 to 79	Bayley-III Motor Scale Composite score < –3 SD (adjusted for inflated norms)† score < 65
**Clinical information**: Other standardised motor test score	Motor test standard score ≥ -2 SD (score ≥ 70)	Motor test standard score <-2 SD to -3 SD (score 55 to 69)	Motor test standard score < –3 SD (score < 55)
**Clinical information**:<br><br>Clinical assessment	No evidence of neuromotor impairment<br><br>*Or*<br><br>Does this child have any difficulty walking? – Yes (1a)<br><br>*And*<br><br>1b, 1c, 1d and 1e are coded No	Is this child's gait non-fluent or abnormal reducing mobility?- Yes (1b)<br><br>*Or*<br><br>Is this child unstable or needs to be supported when sitting? – Yes (1d)<br><br>*And*<br><br>1c and 1e are coded No	Is this child unable to walk without assistance – Yes (1c)<br><br>*Or*<br><br>Is this child unable to sit? – Yes (1e)
**Clinical information**:<br><br>Gross Motor Function Classification System (GMFCS)	No evidence of neuromotor impairment<br><br>*Or*<br><br>GMFCS Level 1	GMFCS Level 2	GMFCS Level 3 to 5

Baby OSCAR
Outcome after selective early closure of PDA

Appendix 2. Examples of Badgernet report forms

Update

Assessment

Was 2 year examination done	Yes
Date of assessment	02 Aug 19
Name of person completing form	
Designation	Associate specialist
Child's current name	
Full current postcode	

Neuromotor

Does the child have difficulty walking	No
Is the child's gait non-fluent or abnormal reducing mobility	No
Is child unable to walk without assistance	No
Is child unstable or needs to be supported when sitting	No
Is child unable to sit	No
Does this child have difficulty with the use of one hand	No
Does child have difficulty with the use of both hands	No
Is child unable to use hands to feed self	No

Malformations

Malformation at birth or within first 2 years	Yes
Malformation impairs daily activities despite assistance	No

Social

Is child on at-risk register, fostered or adopted	No

Resp / CVS

Respiratory / CVS system

Limited exercise tolerance with or without treatment	No
On supplemental oxygen or any respiratory support	No

Gastro-intestinal Tract

Is child on a special diet	No

Renal

Renal impairment, no treatment	No

Neurology

Has the child had a seizure or fit in the past 12 months	No
Is the child on anticonvulsants	No
More than 1 seizure a month despite treatment	No
VP shunt ever inserted or in situ	No

Growth

Date of measurements	02 Aug 19
Weight	12.10, kg
Length / Height	89.30, cms
Measured how?	Standing,
Head circumference	48.80, cms

Development score

Is development normal (<3 months delay)	Yes
Is there mild delay (3-6 month delay)	No
Is there moderate delay (6-12 month delay)	No
Is there severe delay (more than 12 month delay)	No
Was child difficult to test	No
Will you refer for detailed neurodevelopmental assessment	No
Has child had a detailed neurodevelopmental assessment	No
What developmental test(s) used	Bayley III

Bayley III

Cognitive	60, , 8, , 22, , 90, , 25, , 83-99
Receptive language	22, , 7, , 19
Expressive language	29, , 9, , 23
Sum	, , 13, , , , 79, , 8, , 73-88
Fine motor	43, , 13, , 29
Gross motor	54, , 8, , 20
sum	, , 21, , , , 103, , 58, , 92-110
Notes	NORMAL GROWTH AND DEVELOPMENT

Auditory	
Does child have a hearing impairment	No
Hearing impairment corrected by aids	No
Hearing impairment, uncorrected by aids	No

Vision	
Any visual problems. inc squint	No
Visual defect not fully correctable	No
Child blind or sees light only	No

Communication	
Does child have difficulty with communication	No
Does child have difficulty with speech	No
Does child have <5 words, vocalisations or signs	No
Unable to understand word or sign out of familiar context	No
Unable to understand words or signs	No

Neurological diagnosis	
Does child have a diagnosis of cerebral palsy	No
Other diagnosis notes	NORMAL GROWTH AND DEVELOPMENT

Baby OSCAR

Outcome after selective early closure of PDA

ADAPTED NNAP 2–YEAR CORRECTED AGE OUTCOME FORM

PLEASE DO NOT COMPLETE THIS FORM IF THE CHILD IS ACUTELY ILL

Hospital of Birth: <Hospital>

Infant's name: <name> Infant's NHS No: <NHS No>

Date of Birth: <DOB> Sex: <Male / Female> Birth weight: <weight>

Name & Designation of person completing form:

____

Date of assessment: _____ / _____ / _____

Reason if child not assessed (delete as applicable): Deceased post discharge / Lost to follow up

Full Current Post Code: _________________ Date of death if applicable:_____ / _____ / _____

Current hospital of follow up:

____

Paediatrician if known:

____

**1. Neuromotor**	**No**	**Yes**	**Don't Know**
a. Does this child have any difficulty walking?			
b. Is this child's gait non-fluent or abnormal reducing mobility?			
c. Is this child unable to walk without assistance?			
d. Is this child unstable or needs to be supported when sitting?			
e. Is this child unable to sit?			
f. Does this child have any difficulty with the use of one hand?			
g. Does this child have difficulty with the use of both hands?			
h. Is this child unable to use hands (i.e. to feed)?			
**2. Development**			
a. Is the child's development between 3-6 months behind corrected age?			
b. Is the child's development between 6-12 months behind corrected age?			
c. Is the child's development more than 12 months behind corrected age?			
d. Will you be referring the child for a detailed neurodevelopmental assessment?			
e. If child had detailed neurodevelopmental assessment, provide name of the test:			
**3. Neurosensory**			
a. Does this child have a hearing impairment?			
b. Does this child have hearing impairment corrected by aids?			
c. Does this child have hearing impairment not correctable with aids?			
d. Does this child have any visual problems (including squint)?			

Baby OSCAR
Outcome after selective early closure of PDA

e. Does this child have visual defect that is not fully correctable?			
f. Is this child blind or sees light only?			
**4. Communication**			
a. Does this child have any difficulty with communication?			
b. Does this child have difficulty with speech (<10 words/signs)?			
c. Does the child have <5 meaningful words, vocalisations or signs?			
d. Does this child have difficulty with understanding outside of familiar context?			
e. Is this child unable to understand words or signs?			

Note: If answering 'yes' to questions 1a - 1h or 2e please classify/enter score on the reverse of this form

1) Does this child have Cerebral Palsy?

Yes	No

If yes, please classify:

Spastic bilateral: 2 limb involvement	
Spastic bilateral: 3 limb involvement	
Spastic bilateral: 4 limb involvement	
Hemiplegia: Right sided	
Hemiplegia: left sided Dyskinetic/ dystonic/ choreo-athetoid	
Not classifiable	

2) Please give diagnosis:

____

Baby OSCAR
Outcome after selective early closure of PDA

Developmental test results if available

We are not requesting a test to be done, but if it has, the results would be helpful

**Bayley III (if performed)** – please enter standardised scores	
Cognitive - Composite score	
Receptive Communication - Scaled score<br>Expressive Communication – Scaled score	
Language – Composite score	
Fine Motor – Scaled score<br>Gross Motor – Scaled score	
Motor – Composite score	
Notes	

**Griffiths (if performed)** – please enter standardised scores	
A Locomotor<br>B Personal and social<br>C Hearing and Language<br>D Eye and hand coordination	
E Performance	
F Practical reasoning	
Notes	

**Schedule of Growing Skills (if performed)** – please enter standardised scores	
Locomotor<br>Manipulative	
Interactive social	
Self-care social<br>Hearing and Language	
Speech and Language	
Visual	
Notes	
